# A Landscape Analysis of Pediatric and Congenital Heart Disease Services in Africa

**DOI:** 10.1177/21501351251316230

**Published:** 2025-08-21

**Authors:** Thomas Aldersley, Sulafa Ali, Adila Dawood, Frank Edwin, Kathy Jenkins, Alexia Joachim, John Lawrenson, Darshan Reddy, Drissi Boumzebra, James D. St. Louis, Christo Tchervenkov, Amy Verstappen, Bistra Zheleva, Liesl Zühlke, On behalf of all respondents

**Affiliations:** 1 Department of Paediatrics, 37716 University of Cape Town, Cape Town, South Africa; 2 Division of Pediatric Cardiology, 89235 University of Khartoum, Khartoum, Sudan; 3 Division of Cardiothoracic Surgery, 549574 University of Health and Allied Sciences, Ho, Ghana; 4 Department of Cardiology, Harvard Medical School Department of Pediatrics, 1862 Boston Children's Hospital, Boston, MA, USA; 5 Department of Paediatrics, Stellenbosch University, Stellenbosch, South Africa; 6 Division of Cardiothoracic Surgery, University of Kwa-Zulu Natal, Durban, South Africa; 7 Department of Cardiovascular Surgery, Mohamed VI University Hospital, Marrakech, Morocco; 8 Department of Surgery, 1421 Augusta University, Augusta, GA, USA; 9 Division of Pediatric Cardiovascular Surgery, McGill University, Montreal, Canada; 10 Global Arch, Windsor, CT, USA; 11 Children's Heart Link, Minneapolis, MN, USA; 12 59097 South African Medical Research Council, Cape Town, South Africa

**Keywords:** pediatric, cardiology, cardiac surgery, global health, health infrastructure, services, Africa, survey

## Abstract

**Background:**

There is geographic disparity in the provision of Pediatric and Congenital Heart Disease (PCHD) services; Africa accounts for only 1% of global cardiothoracic surgical capacity.

**Methods:**

We conducted a survey of PCHD services in Africa, to investigate institution and national-level resources for pediatric cardiology and cardiothoracic surgery. Results were compared with international guidelines for PCHD services and institutions were ranked by a composite score for low- and middle-income PCHD services.

**Results:**

There were 124 respondents from 96 institutions in 45 countries. Eighteen (40%) countries provided a full PCHD service including interventional cardiology and cardiopulmonary bypass (CPB) cardiac surgery. Ten countries (22%) provided cardiac surgery services but no interventional cardiology service, 4 of which did not have CPB facilities. One provided interventional cardiology services but no cardiac surgery service. Ten countries (22%) had no PCHD service. There were 0.04 (interquartile range [IQR]: 0.00-0.13) pediatric cardiothoracic surgeons and 0.17 (IQR: 0.02-0.35) pediatric cardiologists per million population. No institution met all criteria for level 5 PCHD national referral centers, and 8/87 (9.2%) met the criteria for level 4 regional referral centers. Thirteen (29%) countries report both pediatric cardiology and cardiothoracic surgery fellowship training programs.

**Conclusions:**

Only 18 (40%) countries provided full PCHD services. The number of pediatric cardiologists and cardiothoracic surgeons is below international recommendations. Only Libya and Mauritius have the recommended 2 pediatric cardiologists per million population, and no country meets the recommended 1.25 cardiothoracic surgeons per million. There is a significant shortage of fellowship training programs which must be addressed if PCHD capacity is to be increased.

## Introduction

There is geographic disparity in the provision of Pediatric and Congenital Heart Disease (PCHD) services. Previous studies show that North America and Western Europe account for 74% of the world's cardiothoracic surgical capacity. Africa, however, accounts for only 1% of the total global capacity.^1^ This translates to geographic disparities in outcomes for congenital (CHD) and rheumatic heart diseases (RHD). For example, globally RHD deaths have decreased by 15.6% since 1990.^2^ In low-income and middle-income countries (LMICs), however, there was a 23.75% and 7.07% increase in RHD deaths over the same period, respectively.^2^ Similarly, CHD caused 261 247 deaths globally in 2017, a reduction of 34.5% (95% uncertainty interval: 19.8-44.6) since 1990.^3^ However, CHD mortalities decreased by only 20.1% in southern sub-Saharan Africa, and actually increased for all other sub-Saharan regions, with mortalities in Central and Western sub-Saharan Africa increasing by 38.1% and 40.3%, respectively.^3^ These disparities underscore the urgent need to improve PCHD services in Africa and other low- and middle-income settings. To achieve this, we must first understand what level of services currently exists in relation to established PCHD guidelines.

Pediatric and Congenital Heart Disease center guidelines vary depending on their geographic focus, primary medical field, conceptual framework, and consequently their utility in LMICs.^4–6^ The Hasan et al guidelines^4^ conceptualized in a public health framework seek to create an actionable system to integrate PCHD care within existing LMIC health systems. Hasan et al propose a tier-based framework for PCHD care based on the World Bank health facilities classification (levels 1-5), defined by the complexity of care provided. In this system, level 1, 2, and 3 centers handle screening, diagnosis, noninvasive management, and referral. Level 4 centers are regional referral centers and offer a comprehensive pediatric cardiac service including cardiac intensive care, cardiac catheterization (PREDIC3T categories 0-4),^7^ and surgery (RACHS categories 1-2).^8^ Level 5 centers additionally, offer adult CHD (ACHD) care, all cardiac procedures, transplants, left ventricular assist devices (LVADs), and extracorporeal membrane oxygenation (ECMO). Level 4 centers require pediatric cardiologists but can be staffed with either pediatric or adult anesthetists and cardiothoracic surgeons. Level 5 centers, however, require credentialed pediatric cardiothoracic surgeons and cardiac anesthetists. Additionally, Level 4 centers should provide cardiology and cardiothoracic surgery training, while level 5 centers should also offer research programs.

The UK report on cardiac services recommends 2 pediatric cardiologists per million population.^9^ This figure is based on UK demographics and may need to be higher in lower-income settings with higher birth rates and lower median age, resulting in a higher CHD burden. For example, Daenen et al^6^ recommend a minimum of 0.4 (0.33-0.5) cardiothoracic surgeons per million population, whereas Sliwa et al^10^ recommend that African cardiac centers should have 1.25 cardiothoracic surgeons per million population. This recommendation accounts for higher African fertility rates (4.7 in Africa vs 1.6 in Europe)^11^ and is proportionally similar.

This landscape analysis of pediatric cardiac services in Africa seeks to quantify gaps in PCHD service delivery relative to these established guidelines. Understanding where and how these services fall short is essential to informing targeted interventions and strategies aimed at aligning African PCHD services with global best practices, ultimately ensuring equitable access to high-quality cardiac care for all children across the continent.

## Methods

We conducted a cross-sectional electronic survey to evaluate PCHD services in Africa.

### Survey Development and Testing

The survey was developed by the authors belonging to the University of Cape Town's Children's Heart Disease Research Unit in collaboration with Pan-African Network for Pediatric and Congenital Hearts, a special interest group of the Pan-African Society of Cardiology, the African Society for Pediatric and Congenital Heart Surgery and the World Society for Pediatric and Congenital Heart Surgery, the International Quality Improvement Collaborative for Congenital Heart Disease, Children's Heart Link, and Global Alliance for Rheumatic and Congenital Hearts (ARCH).

Following pretesting, the survey was pilot tested with three pediatric cardiologists, one adult cardiologist, and one medical officer for comprehensiveness and clarity.^12^ The individuals comprising the test group were deliberately selected to align with the expected professional diversity of the survey respondents.

The survey was developed in English only and consisted of 138 possible questions. Branching logic was used to reduce respondent burden by hiding irrelevant questions, and the average completion time was 28 min. The survey included respondent-level, institution-level, and national-level queries. Respondent-level questions included profession, specialization, and practicing institution. Institution-level details included staffing, infrastructure, and services for pediatric cardiology, cardiac catheterization, cardiothoracic surgery, and intensive care, for ACHD, and cardio-obstetrics. The survey also included information related to screening programs, cardiac imaging, electronic health records and databases, fellowship training, and financial organization. National-level questions related to the availability and provision of pediatric cardiology, interventional cardiac catheterization, pediatric cardiothoracic surgery, and fellowship training (see Supplemental File, Appendix 1 for full questionnaire).

### Recruitment and Data Collection

Potential respondents were selected by purposive sampling. A literature review was performed by searching the PubMed/MEDLINE and Google Scholar databases using a combination of pediatric cardiology, cardiology, cardiothoracic surgery, cardiac surgery, Africa, and country-specific keywords. Local African authors were identified and contacted via included contact details or via follow-up contact tracing. An extensive internet search for PCHD practitioners and institutions was also conducted, with a review of hospital, university websites, and professional social network websites, including LinkedIn and ResearchGate. Additionally, the authors leveraged their individual professional networks to contact potential collaborators, drawing on connections to the World Health Organization and other global entities. Potential respondents were contacted via email in English, French, and Portuguese and sent URL and QR-code links to the online survey. Where necessary, follow-up by telephone was conducted in English only.

The study protocol was reviewed and approved by Human Research Ethics Committee, University of Cape Town (HREC459-2023). No personally identifiable staff or patient data were collected, and consent to participate was obtained in the survey preface. Survey distribution was commenced on June first, 2023. All eligible responses received before December first, 2023, were incorporated into the analysis.

### Data Analysis

Data were analyzed using R (version 4.4.0, R Foundation).^13^ The survey permitted multiple respondents per country and institution. These responses were amalgamated to create summaries at both the national and institutional levels. The African countries and regions defined in this study correspond to the 55 member states and five regions recognized by the African Union (AU), as detailed in the AU's official country profiles.^14^

Before combining the data, categorical responses were scrutinized for discrepancies between countries and institutions. If any were discovered, additional questionnaires were distributed for clarification. Likewise, continuous data underwent scrutiny for inconsistencies and outliers. Inconsistencies were addressed through supplementary questionnaires, outliers were removed, and the data were summarized using means or medians based on their distribution.

Institutions were ranked according to a composite score based on Hassan et al's recommendations for developing PCHD services in LMICs.^4^ Variables relating to human resources, PCHD services, infrastructure, training, and health data infrastructure were assessed. A full list of criteria included in the analysis is available in Supplemental Table S1. Radar plots were utilized to visually compare the percentage of institutions meeting criteria for level 4 or 5 PCHD centers across the five categories within each UN African subregion. Radar plots allow for a multidimensional comparison, revealing the relative strengths and weaknesses across categories and subregions. Each axis of the radar plot represents one category, with values ranging from 0% to 100%, indicating the proportion of institutions meeting the specified criteria. Higher values along an axis indicate greater compliance with the criteria, while lower values signify gaps in service provision. The plotted points are then connected to form polygons for each region, asymmetry indicates discrepancies between the different categories and areas of strength or potential targets for intervention. In addition to examining the primary categories, radar plots were also used to analyze specific subcriteria within three key categories: human resources, infrastructure, and PCHD services.

## Results

### Respondents

There were 124 respondents, from 96 institutions, in 70 cities, and 45 different countries in Africa representing 93.7% of the African population. The cumulative population of the 10 countries with nonrespondents amounts to only 6.3% of the total African population.^15^ These 10 countries were Cape Verde, Central African Republic, Djibouti, Equatorial Guinea, Eritrea, Guinea, Ivory Coast, Madagascar, Republic of the Congo, and the Sahrawi Arab Democratic Republic.

Most respondents were interventional pediatric cardiologists (31%, 38/119), followed by noninterventional pediatric cardiologists (26%), pediatricians (12%), pediatric cardiac surgeons (11%), adult cardiologists (7%), adult cardiac surgeons (4%), and pediatric intensivists (2%). Other respondents (6%) included pediatric cardiology and cardiothoracic surgery fellows, general practitioners, and anesthetists.

### National-Level Data

**Figure 1.**
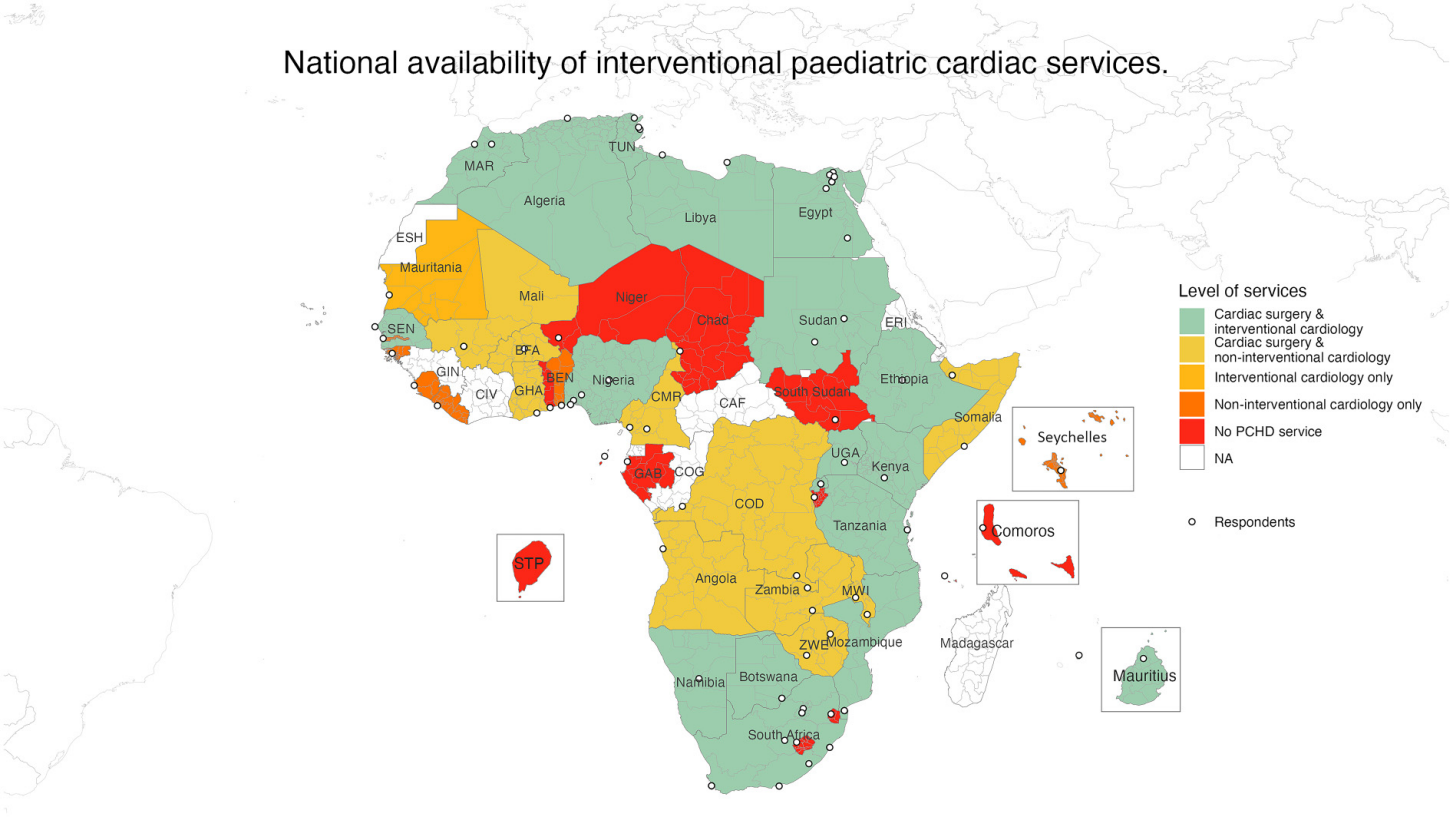
Choropleth depicting availability of cardiac services at the national level. Abbreviations: BEN, Benin; BFA, Burkina Faso; CAF, Central African Republic; CIV, Ivory Coast; CMR, Cameroon; COD, Democratic Republic of the Congo; COG, Republic of the Congo; ERI, Eritrea; ESH, Sahrawi Arab Democratic Republic; GAB, Gabon; GHA, Ghana; GIN, Guinea; MAR, Morocco; MWI, Malawi; SEN, Senegal; TUN, Tunisia; UGA, Uganda; ZWE, Zimbabwe.

Aggregated country-level data showed that 78% or 35 of the 45 respondent countries had some form of cardiac service (Figure 1). Eighteen countries (40%) were able to provide a full PCHD service including interventional pediatric cardiology and pediatric cardiopulmonary bypass (CPB) cardiac surgery (Figure 1, Supplemental File). Ten countries (10/45, 22%) provided pediatric cardiac surgery services but no interventional pediatric cardiology service, of these 4/45 (9%) did not have CPB facilities. Of the 17/45 countries (38%) with no cardiac surgery service, 1 country provided interventional pediatric cardiology services, 6 countries provided a noninterventional cardiology service, and 10 had no PCHD service (Figure 1). Additionally, 49% (22/45) of countries had surgical support from visiting international teams. This included four countries, which rely entirely on visiting teams for surgical interventions, that are not included among the 28 cardiac surgery centers.

#### Pediatric Cardiac Surgery

**Figure 2.**
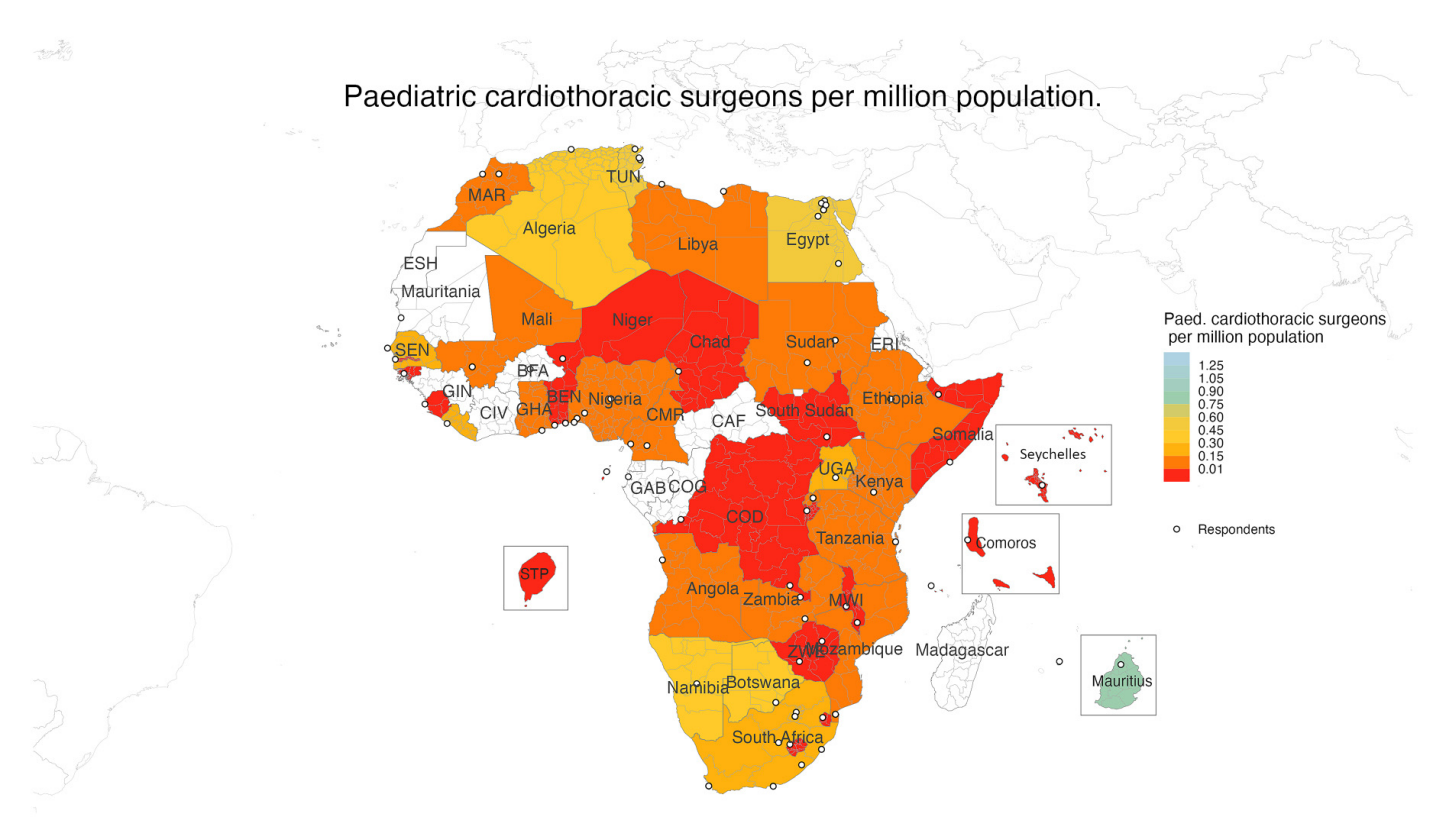
Choropleth depicting the number of pediatric cardiothoracic surgeons per million population at the national level. Abbreviations: BEN, Benin; BFA, Burkina Faso; CAF, Central African Republic; CIV, Ivory Coast; CMR, Cameroon; COD, Democratic Republic of the Congo; COG, Republic of the Congo; ERI, Eritrea; ESH, Sahrawi Arab Democratic Republic; GAB, Gabon; GHA, Ghana; GIN, Guinea; MAR, Morocco; MWI, Malawi; SEN, Senegal; TUN, Tunisia; UGA, Uganda; ZWE, Zimbabwe.

Aggregated country-level data showed a median number of 1 (interquartile range [IQR]: 0-3.0) pediatric cardiothoracic surgeon per country or 0.04 (IQR: 0.00-0.14) pediatric cardiothoracic surgeons per million population, far below the international recommended ratio of 1 pediatric cardiothoracic surgeon per 800,000 population (1.25 per million population) (Figure 2).^10^ Of the 44 respondent countries, 20 (45%) had no pediatric cardiothoracic surgeons, 8 (18%) countries had one surgeon, 10 (23%) countries had one to five surgeons, and 6 (14%) had more than five surgeons.

#### Pediatric Cardiology

**Figure 3.**
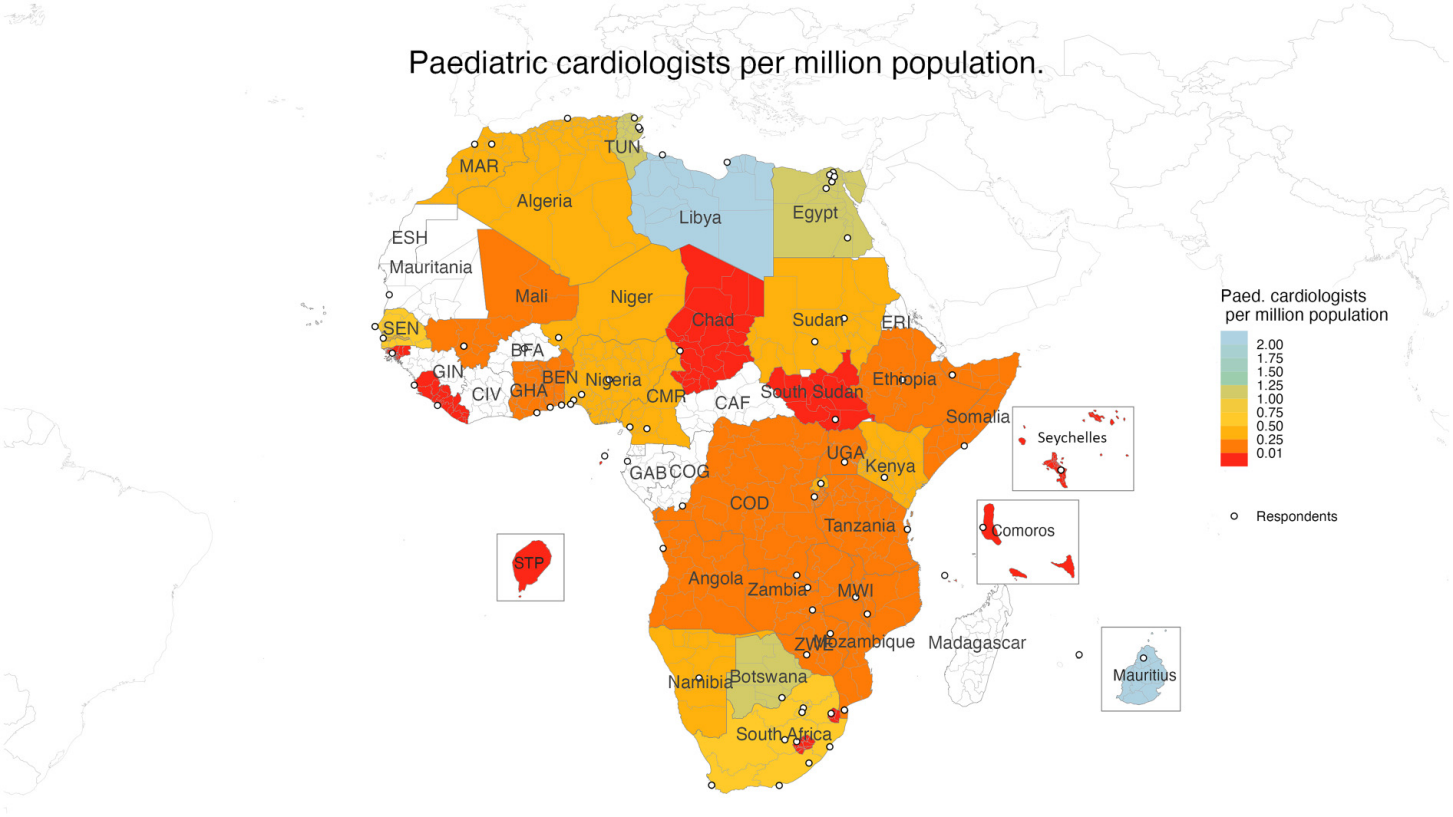
Choropleth depicting the number of pediatric cardiologists per million population at the national level. Abbreviations: BEN, Benin; BFA, Burkina Faso; CAF, Central African Republic; CIV, Ivory Coast; CMR, Cameroon; COD, Democratic Republic of the Congo; COG, Republic of the Congo; ERI, Eritrea; ESH, Sahrawi Arab Democratic Republic; GAB, Gabon; GHA, Ghana; GIN, Guinea; MAR, Morocco; MWI, Malawi; SEN, Senegal; TUN, Tunisia; UGA, Uganda; ZWE, Zimbabwe.

Similarly, aggregated country-level data showed a median number of 3 (IQR: 0.17-10) pediatric cardiologists per country or 0.17 (IQR: 0.02-0.35) pediatric cardiologists per million population, below the international recommended ratio of one pediatric cardiologist per 500,000 population (2 per million population) (Figure 3).^9^ Of the 43 respondent countries, 11 (26%) had no pediatric cardiologists, 5 (12%) countries had one cardiologist, 10 (23%) countries had one to five cardiologists, and 17 (40%) had more than five cardiologists.

### Institutions

Our results include respondents from 96 different hospitals across Africa, 27 institutions in Eastern Africa, 21 in Western Africa, 20 in Northern Africa, 20 in Southern Africa, and 8 in Central Africa.

#### Pediatric cardiac surgery

Pediatric cardiac surgery services were available in 53% (51/96) of institutions; however, only 46 institutions (48%) had cardiac perfusion services, with a median of 2 (IQR: 2-2) CPB machines per hospital. Overall, this equates to a median of 0.04 (IQR: 0.03-0.15) centers offering CPB cardiac surgery per million population (Table 1). Southern Africa has the highest proportion of CPB cardiac surgery centers with 0.175 centers per million population, has a *z*-score of 1.73, the highest positive deviation from the mean rate for all regions, and is the only region outside the range for low-income countries.^16^ Central Africa has the lowest rate of CPB cardiac surgery centers with 0.005 centers per million population and the highest negative deviation from the mean rate (*z*-score: −0.75).

**Table 1.** The Number of Cardiac Surgery Centers With Cardiopulmonary Bypass Services per Million Population Overall and Stratified by AU Subregion.

Region	Number of centers	Number of cardiac surgery centers with cardiopulmonary bypass (CPB) services	Population	Number of cardiac surgery centers with CPB services per million population	*z*-score^a^
All Africa	96	49 (51%)	1 424 810 790	0.034	NA
Northern Africa	20	14 (70%)	259 393 961	0.054	−0.04
Western Africa	21	9 (43%)	429 079 551	0.021	−0.52
Central Africa	8	1 (13%)	196 077 898	0.005	−0.75
Eastern Africa	27	13 (48%)	471 660 692	0.028	−0.42
Southern Africa	20	12 (60%)	68 598 688	0.175	1.73

Abbreviation: AU, African Union; CPB, cardiopulmonary bypass.

^a^ *z*-score indicates how many standard deviations each region's rate is from the mean of all the regions.

Most hospitals had two operating rooms (median 2, IQR: 1-2, range 0-5), with most (58.8%, 30/51) of them being used for pediatric cases only. On average, these centers performed a median of 98 (IQR: 33-150) pediatric CHD surgeries and 20 (IQR: 8-50) pediatric acquired heart disease surgeries annually.

Most of the responding cardiac surgery programs were run by cardiothoracic surgeons with formal training in congenital heart surgery (42/51, 82%). The remaining programs were run by cardiothoracic surgeons with no formal training in congenital heart surgery (5/51, 9.8%) and a combination of general surgeons, pediatric surgeons, and sessional staff (4/51, 7.9%). Of these program leads, 29/51 (57%) had greater than 10 years of experience, 15/51 (29%) had 5 to 10 years’ experience and 6/51 (12%) had less than 5 years’ experience. The median number of formally trained cardiothoracic surgeons in full-time employment per hospital was 2 (IQR: 2-4.8), with a median of 1 (IQR: 1-2) surgeons with formal training in congenital heart disease surgery per hospital. Thirty-four hospitals (34/51, 66.7%) had a pediatric cardiac anesthesiologist, with a median of 2 (IQR: 1-4) pediatric cardiac anesthesiologists per hospital. There was a median of 4.5 (IQR: 3-10) operating room nurses per hospital.

#### Pediatric cardiology

Seventy-five hospitals provided pediatric cardiology services. Forty-four (46%, 44/96) of these performed interventional pediatric cardiac catheterizations, with a median of 37.5 (IQR: 13-100) performed annually. Thirty-one hospitals (32%, 31/96) provided a noninterventional cardiology service. Eighteen hospitals (18/96, 19%) had no PCHD care services.

**Figure 4.**
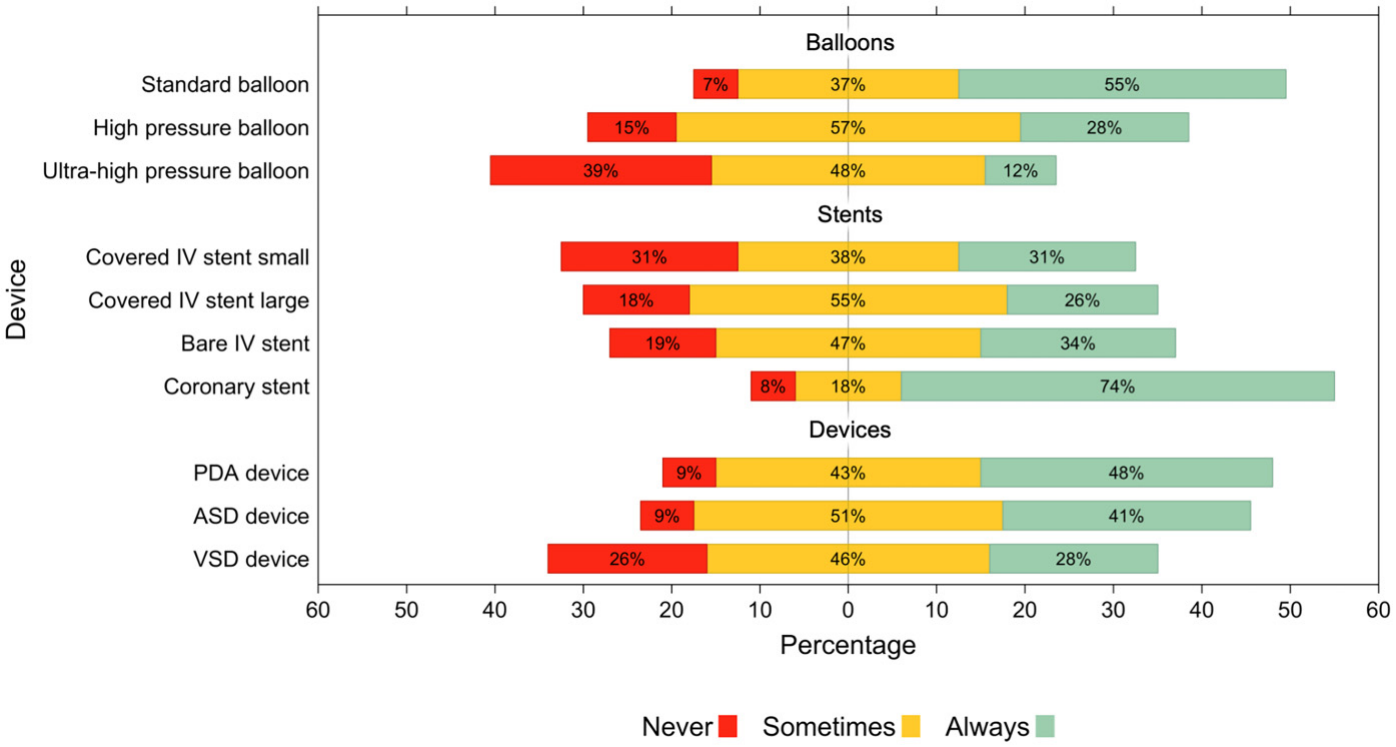
Likert scale depicting availability of cardiac catheterization equipment. Abbreviations: ASD, atrial septal defect; PDA, patent ductus arteriosus; VSD, ventricular septal defect.

Most (90.7%, 68/75) pediatric cardiology services were run by a formally trained pediatric cardiologist, five services were run by general pediatricians, one center was run by a combination of adult and pediatric cardiologists, and one center did not specify. Most of these department heads had more than 10 years’ experience (63%, 47/75), 20/75 (27%) had 5 to 10 years’ experience, 7/75 (9%) had less than 5 years’ experience, and 1 did not specify. The median number of doctors in full-time employment per cardiology service was 4 (IQR: 2-8, max 35); of this median 2 (IQR: 1-4, max 32) were formally trained pediatric cardiologists. Cardiac catheterization laboratories were available for 72% (54/75) of services, of these 89% (48/54) were onsite, 69% (37/54) of these had dedicated surgical backup, 15% (8/54) were used exclusively for pediatric cases, and 56% (30/54) had biplane fluoroscopy machines. There was a median of 4 (IQR: 2-7) catheterization laboratory nurses per hospital. Availability of cardiac catheterization equipment was generally low, especially for specialized equipment such as ultra-high-pressure balloons, small covered vascular stents, and ventricular septal defect (VSD) closure devices. Coronary stents (which may be utilized in pediatric cases) and standard balloons were “always available” in most centers. Similarly, patent ductus arteriosus (PDA) and atrial septal defect (ASD) closure devices were also commonly “always available” (Figure 4).

### Adult CHD and Cardio-Obstetric Services

Pediatric patients were transferred to an adult service at a median of 17 years of age (IQR: 15-18). Dedicated ACHD services were available in 12 centers, in six countries: four centers in Egypt, four centers in South Africa, and one center each in Ethiopia, Guinea-Bissau, Namibia, and Niger (Figure 2, Supplemental File). Cardio-obstetric services were available in nine centers in four countries: four centers in South Africa, two centers in Egypt, two centers in Mozambique, and one center in Sudan (Figure 2, Supplemental File).

### Fellowship Training

**Figure 5.**
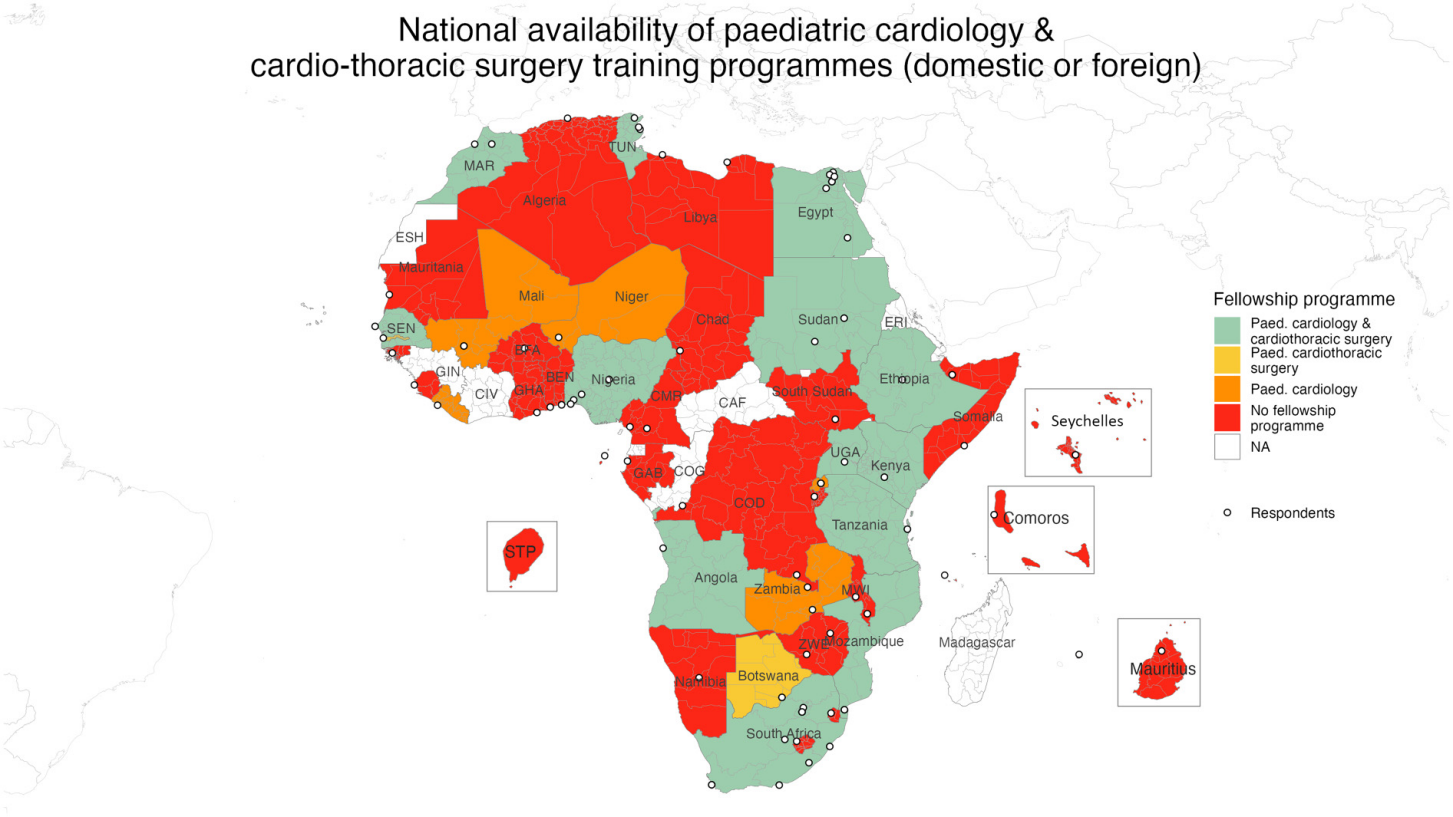
Choropleth depicting the availability of pediatric cardiology and cardiothoracic surgery fellowship programs at the national level. Abbreviations: BEN, Benin; BFA, Burkina Faso; CAF, Central African Republic; CIV, Ivory Coast; CMR, Cameroon; COD, Democratic Republic of the Congo; COG, Republic of the Congo; ERI, Eritrea; ESH, Sahrawi Arab Democratic Republic; GAB, Gabon; GHA, Ghana; GIN, Guinea; MAR, Morocco; MWI, Malawi; SEN, Senegal; TUN, Tunisia; UGA, Uganda; ZWE, Zimbabwe.

More than one-half (25/45, 56%) of the respondent countries reported no pediatric cardiology or cardiothoracic surgery training program. Of the 20 countries with fellowship programs, 13/45 (29%) had both cardiology and cardiothoracic surgery fellowship programs, 5/45 (11%) only had cardiology programs, and 2/45 (4%) only had cardiothoracic surgery fellowship programs (Figure 5). At an institutional level, there were 19 cardiology fellowship programs, most of which trained fellows locally (13/19, 69%), 5/19 (26%) programs trained cardiology fellows both locally and internationally, and 1 program did not specify. Most (18/19, 95%) cardiology programs provided official certification at completion of training, and trained a median of 2 (IQR: 1.5-3, range 1-10) pediatric cardiologists per year. Similarly, there were 19 cardiothoracic surgery fellowship programs which were primarily local (11/19, 58%), with 6/19 (32%) programs training fellows both locally and internationally, 1/19 (5%) program training fellows exclusively internationally, and 1 program not specifying. Most (17/19.90%) cardiothoracic surgery programs provided official certification at completion of training. Each institution trained a median of 3.5 (IQR: 2-4.3, range 1-12) pediatric cardiothoracic surgeons annually.

### Pediatric and Congenital Cardiac Service Levels

**Figure 6.**
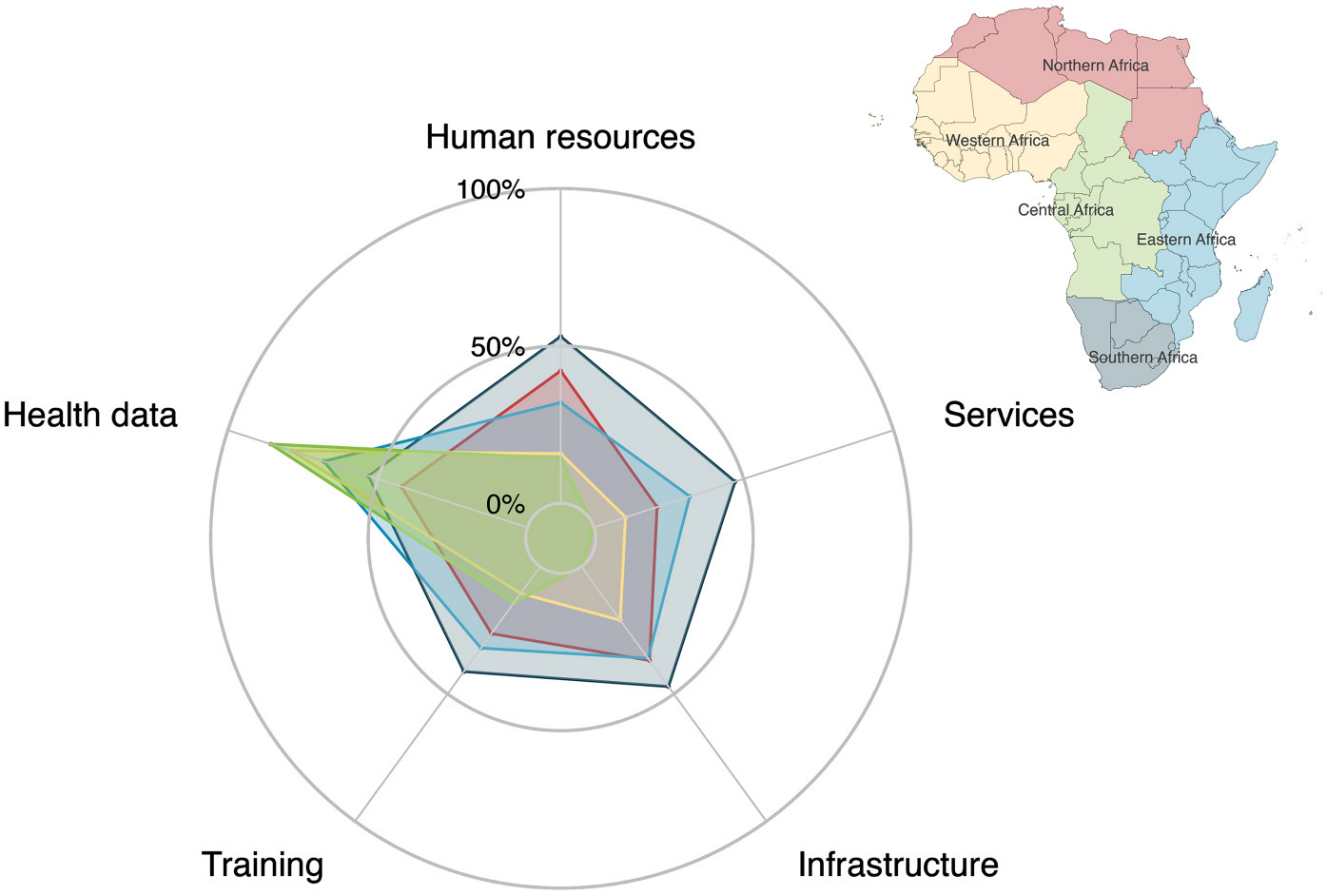
Radar plot showing the percentage of institutions meeting the category criteria for Level 4 or 5 Pediatric and Congenital Heart Disease (PCHD) centres^4^ across African Union (AU) subregions (Northern, Western, Central, Eastern, and Southern Africa). The categories assessed include Human Resources, PCHD Services, Infrastructure, Training, and Health Data Infrastructure. Each axis represents one of these categories, with the plotted points connected to form a polygon for each subregion. The extent of the polygon along each axis indicates the proportion of institutions in that subregion meeting the specific category criteria, with asymmetry indicating imbalance or disparity between the different categories. This allows for a visual comparison of strengths and gaps in PCHD care across the different regions.

**Figure 7.**
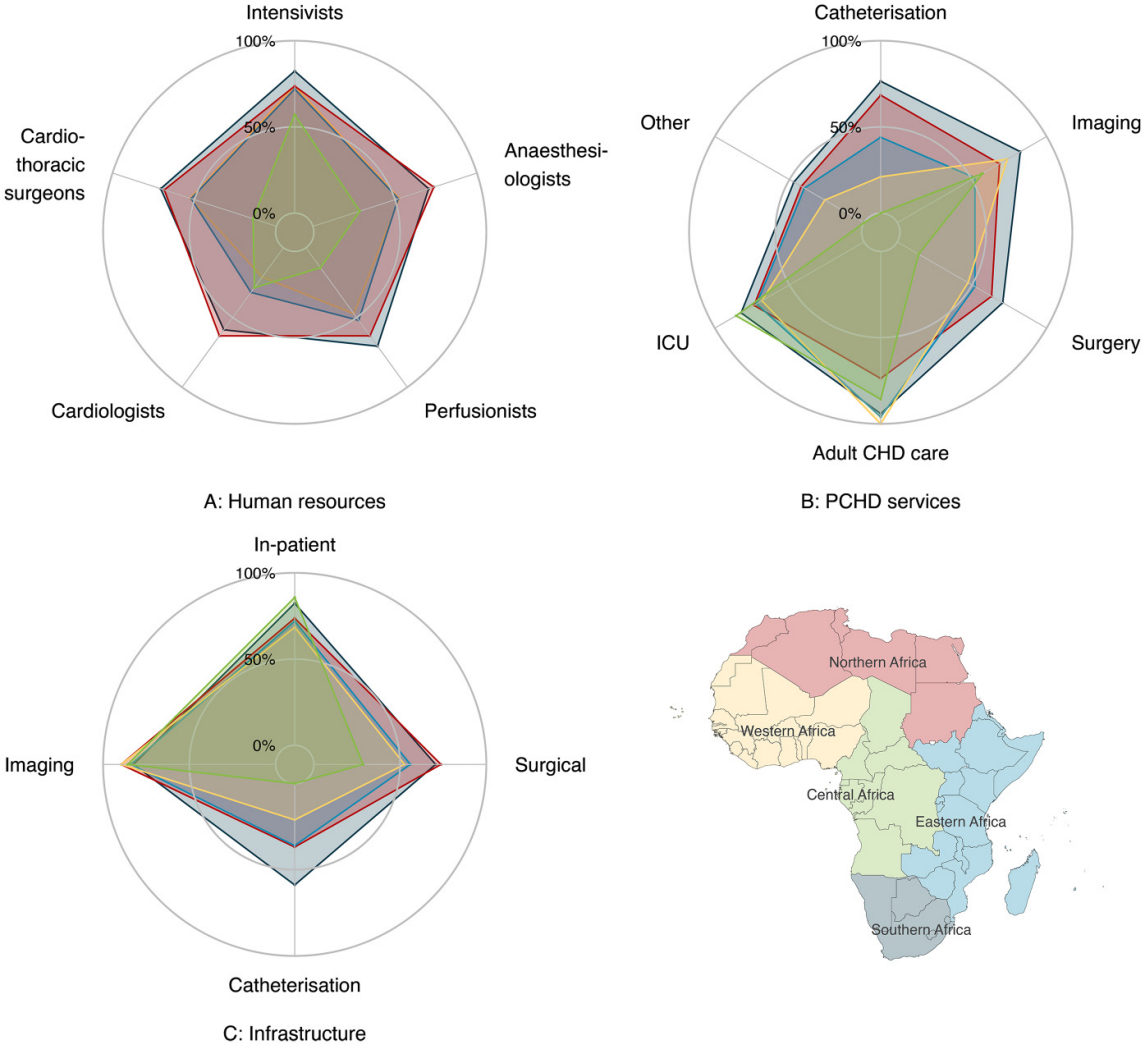
Radar plots showing the percentage of institutions meeting level 4 or 5 Pediatric and Congenital Heart Disease (PCHD)^4^ subcriteria for categories (A) Human resources, (B) PCHD Services, and (C) Infrastructure. Data are stratified by African Union (AU) subregions (Northern, Western, Central, Eastern, and Southern Africa). Each axis represents one of these subcriteria, with the plotted points connected to form a polygon for each subregion. The extent of the polygon along each axis indicates the proportion of institutions in that subregion meeting the specific subcriteria, allowing for a visual comparison of strengths and gaps across the different regions. Abbreviations: CHD, congenital heart disease; ICU, intensive care unit; PCHD, pediatric and congenital heart disease.

The 96 institutions were ranked according to a composite score based on Hasan et al's recommendations for developing PCHD services in LMICs.^4^ Twelve responses, from nine institutions were excluded from this analysis due to missing data. When ranked according to human resources, services, infrastructure, training, and health-data infrastructure, there were no national-level referral centers (Level 5) and only eight regional-level referral centers (Level 4): five from Southern Africa, two from Northern Africa and one from Eastern Africa (Table 2). By category, only 29/87 (33%) of institutions reported having level 4 or 5 human resources, 22/87 (25.3%) of institutions met level 4 or 5 criteria for PCHD services, 28/87 (32.2%) met level 4 or 5 criteria for available infrastructure, 23/87 (26.4%) met level 4 or 5 criteria for PCHD training, and 55/87 (63%) had sufficient health data infrastructure to enable quality control measures to be implemented. Stratification by AU subregion reveals geographic discrepancies. The radar plot in Figure 6 shows the proportion of centers in each region which meet either level 4 or 5 criteria in human resources, services, infrastructure, training, and health data infrastructure. Analysis of the human resources, PCHD services, and infrastructure categories subcriteria, stratified by African Union (AU) subregion (Figure 7) reveal further discrepancies.

**Table 2.** The Proportion of Respondent Health Centers Meeting All Hasan et al^4^ Criteria for Either Level 4 or Level 5 PCHD Centers, Stratified by AU Subregion and Criteria Category.

	Number (N) of institutions	N (%) of level 4/5 institutions	N (%) of level 4/5 institutions by category				
			Human resources	Services	Infrastructure	Training	Health data
Africa	87	8/87 (9.2%)	29 (33.3%)	22 (25.3%)	28 (32.2%)	23 (26.4%)	55 (63.2%)
Northern Africa	19	1/19 (5.3%)	8 (42.1%)	4 (21.1%)	7 (36.8%)	5 (26.3%)	8 (42.1%)
Western Africa	19	0	3 (15.8%)	2 (10.5%)	4 (21.1%)	2 (10.5%)	15 (78.9%)
Central Africa	7	0	1 (14.3%)	0 (0%)	0 (0%)	1 (14.3%)	6 (85.7%)
Eastern Africa	25	2/25 (8.0%)	8 (32%)	8 (32%)	9 (36%)	8 (32%)	17 (68%)
Southern Africa	17	5/17 (29.4%)	9 (52.9%)	8 (47.1%)	8 (47.1%)	7 (41.2%)	9 (52.9%)

Abbreviations: AU, African Union; PCHD, Pediatric and Congenital Heart Disease.

**Table 3.** Summary of Study Findings and List of Recommendations.

Findings	Recommendations	
	Short term	Long term
Severe shortage of pediatric cardiologists and cardiothoracic surgeons. Severe shortage of allied cardiac healthcare practitioners, including perfusionists and anesthetists.	Telemedicine Medical missions International fellowships	Increase government and institutional investment. Better integrate pediatric cardiology and cardiothoracic surgery into general medical curricula, to raise awareness and foster interest. Increase fellowship training programs across Africa. Implement retention strategies. Promote international collaborations and mentorship.
Only 18/45 (40%) countries provide comprehensive PCHD services.	Regional collaborations and cross-border PCHD programs.^17^ Medical missions	Develop existing PCHD centers, focusing on increasing resources for interventional catheterization and cardiac surgery. Consider cross-border PCHD programs.
Insufficient cardiac surgery centers with cardiopulmonary bypass facilities (0.034 centers per million population).	Regional collaborations and cross-border PCHD programs.^17^ Medical missions	Increase fellowship training programs across Africa, emphasizing the need for perfusionist training programs. Develop existing PCHD centers, focusing on increasing resources for cardiopulmonary bypass cardiac surgery. Consider cross-border PCHD programs.
Lack of level 5 (0%) and level 4 (9.2%) cardiac centers.		Support centers in meeting levels 4 and 5 PCHD criteria by addressing gaps in human resources, services, infrastructure, training, and health data infrastructure.
Geographic disparities in PCHD services (Central and Western Africa most underserved)	Regional collaborations and cross-border PCHD programs.^17^ Targeted medical missions.	Implement region-specific development plans, with targeted investments in the most underserved regions (Central and Western Africa), addressing their unique deficiencies in human resources and infrastructure. Foster collaboration between African regions, facilitating knowledge exchange and shared resources to fill specific service gaps.
Fellowship programs lacking, especially in Western and Central Africa	International fellowships.	Replicate and scale successful models like the APFP train-the-trainer program to ensure more locally trained specialists are available to provide PCHD services and to develop local fellowship training programs. Establish more pediatric cardiology and cardiothoracic surgery fellowship programs in countries, especially in underserved regions.
Global health issue: limited access to safe, timely, and affordable cardiac surgery		Advocate for increased global attention and funding to improve access to pediatric cardiac surgery in LMICs, aligned with Sustainable Development Goals. (e.g., World Health Assembly Resolution 71.14 on rheumatic fever and rheumatic heart disease (WHA 71.14)

Abbreviations: APFP, African Pediatric Fellowship Program; LMIC, low- to middle-income country; PCHD, Pediatric and Congenital Heart Disease.

## Discussion

The provision of PCHD services in Africa is critically hampered by severe shortages of specialized personnel, inadequate institutional capacity, significant infrastructure deficiencies, and pronounced geographic disparities, with only 18 countries offering comprehensive PCHD services and many lacking essential training programs, resulting in inadequate care for African children with heart disease, high unmet needs, and preventable morbidity and mortality among children with heart disease (Table 3).

While 78% or 35 of the 45 respondent countries report having some form of cardiac service, only 28 (62%) of the 45 countries surveyed provide pediatric cardiac surgery and 4 of these countries did not have CPB facilities. Additionally, the number of pediatric cardiologists and cardiothoracic surgeons is below international population-based recommendations. Only Libya and Mauritius have the recommended two pediatric cardiologists per million population, and no country has the recommended 1.25 cardiothoracic surgeons per million population.

Less than one-half (46/96, 48%) of respondent institutions provided CPB cardiac surgery, with a median of 0.04 (IQR: 0.03-0.15) centers offering CPB cardiac surgery per million population and with critical deficiencies in Central Africa (Table 1). This ratio is similar to previous estimates for low-income countries of 0 (IQR: 0-0.06) centers per million population and well below that of upper-middle (0.52, IQR: 0-1.02) and high-income (0.75, IQR: 0-1.44) countries.^16^ When ranked according to a composite score based on the recommendations for LMICs PCHD services by Hasan et al,^4^ no institution met all level 5—national-level PCHD referral center criteria and only 8 of 87 (9.2%) respondent institutions met all available criteria for level 4—regional PCHD referral centers (Table 2).

Stratification by AU subregion highlights geographic disparities in PCHD services, with Central and Western Africa showing the greatest need for development (Figure 6). In Southern Africa (black), there is no specific category that is lacking and a generalized expansion of PCHD resources is warranted. Northern (red) and Eastern Africa (blue) are similar but with a lower proportion of centers meeting level 4 or 5 criteria, particularly for PCHD services in Northern Africa, largely related to gaps in ACHD services (Figure 7C).

In contrast, Western (yellow) and Central Africa (green) show generalized deficiencies, with significant gaps in PCHD services and infrastructure, especially for Central Africa (Figure 6). In Western Africa, this is primarily due to deficiencies in human resources with a disproportionate lack of pediatric cardiologists (Figure 7A), lack of infrastructure for interventional services, especially cardiac catheterization (Figure 7C), and related deficiencies in the provision of catheterization services.

The situation in Central Africa is similar but worse with a critical lack of level 4 or 5 cardiothoracic surgery, anesthesiology, and perfusionist human resources. When combined with the severe lack of surgical infrastructure it is unsurprising that the provision of cardiothoracic surgery services is lacking (Figure 7B). Despite similar proportions of centers with level 4 or 5 cardiology human resources to that seen in Eastern Africa, in Central Africa there are no centers with level 4 or 5 catheterization infrastructure, which severely hampers the provision of interventional catheterization services.

These data are especially concerning given the significant shortage of pediatric cardiology and cardiothoracic surgery fellowship programs. Only 13/45 (29%) countries report having both pediatric cardiology and cardiothoracic surgery fellowship training programs (Figure 5) with the greatest deficits in Western and Central Africa (Figure 7). This shortage must be addressed if PCHD capacity is to be increased. One promising model for addressing this gap is the African Pediatric Fellowship Program (APFP) at the University of Cape Town.^17^ The APFP provides specialized training to doctors from across Africa, including in pediatric cardiology and cardiothoracic surgery. Through its “train the trainer” model, the APFP equips African specialists with the skills needed to return to their home countries and train the next generation of health professionals ensuring sustainable growth in the availability of pediatric services across the continent. It would be highly beneficial for other countries in Africa to develop similar programs, enabling them to build African expertise and reduce reliance on external training, further bolstering the continent's ability to manage pediatric congenital heart disease.

In conclusion, these results emphasize that PCHD services across Africa remain critically insufficient, with significant disparities between regions. Central and Western Africa are particularly underserved, where a lack of vital training programs is likely exacerbating the issue. As progress is made in other health areas, PCHD has become an important focus in the reduction of childhood deaths and the realization of the United Nations', 2016, Sustainable Development Goals. Despite this, progress in global cardiac surgery, particularly in Africa and other low- and middle-income regions, remains slow.

## Limitations

This study is subject to the limitations and biases inherent in surveys. Despite efforts to include a broad range of professionals and a systematic approach to identifying respondents, the sample may not fully represent the entire spectrum of PCHD care providers across Africa, and certain groups may be misrepresented due to the way participants were selected or volunteered to participate. The survey relies on self-reported data from respondents, which is subject to response and recall bias. When ranking institutions according to the Hasan et al guideline, not all criteria used in the guideline were available. As such, the analysis represents a best-case scenario given the available data. For instance, the guideline specifies the equipment and supplies required at each institutional level. Since these data are unavailable, a center that meets level 4 criteria in all categories except equipment and supplies would still be categorized as a level 4 center in this analysis. The same applies to specific unavailable variables within the categories described above. For example, the guideline states that level 4 and 5 centers should provide an electrophysiology service. As these data were not recorded, no center would be downgraded due to a lack of electrophysiology services.

## Supplemental Material

sj-docx-1-pch-10.1177_21501351251316230 - Supplemental material for A Landscape Analysis of Pediatric and Congenital Heart Disease Services in Africa Supplemental material, sj-docx-1-pch-10.1177_21501351251316230 for A Landscape Analysis of Pediatric and Congenital Heart Disease Services in Africa by Thomas Aldersley, Sulafa Ali, Adila Dawood, Frank Edwin, Kathy Jenkins, Alexia Joachim, John Lawrenson, Darshan Reddy, Drissi Boumzebra, James D. St. Louis, Christo Tchervenkov, Amy Verstappen, Bistra Zheleva, Liesl Zühlke and in World Journal for Pediatric and Congenital Heart Surgery

## Abbreviations

ACHD: adult congenital heart disease
CHD: congenital heart disease
CPB: cardiopulmonary bypass
IQR: interquartile range
LMIC: low- to middle-income country
PCHD: Pediatric and Congenital Heart Disease
RHD: rheumatic heart disease
