# Supplementary material for: A Landscape Analysis of Pediatric and Congenital Heart Disease Services in Africa
Source: World J Pediatr Congenit Heart Surg. 2025 Aug 21;16(6):827–38. doi: 10.1177/21501351251316230 (PMC12504779; doi:10.1177/21501351251316230)

A landscape analysis of paediatric and congenital heart disease services in Africa

Supplemental Figures and Tables


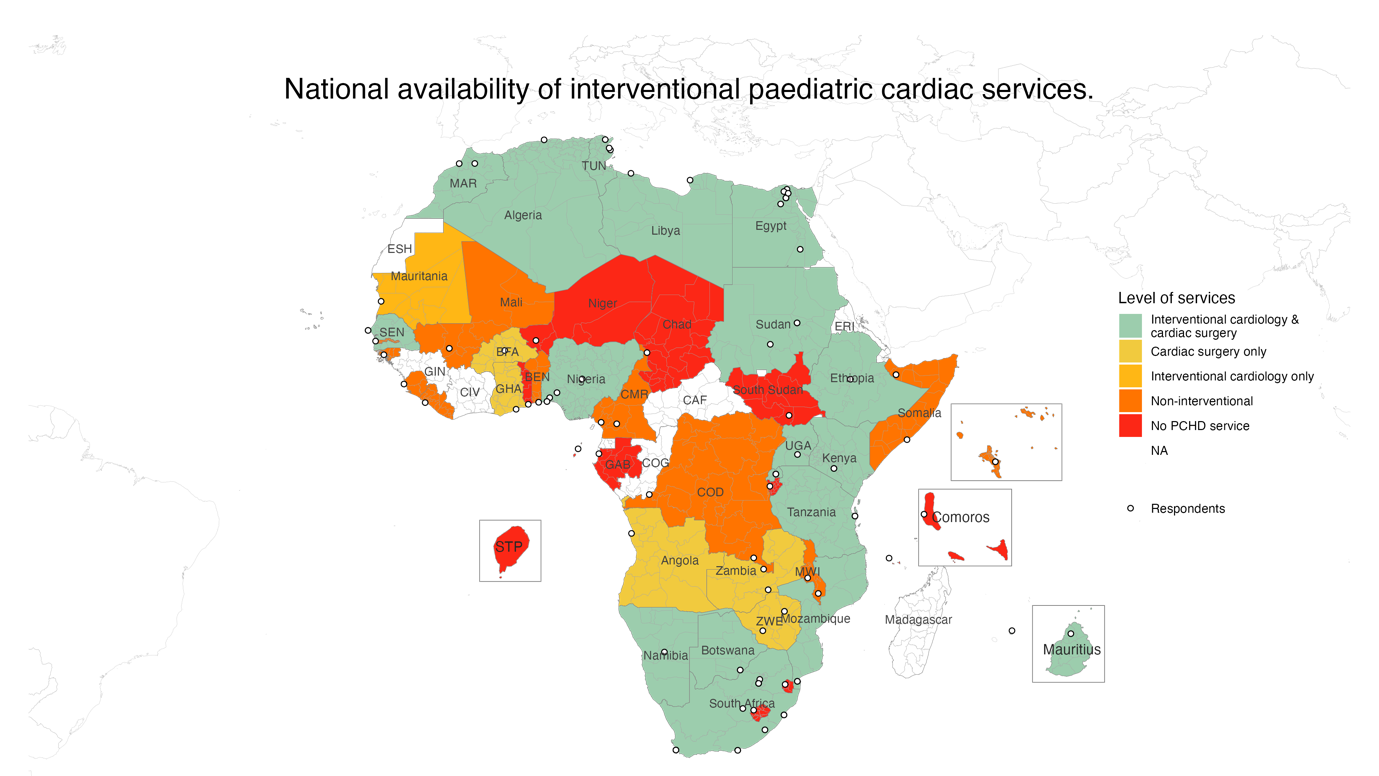


Figure S1: Choropleth depicting availability of cardiac services at the national level. These data exclude cardiac centers with no cardiopulmonary bypass service.

BEN: Benin, BFA: Burkina Faso, CAF: Central African Republic, CIV: Ivory Coast, CMR: Cameroon, COD: Democratic Republic of the Congo, COG: Republic of the Congo, ERI: Eritrea, ESH: Sahrawi Arab Democratic Republic, GAB: Gabon, GHA: Ghana, GIN: Guinea, MAR: Morocco, MWI: Malawi, SEN: Senegal, TUN: Tunisia, UGA: Uganda, ZWE: Zimbabwe.


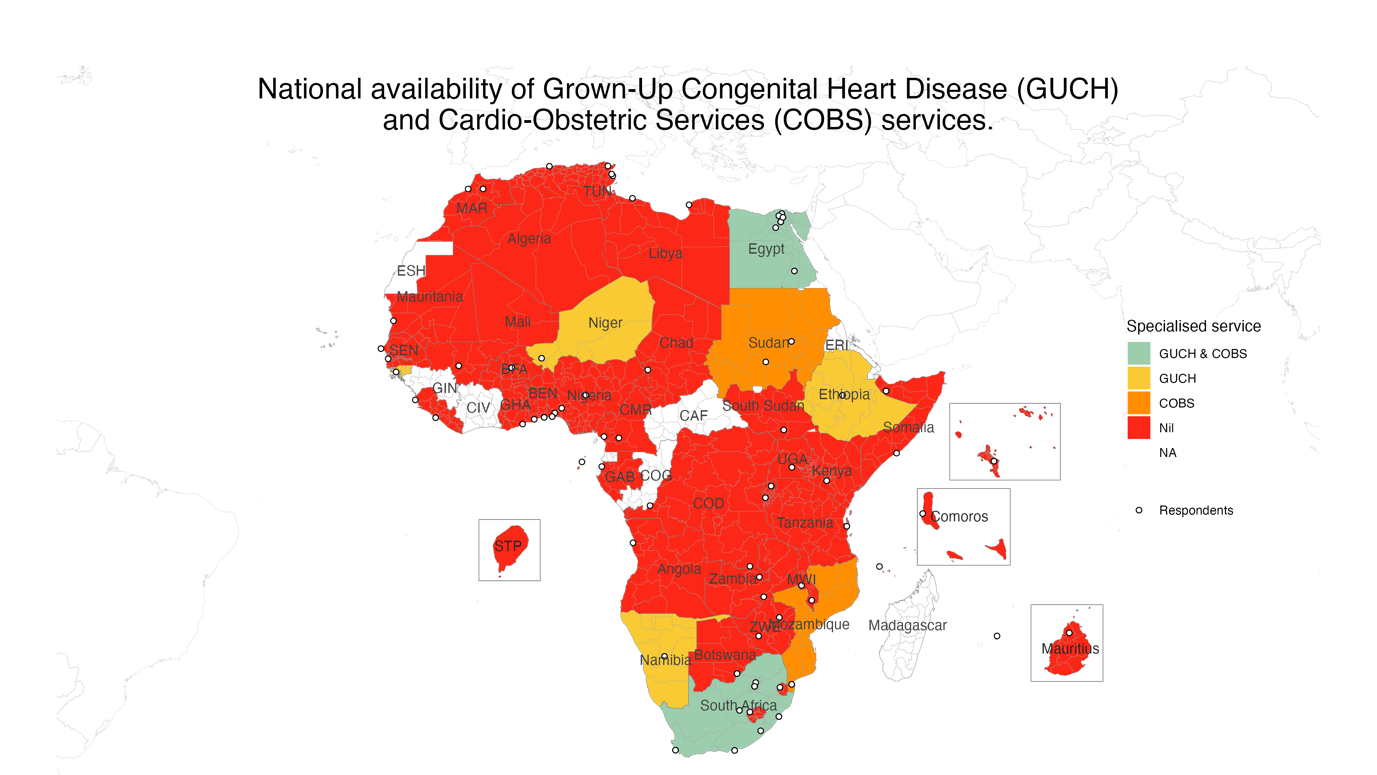


Figure S2: Choropleth depicting the availability of specialised cardiac services including grown-up congenital heart disease (GUCH) and cardio-obstetric (COBS) services at the national level.

BEN: Benin, BFA: Burkina Faso, CAF: Central African Republic, CIV: Ivory Coast, CMR: Cameroon, COD: Democratic Republic of the Congo, COG: Republic of the Congo, ERI: Eritrea, ESH: Sahrawi Arab Democratic Republic, GAB: Gabon, GHA: Ghana, GIN: Guinea, MAR: Morocco, MWI: Malawi, SEN: Senegal, TUN: Tunisia, UGA: Uganda, ZWE: Zimbabwe.

Table S1: Criteria used to rank institutions according to the Hassan et al’s recommendations for developing PCHD services in low and middle-income countries

| Category | Sub-category | Level 4 Criteria | Level 5 Criteria |
| --- | --- | --- | --- |
| Staffing | Cardiology | Paediatric cardiologists (≧1) | Paediatric cardiologists (≧2) |
|  | Surgery | Paediatric congenital cardiac surgeon or adult cardiac surgeons with paediatric experience. (≧1) | Paediatric congenital cardiac surgeons (≧2).  Expertise in neonatal cardiac surgery (NA). |
|  | Anaesthesiology | Anaesthetist with significant experience in paediatric cardiac cases. | Paediatric cardiac anaesthetists |
|  | Allied Health | Congenital cardiac perfusionists. Congenital cardiac sonographers (NA) CICU nurses (NA) | Congenital cardiac perfusionists. Congenital cardiac sonographers (NA) CICU nurses (NA) |
|  | Intensivists | Paediatric intensivists with CICU training | Dedicated congenital cardiac intensivists |
| Infrastructure | Cardiology | Basic inpatient, outpatient, and emergency infrastructure. Functionally and physically separate paediatric echocardiography lab. | Full spectrum of inpatient, outpatient, and emergency infrastructure. Paediatric electrophysiology expertise (NA). |
|  | Cardiac catheterisation | Infrastructure for biplane cardiac catheterization, may be shared with adult cardiac services. | Functionally separate paediatric biplane cardiac catheterization services. |
|  | Surgical | Infrastructure for cardiac surgery. | Infrastructure for cardiac surgery. |
|  | ICU | Infrastructure for CICU. | Functionally and physically separate infrastructure for CICU. ECMO preferable (NA). |
| PCHD Services | Catheterisation service | Emergent & elective cardiac catheterization. PREDIC3T categories 0-4 (NA). | All procedures. |
|  | Diagnostic cardiology service | Echocardiography, Electrocardiogram (NA), X-ray, Holter Monitoring (NA), Diagnostic Cardiac Catheterization, CT (can be shared with adult). | Echocardiography, Electrocardiogram (NA), X-ray, Holter Monitoring, Cardiac Catheterization, cardiac CT scanner (minimum 64 slice) and cardiac MRI (preferable). |
|  | Cardiothoracic surgery service | Cardiothoracic surgery service. RACHS 1 & 2 procedures. (NA proxy variable = bypass) | Cardiothoracic surgery service. All procedures. |
|  | ACHD services | Adult CHD care | Specialised ACHD service. |
|  | ICU services | Congenital cardiac intensive care (preferable). | Dedicated CICU |
|  | Other services | Hospital infection control programme and blood bank. | Hospital infection control programme, blood bank, and transplantation programme. |
| Training & Research | Training | Cardiology or cardiothoracic surgery fellowship programme | Cardiology and cardiothoracic surgery fellowship programme |
|  | Research (NA) | Integrated research programmes (NA). | Integrated research programmes with postgraduate research outputs (NA). |
| Health data & Quality Control |  | A data registry for diagnostic imaging, cardiac catheterisation, and surgery - consistently track and report their outcomes. QI framework, QI & safety representative (certification, CME, CPD) (NA). | A data registry for diagnostic imaging, cardiac catheterisation, and surgery - consistently track and report their outcomes. QI framework, QI & safety representative (certification, CME, CPD) (NA). |
| NA: Data not available | | | |

Appendix 1: Survey document - A landscape analysis of paediatric and congenital heart disease services in Africa
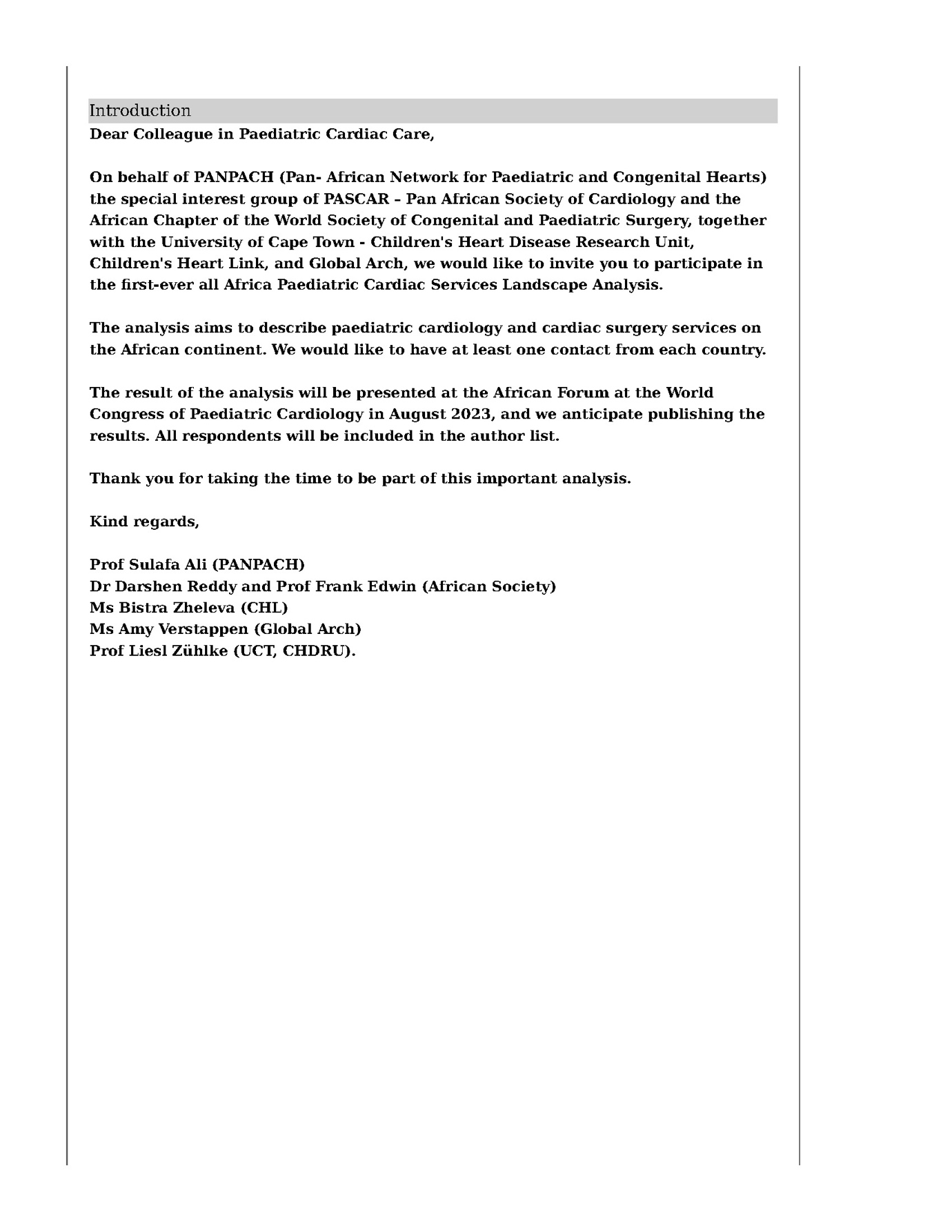


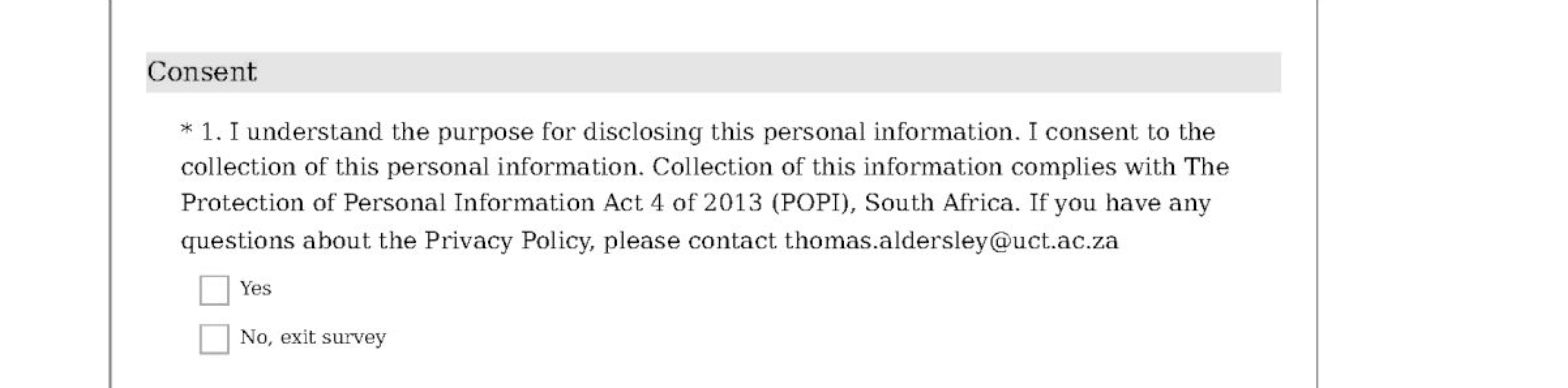


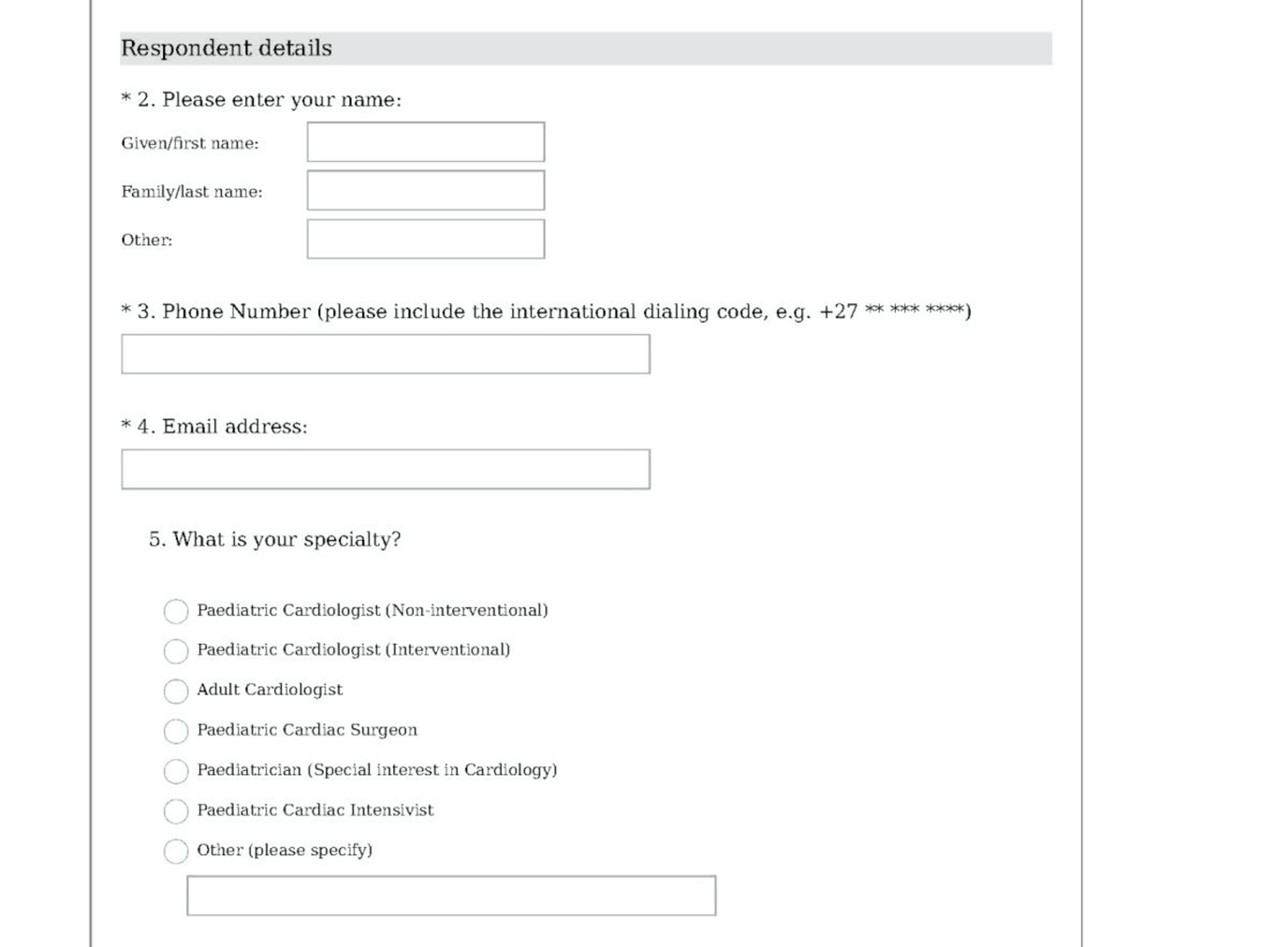


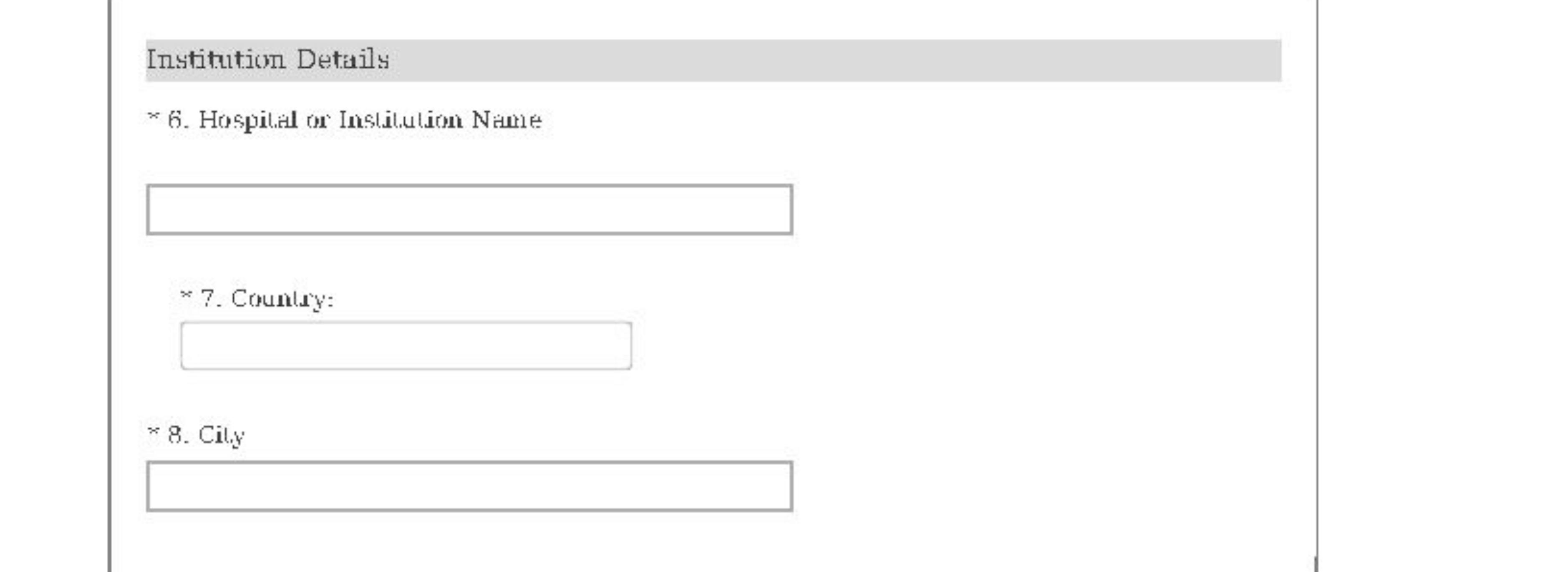


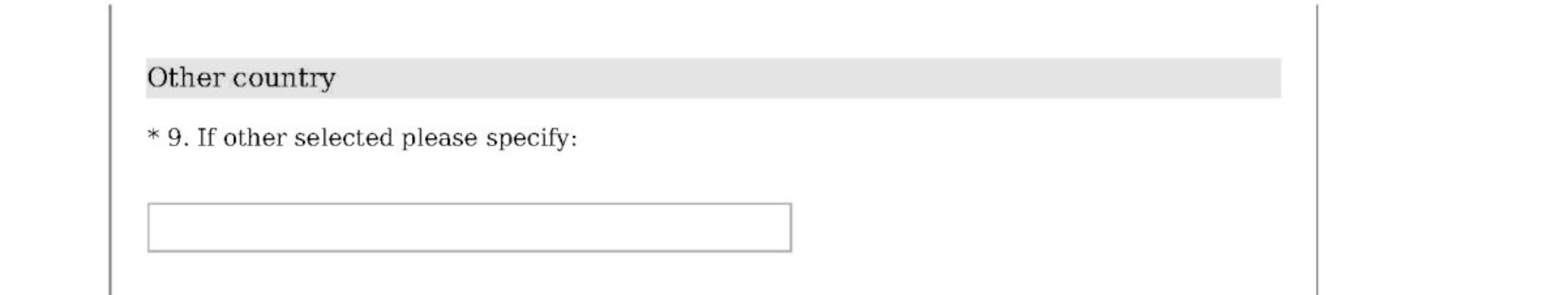


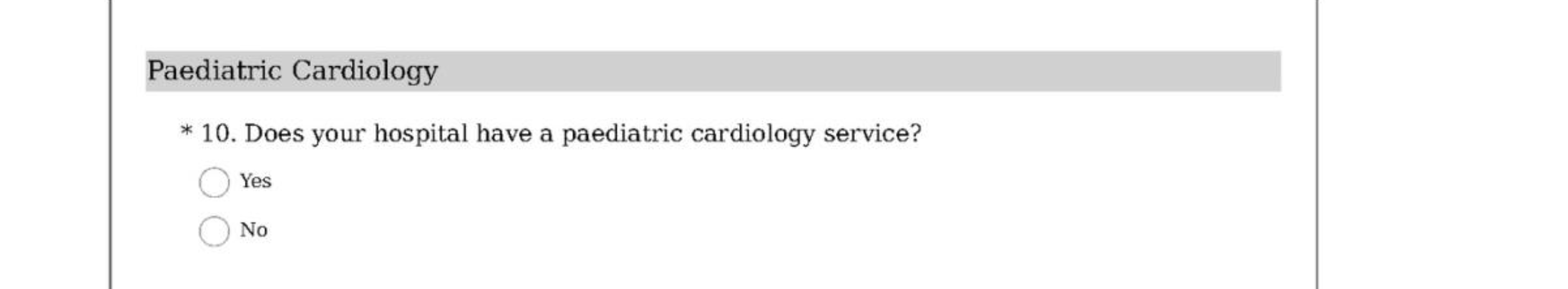


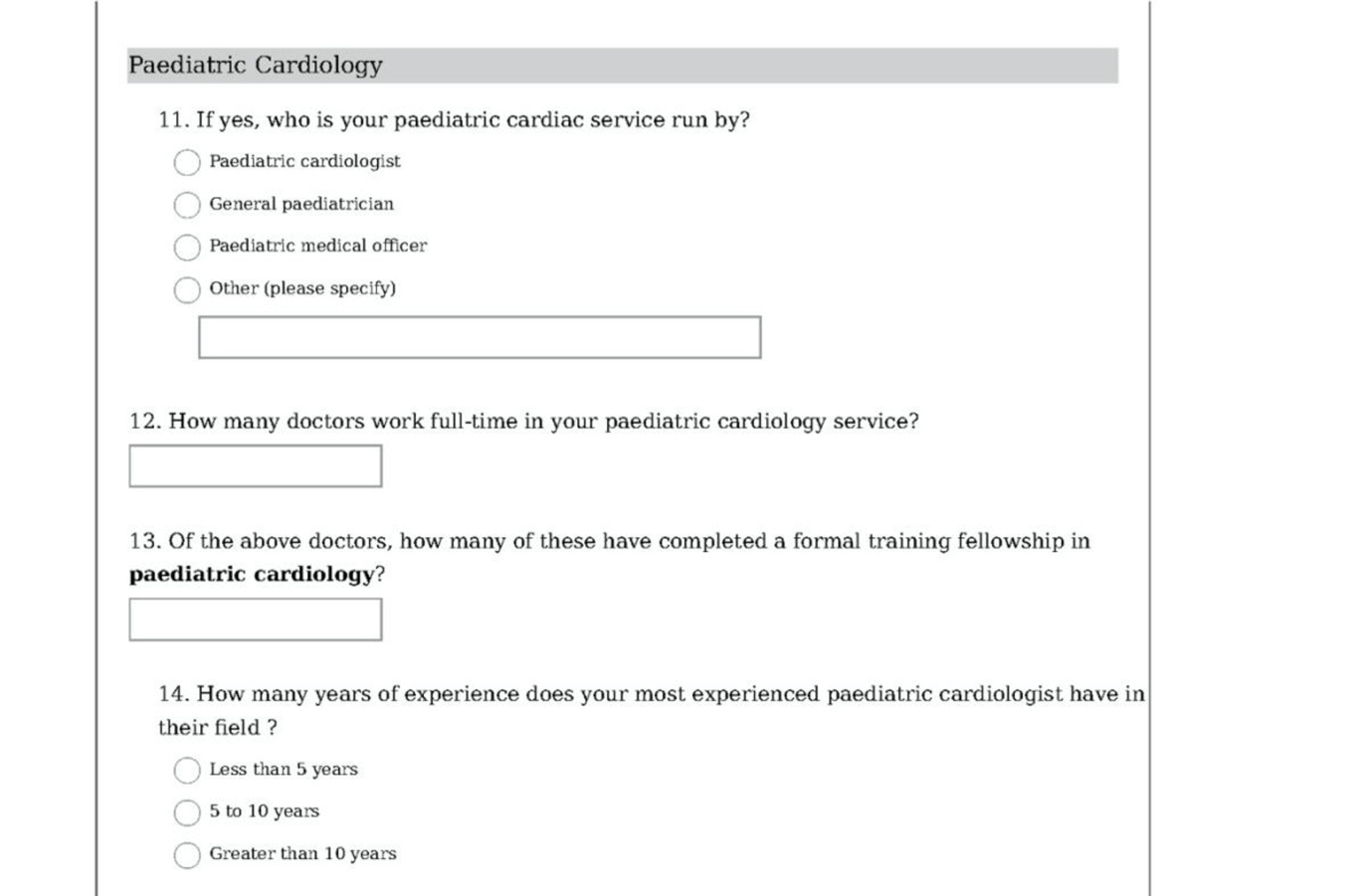


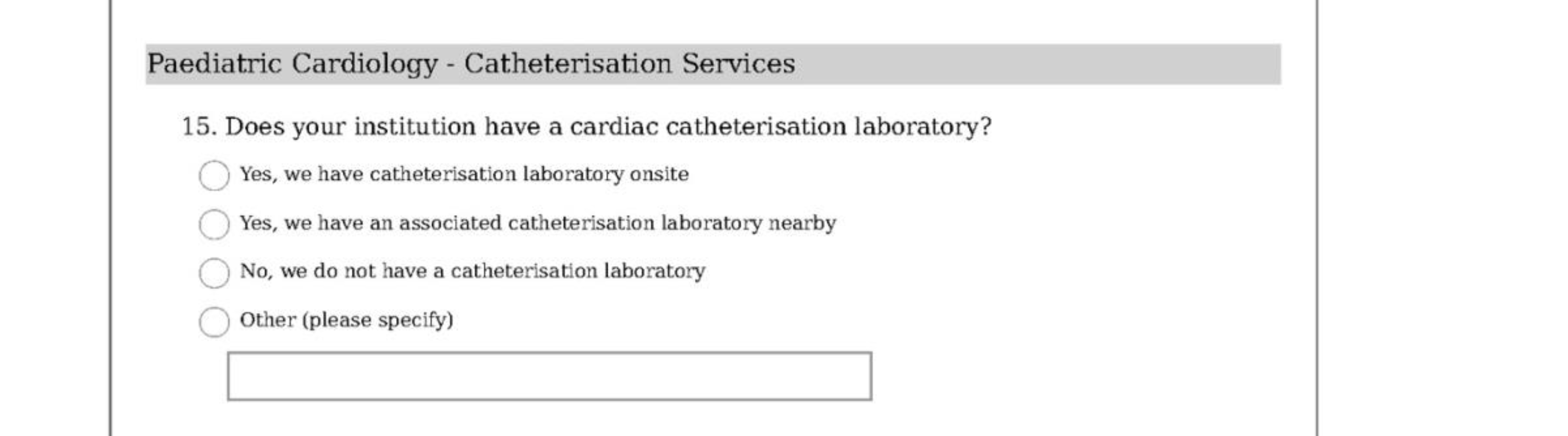


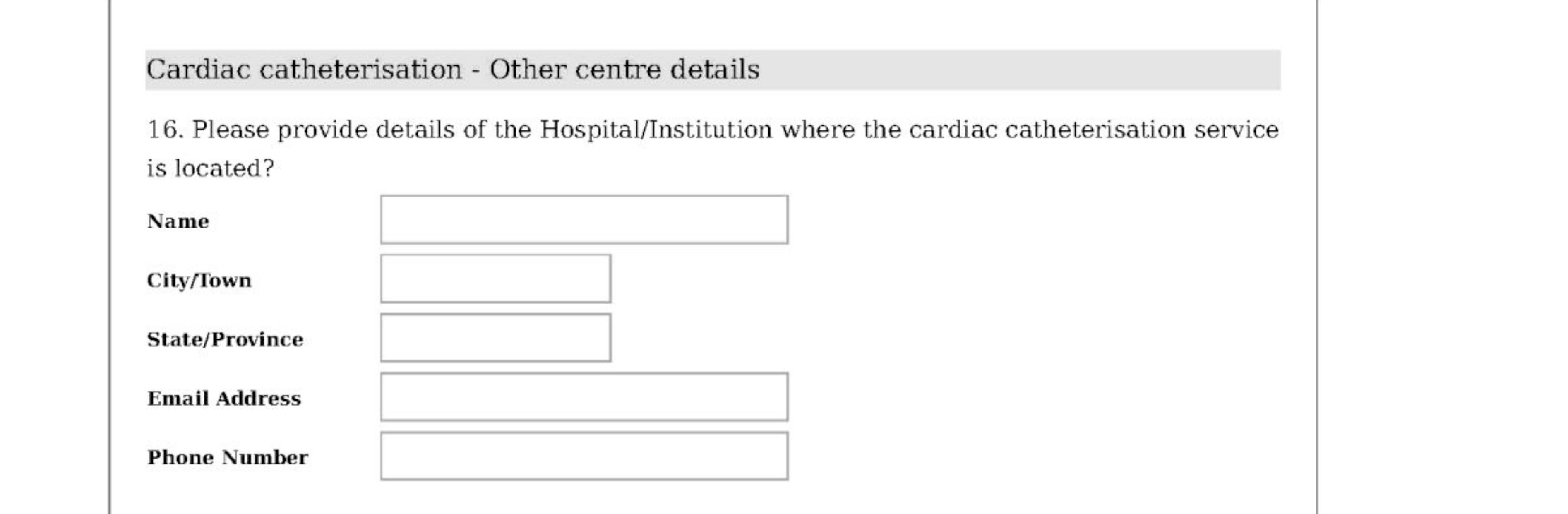


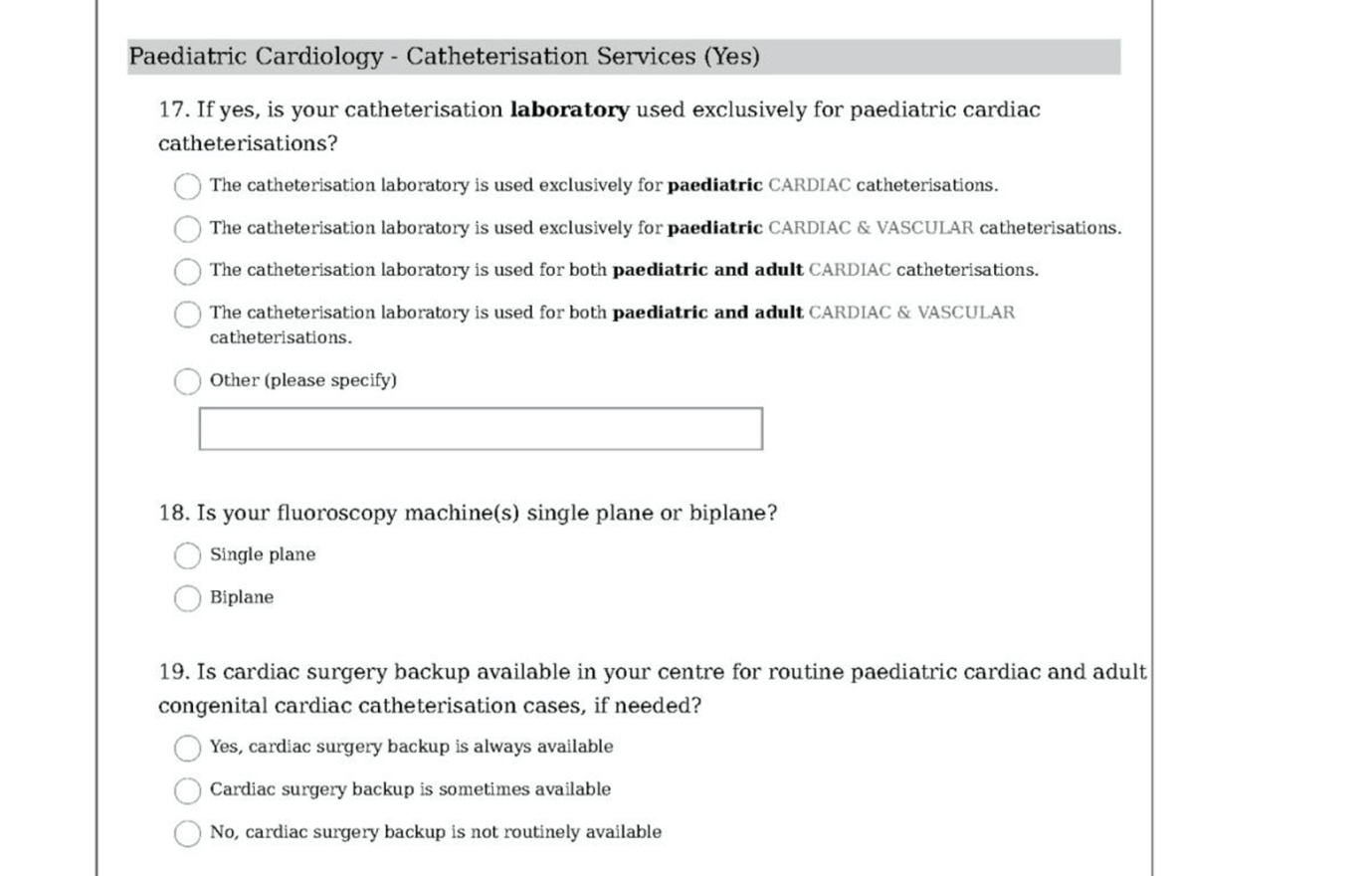


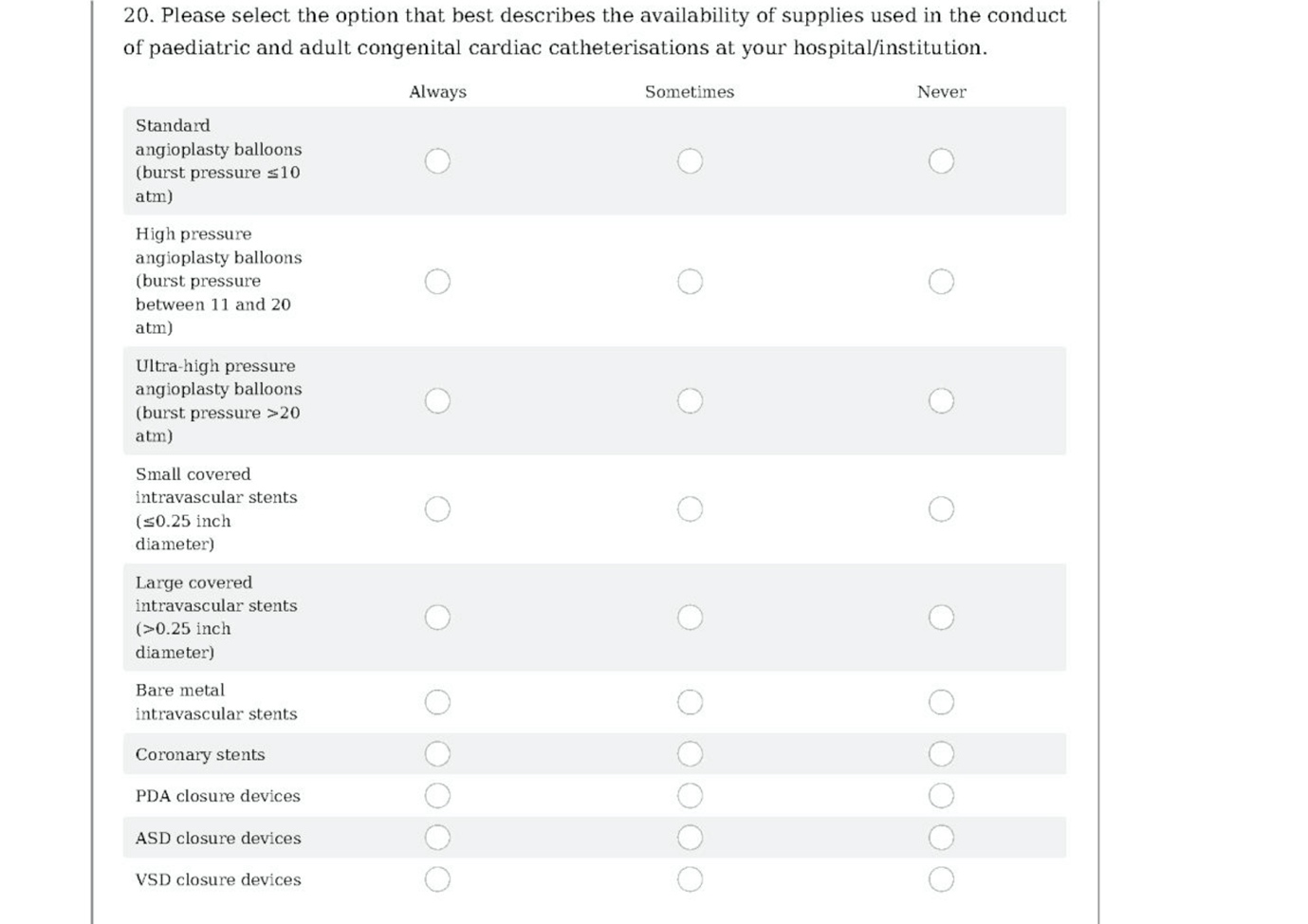


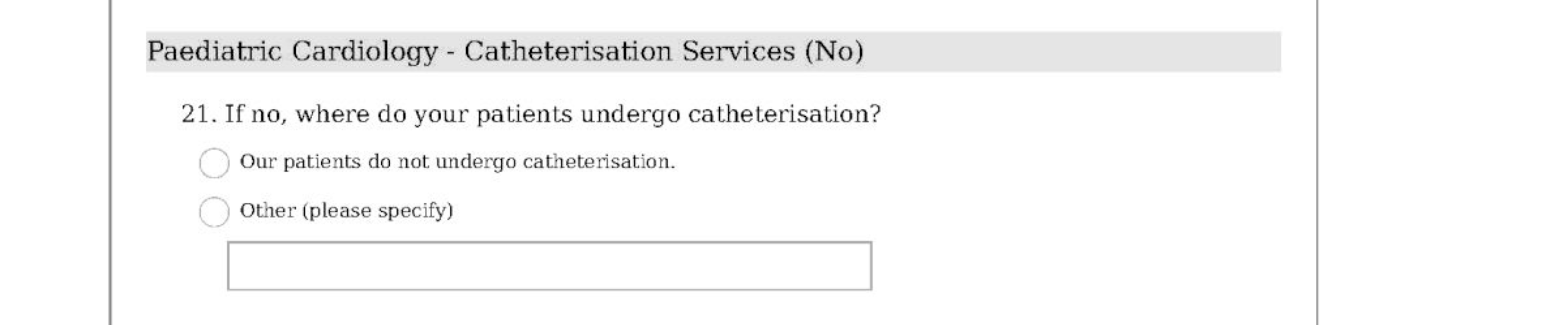


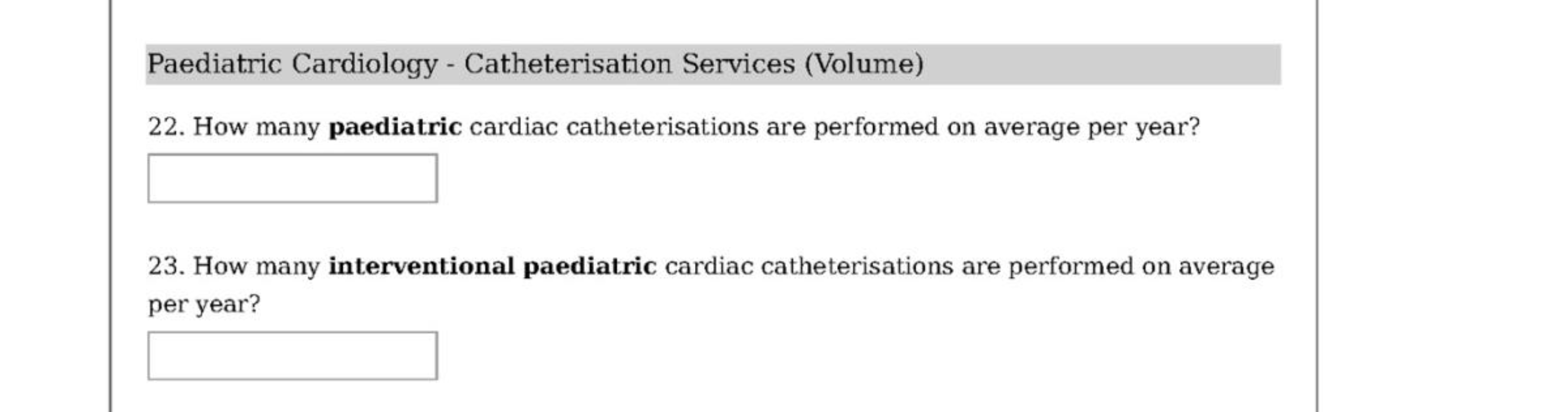


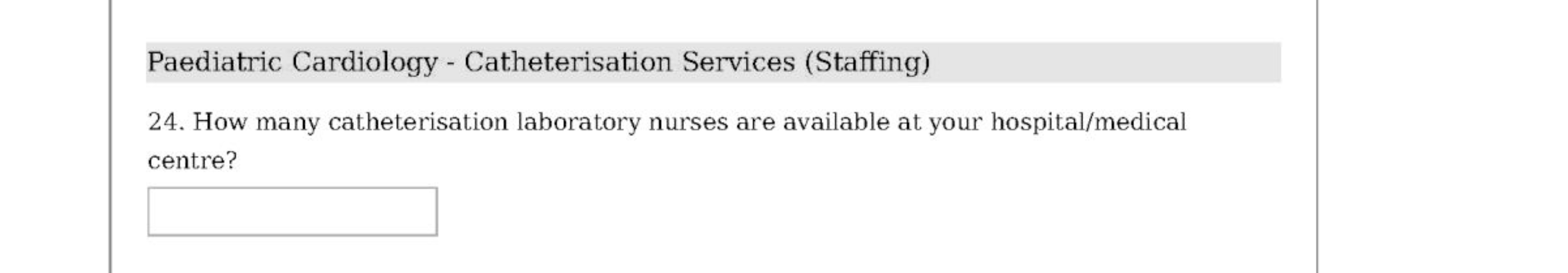


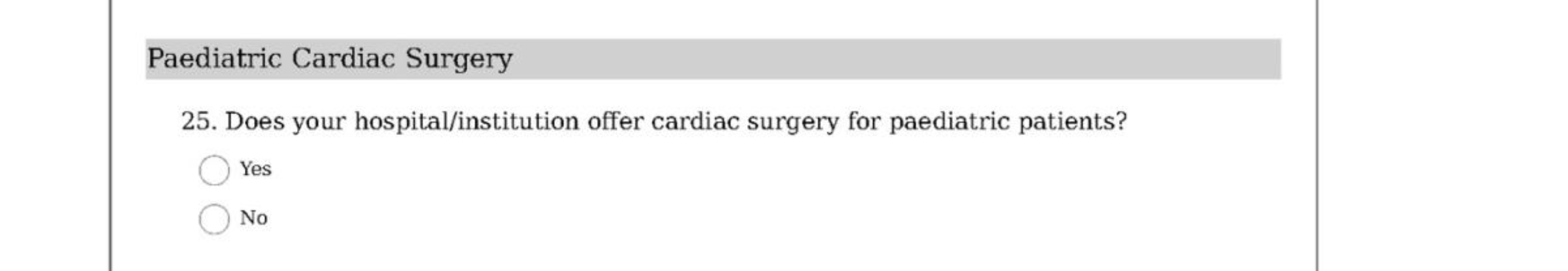


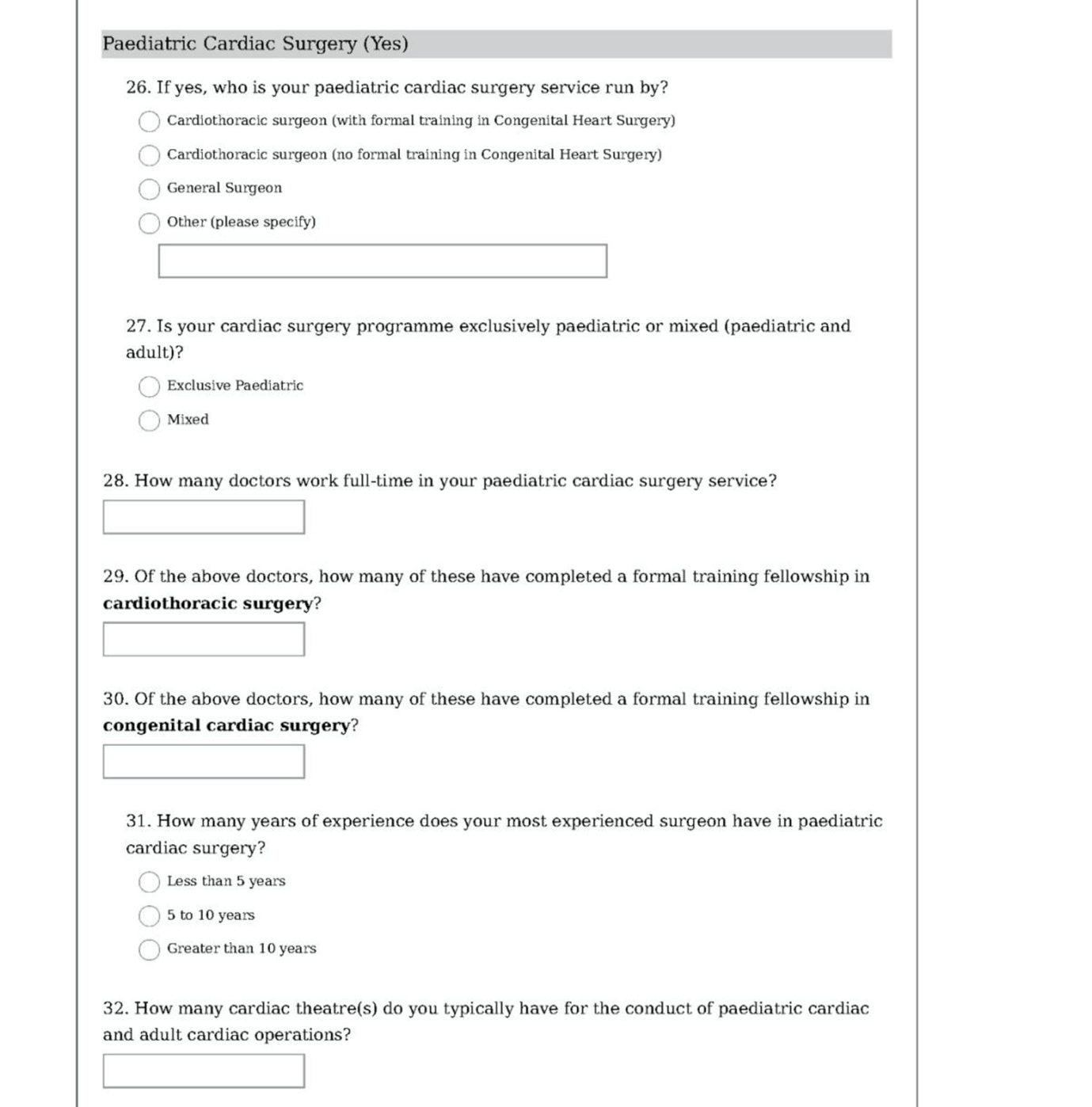


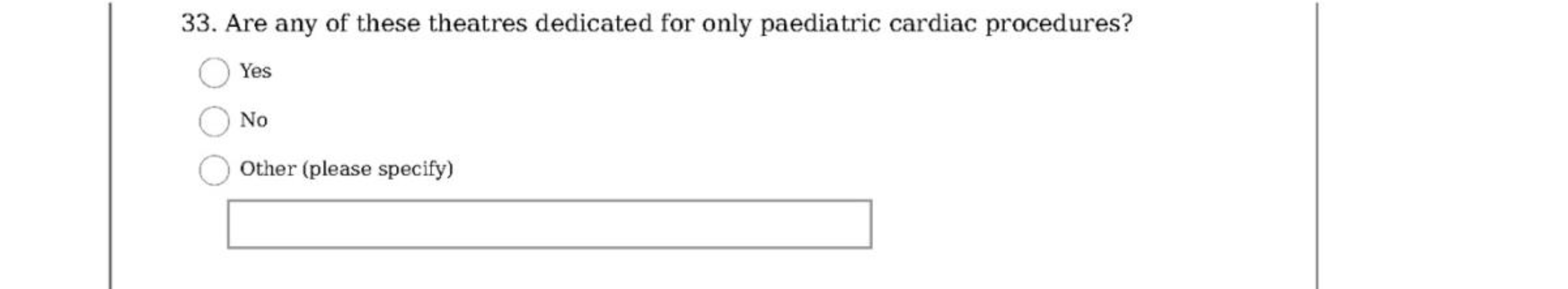


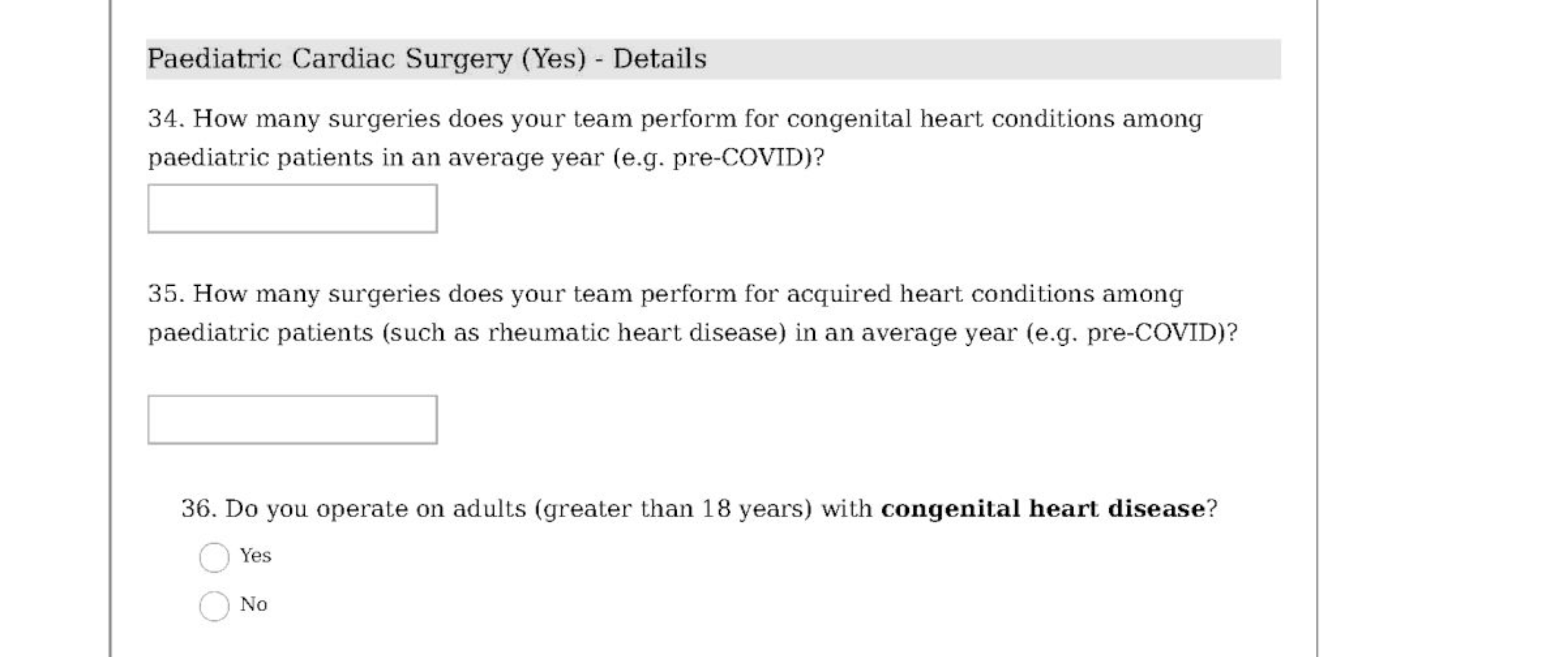


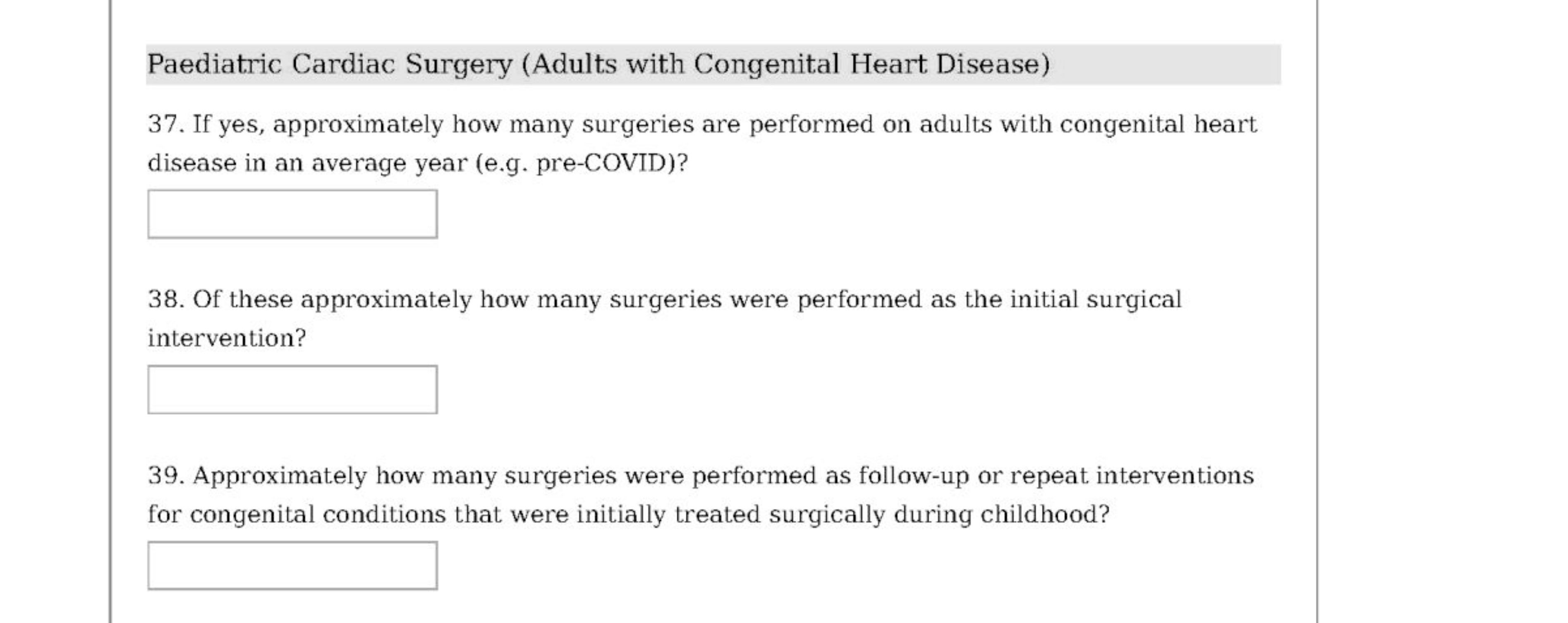


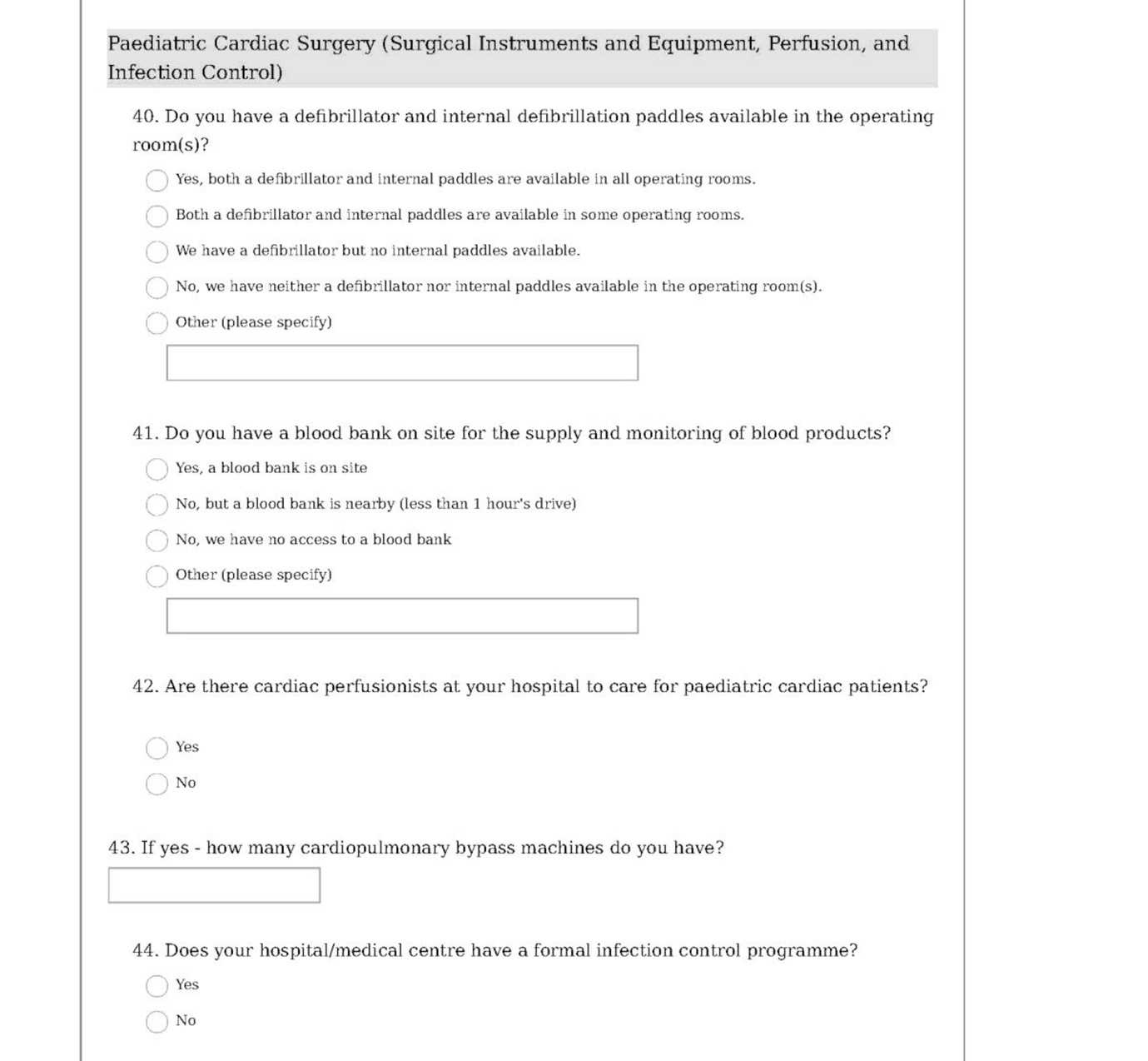


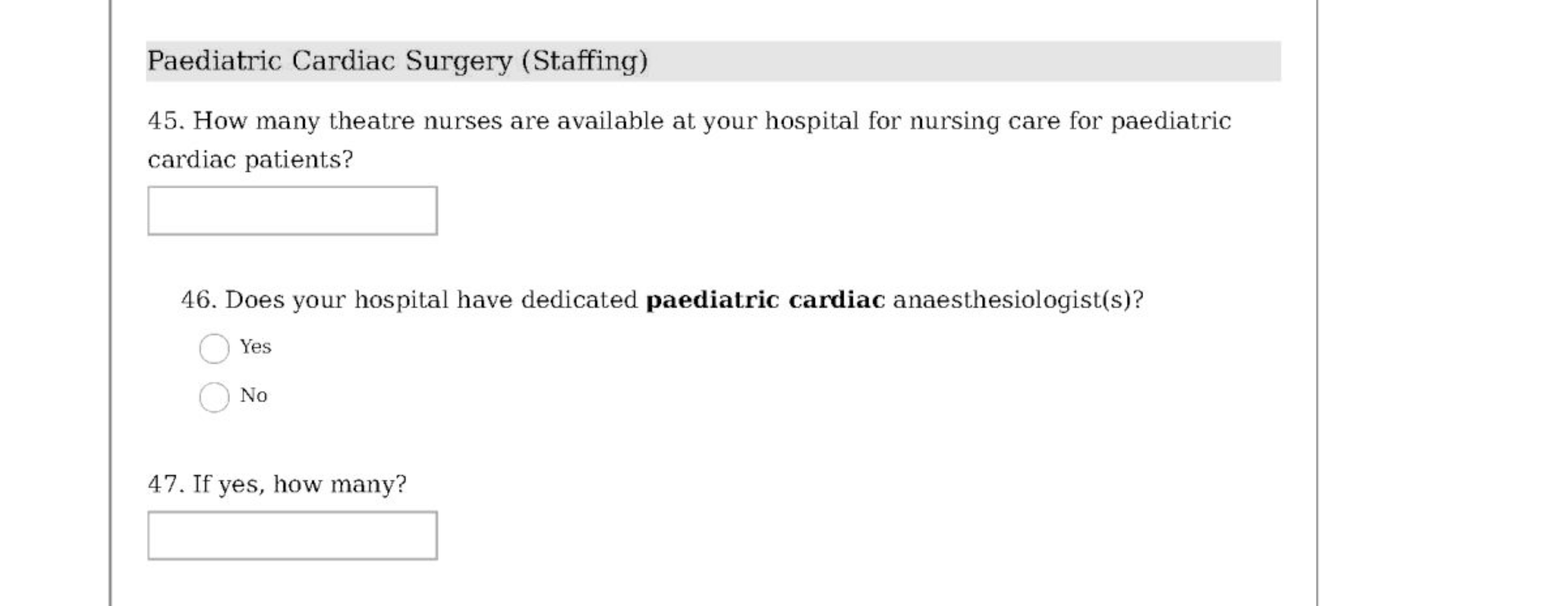


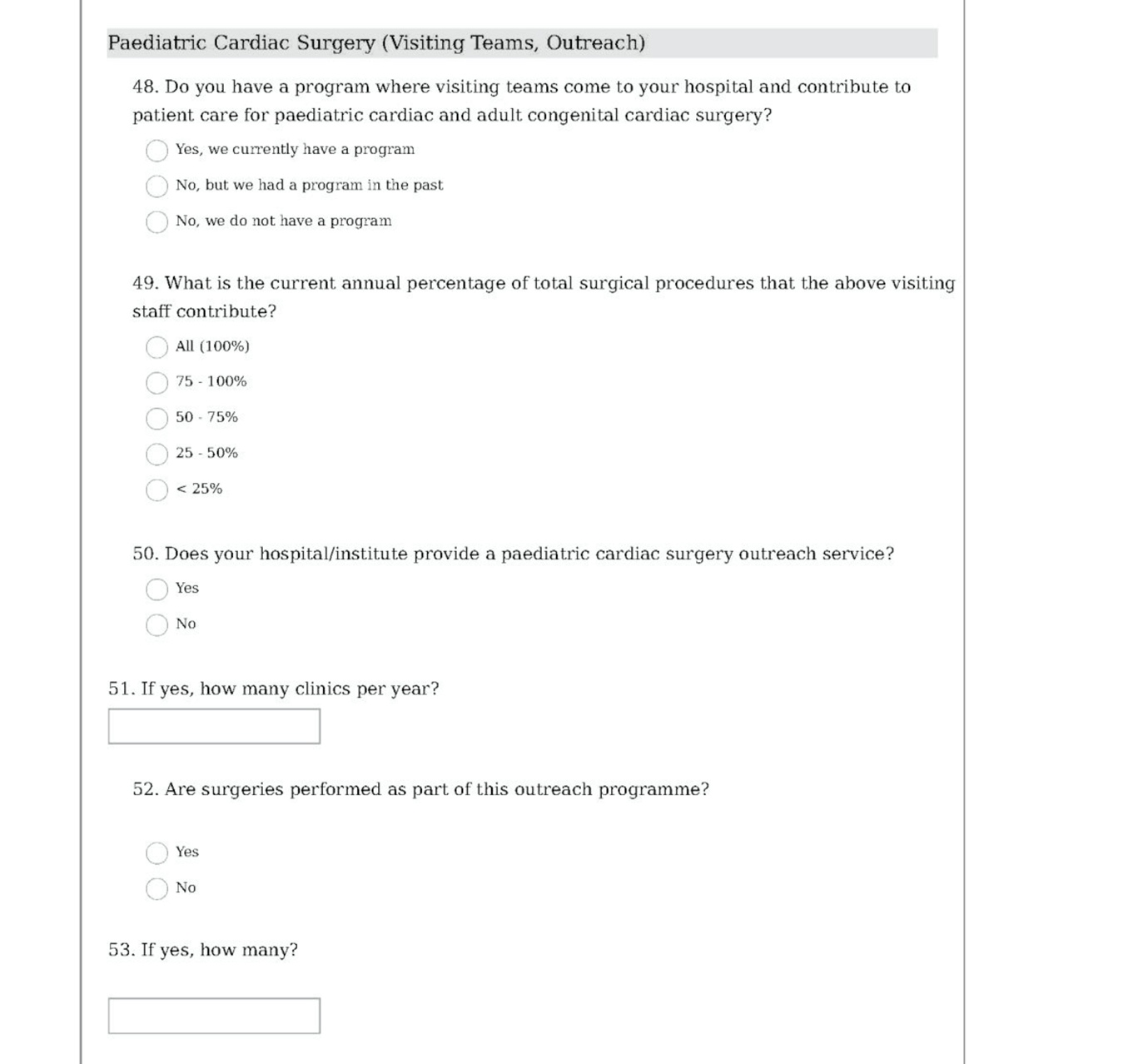


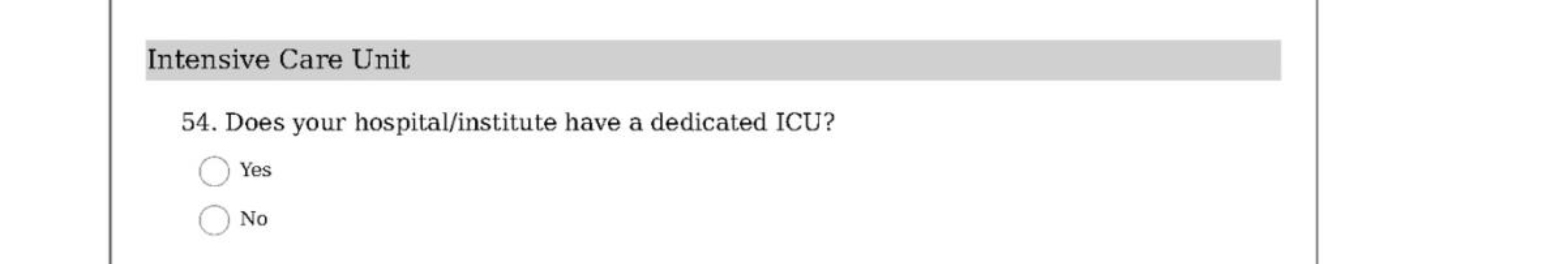


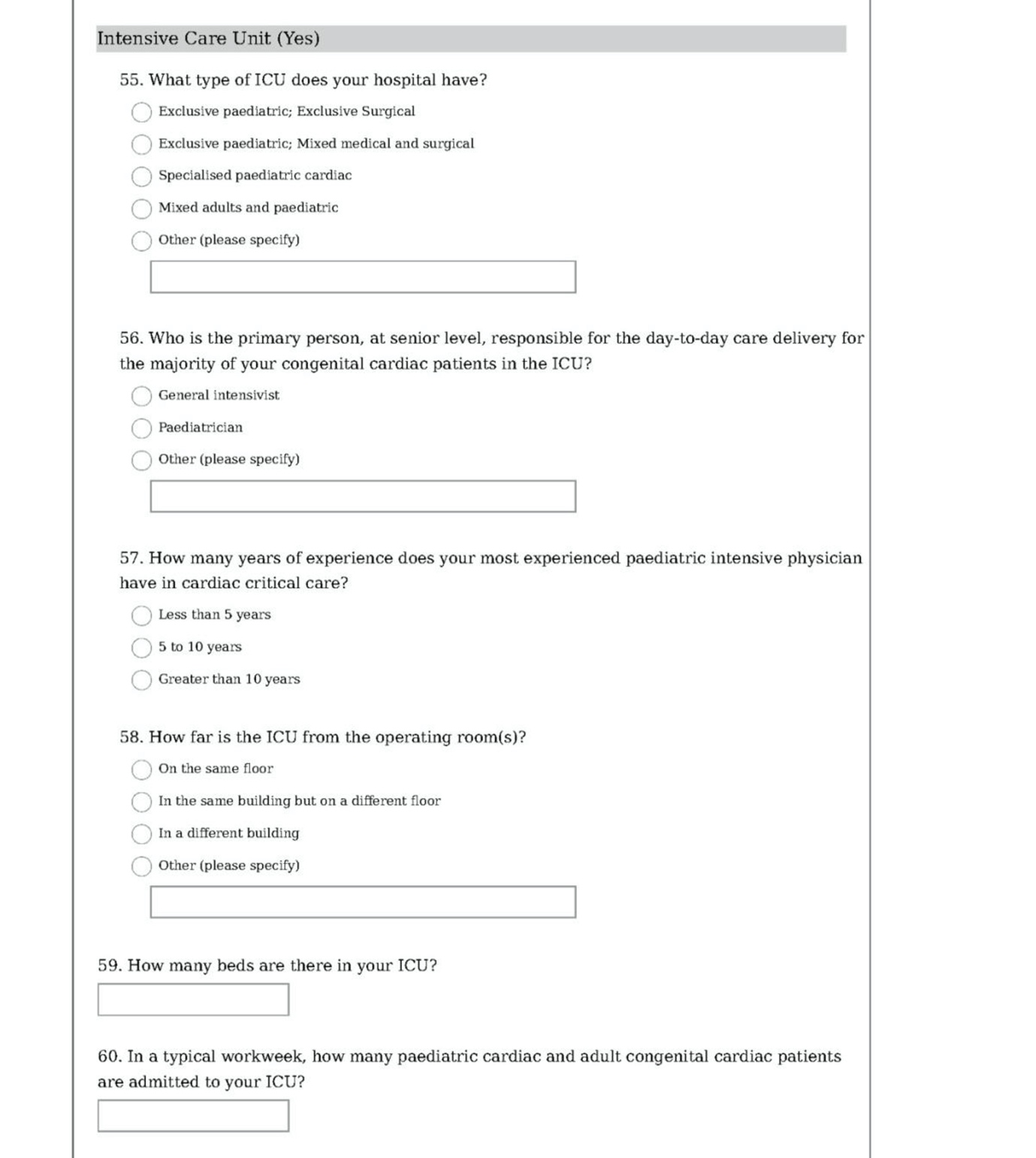


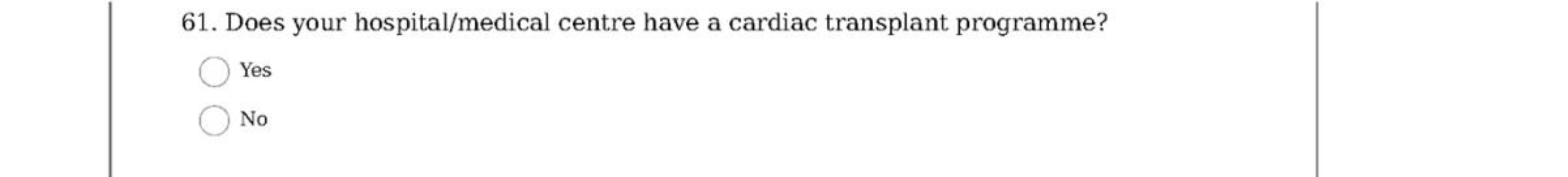


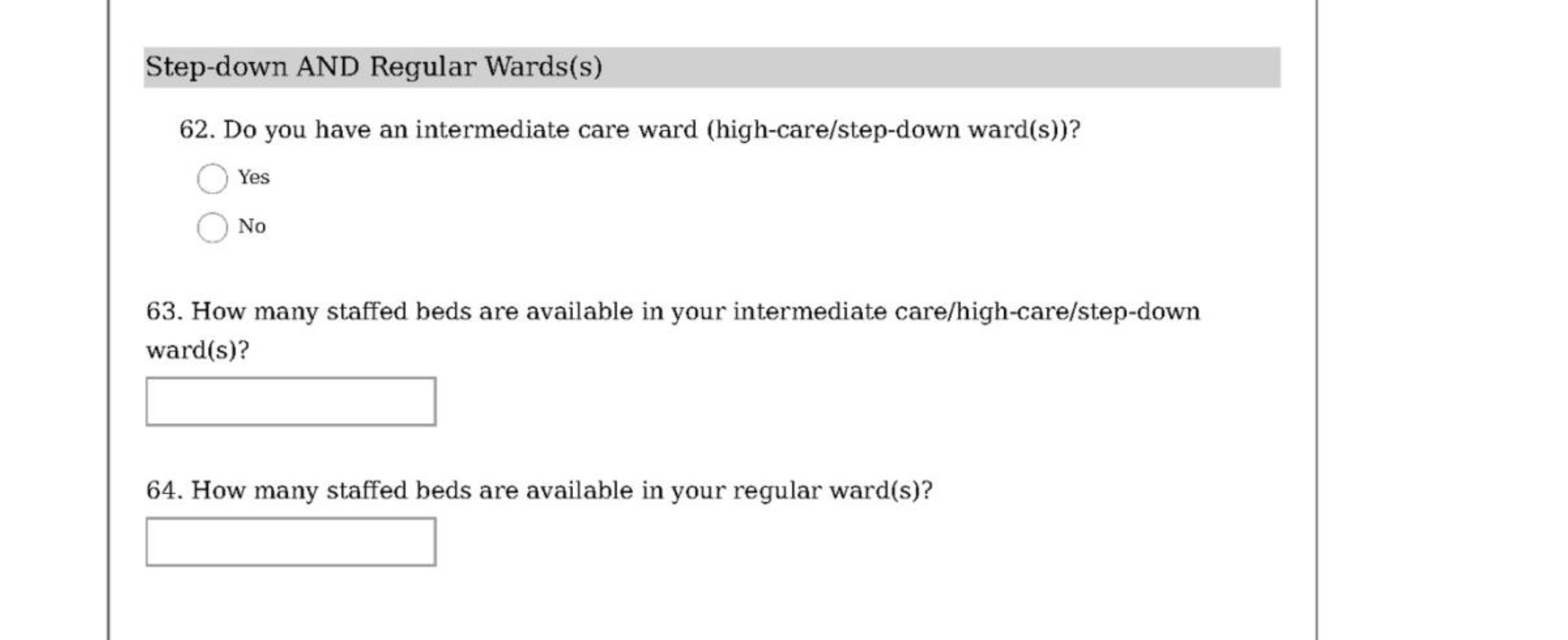


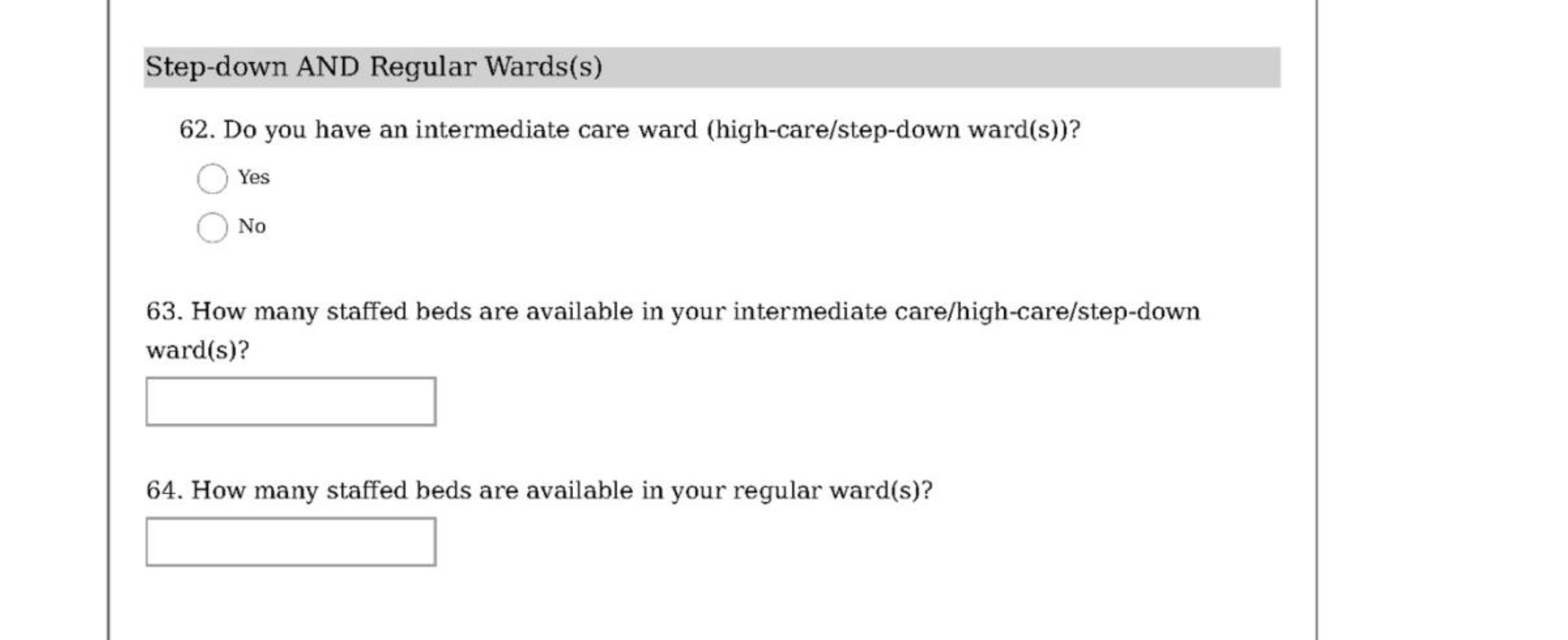


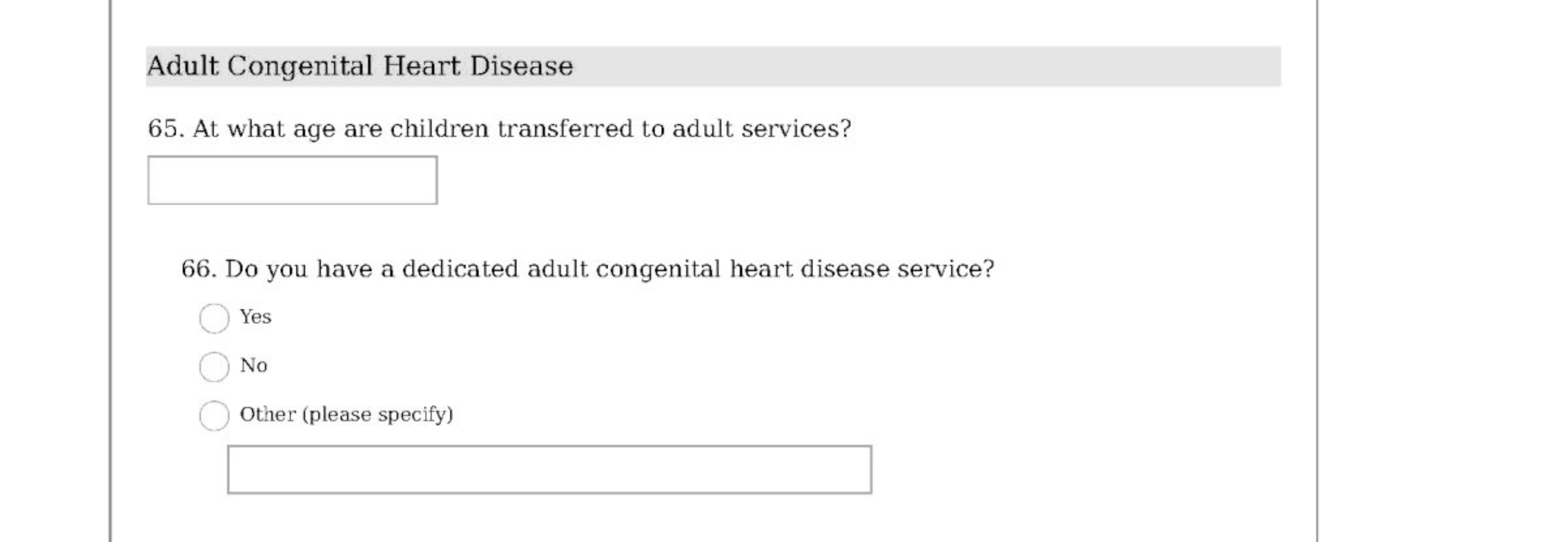


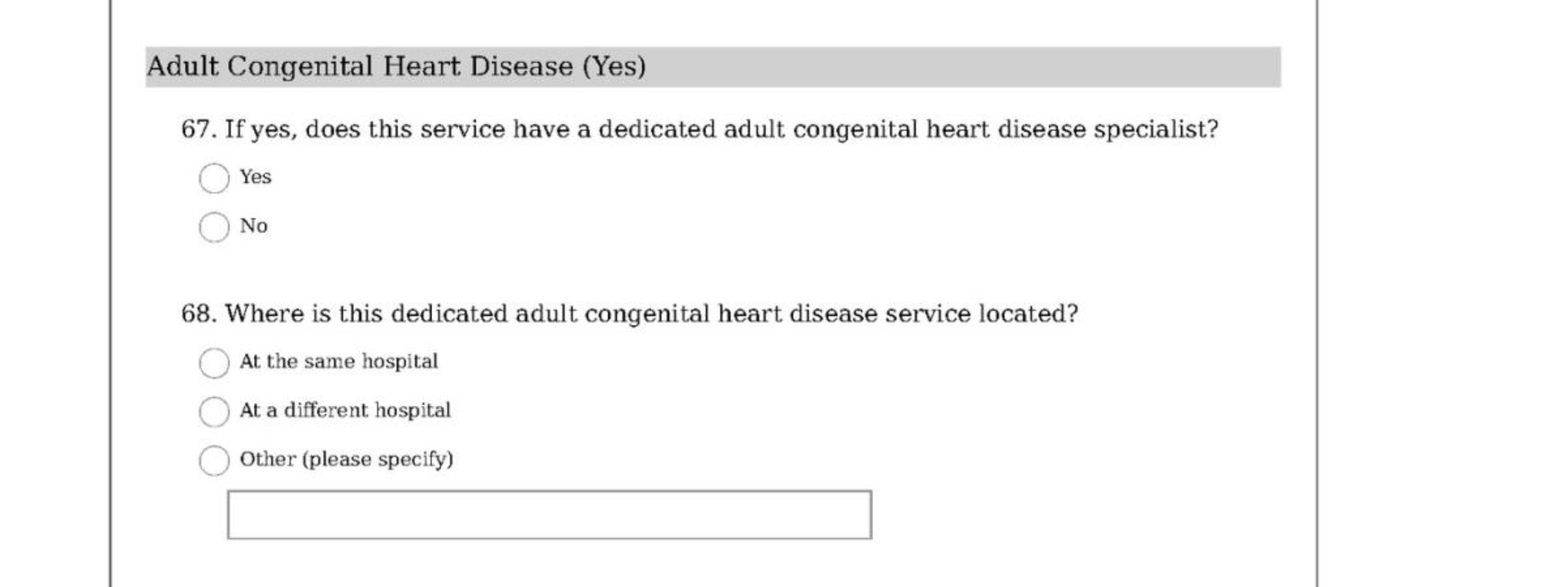


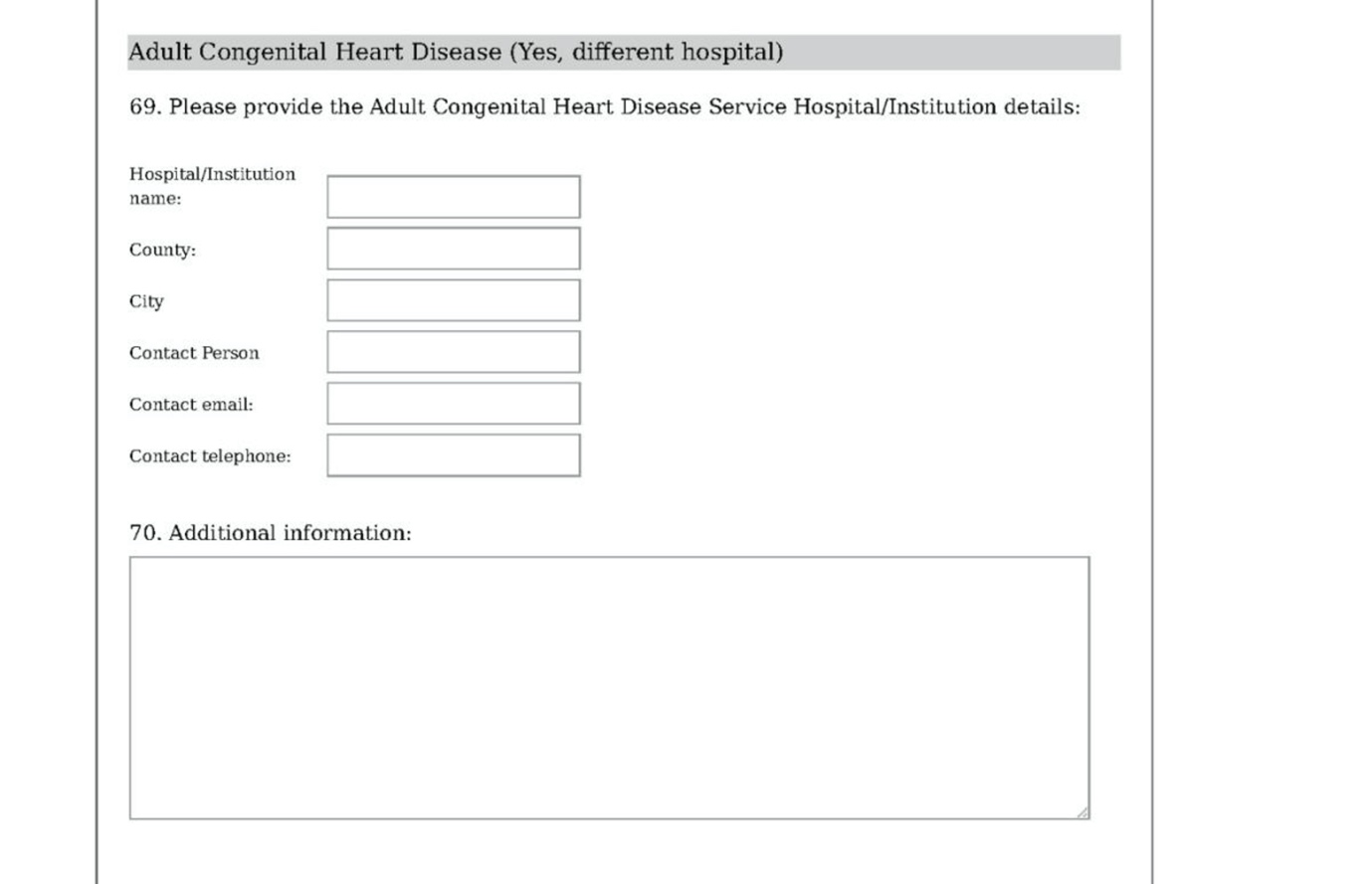


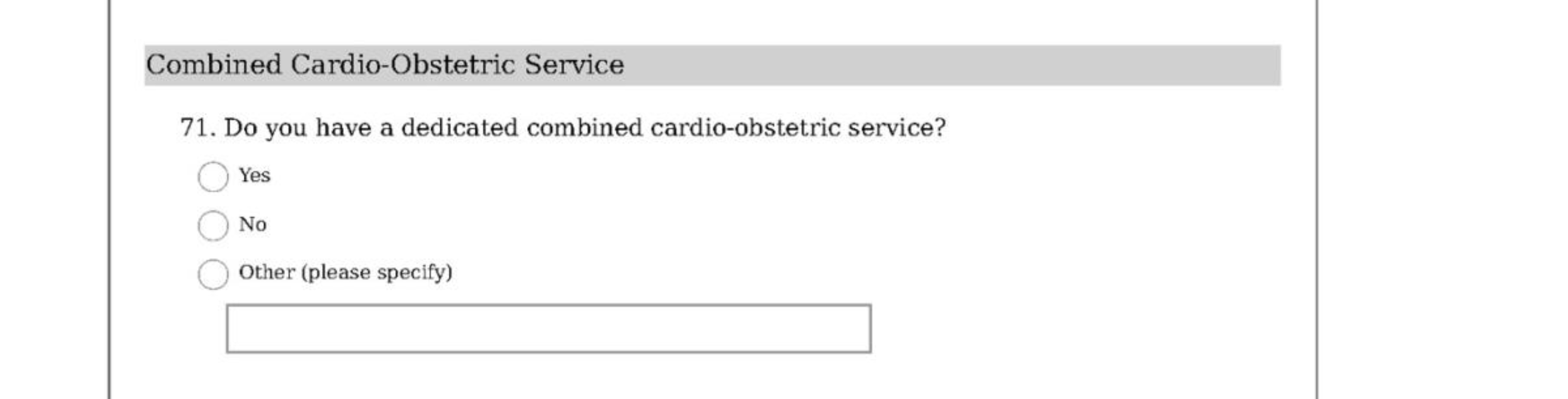


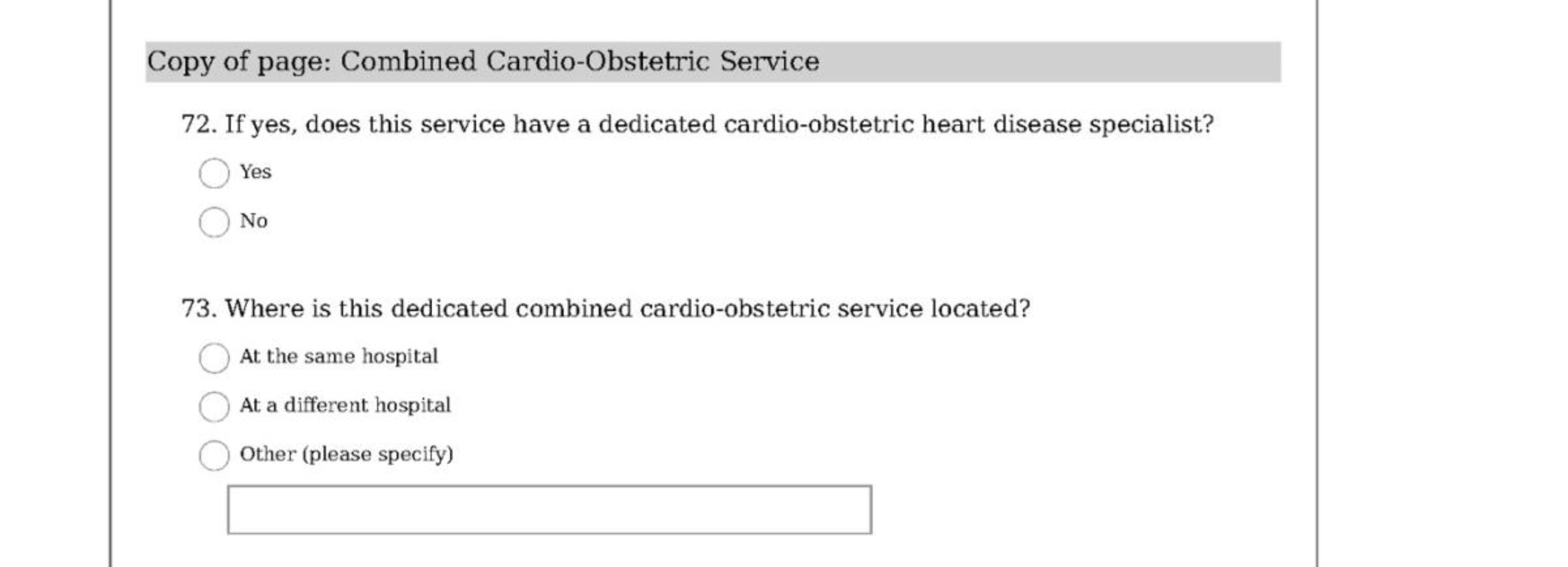


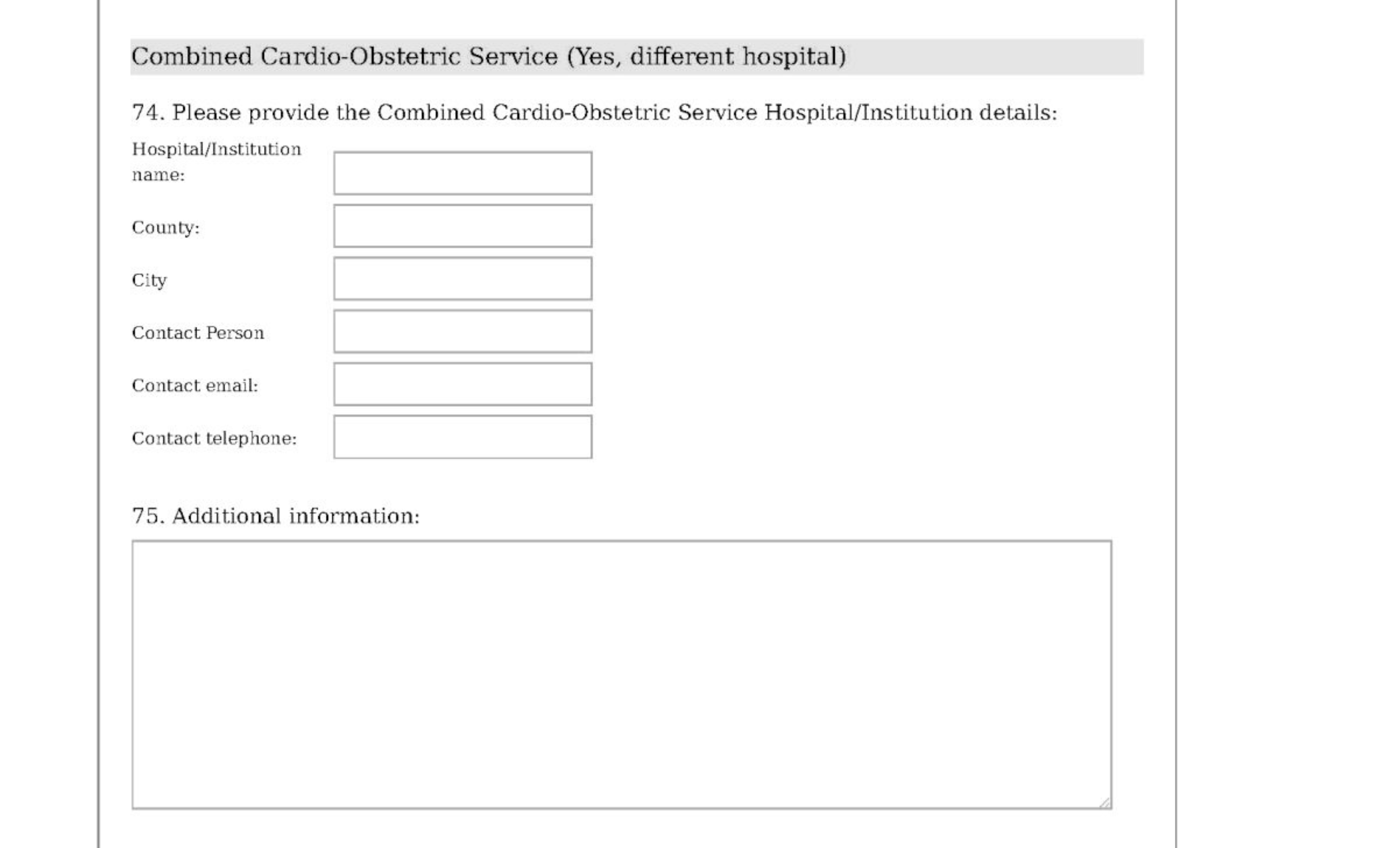


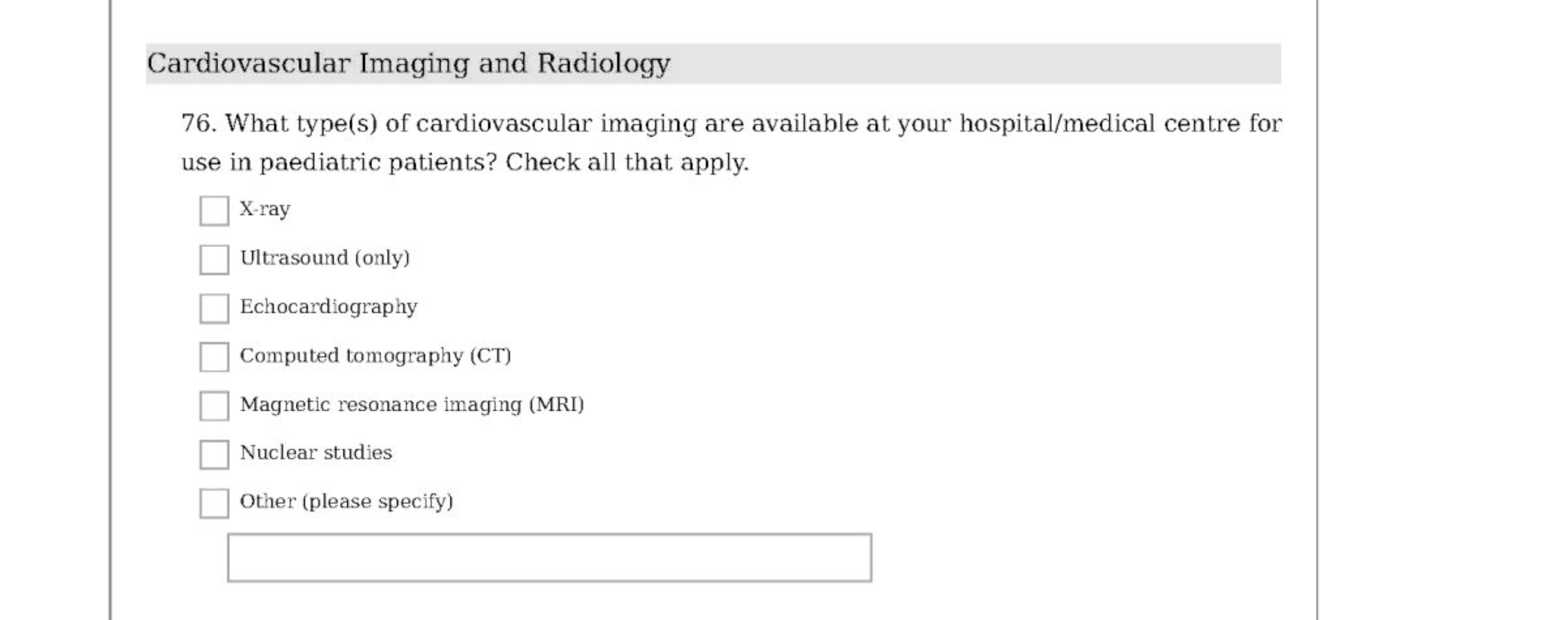


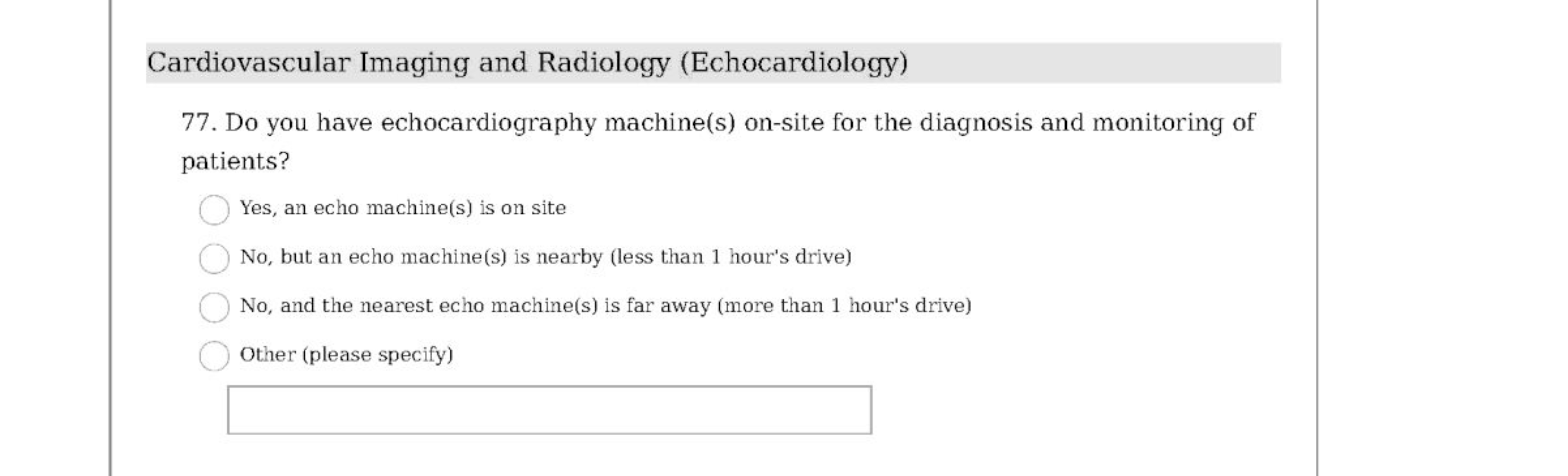


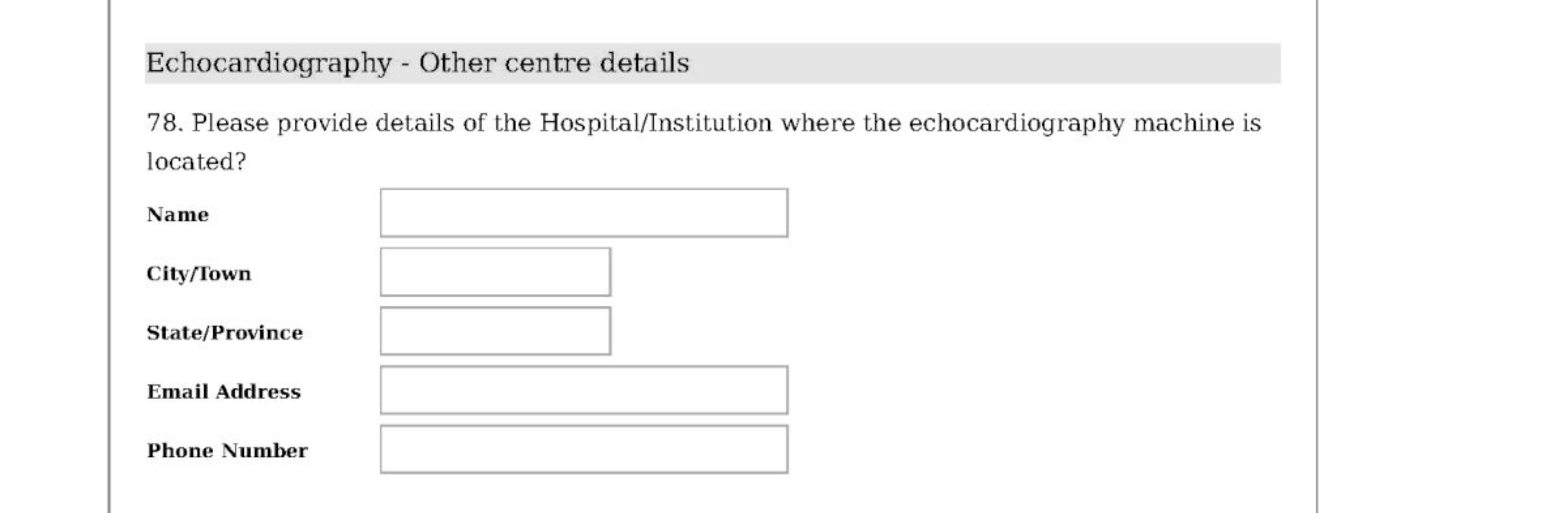


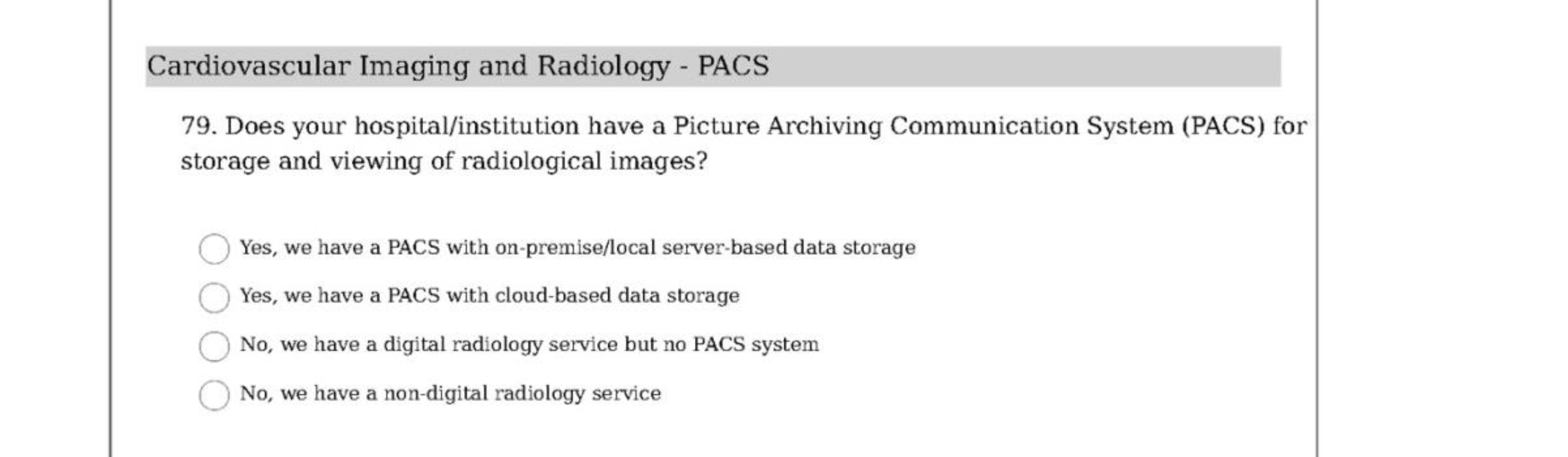


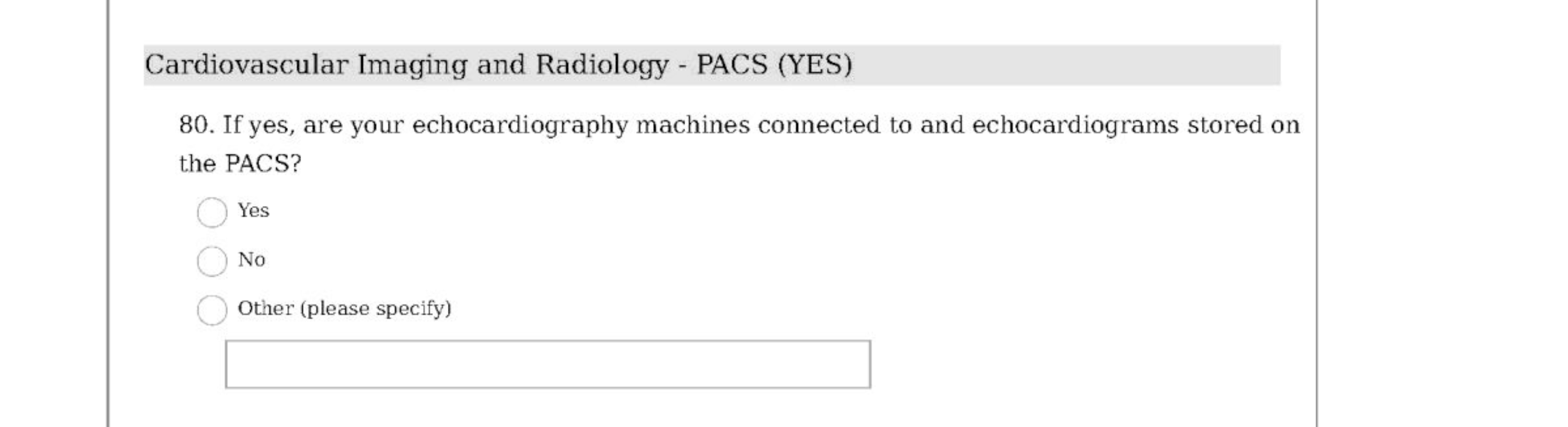


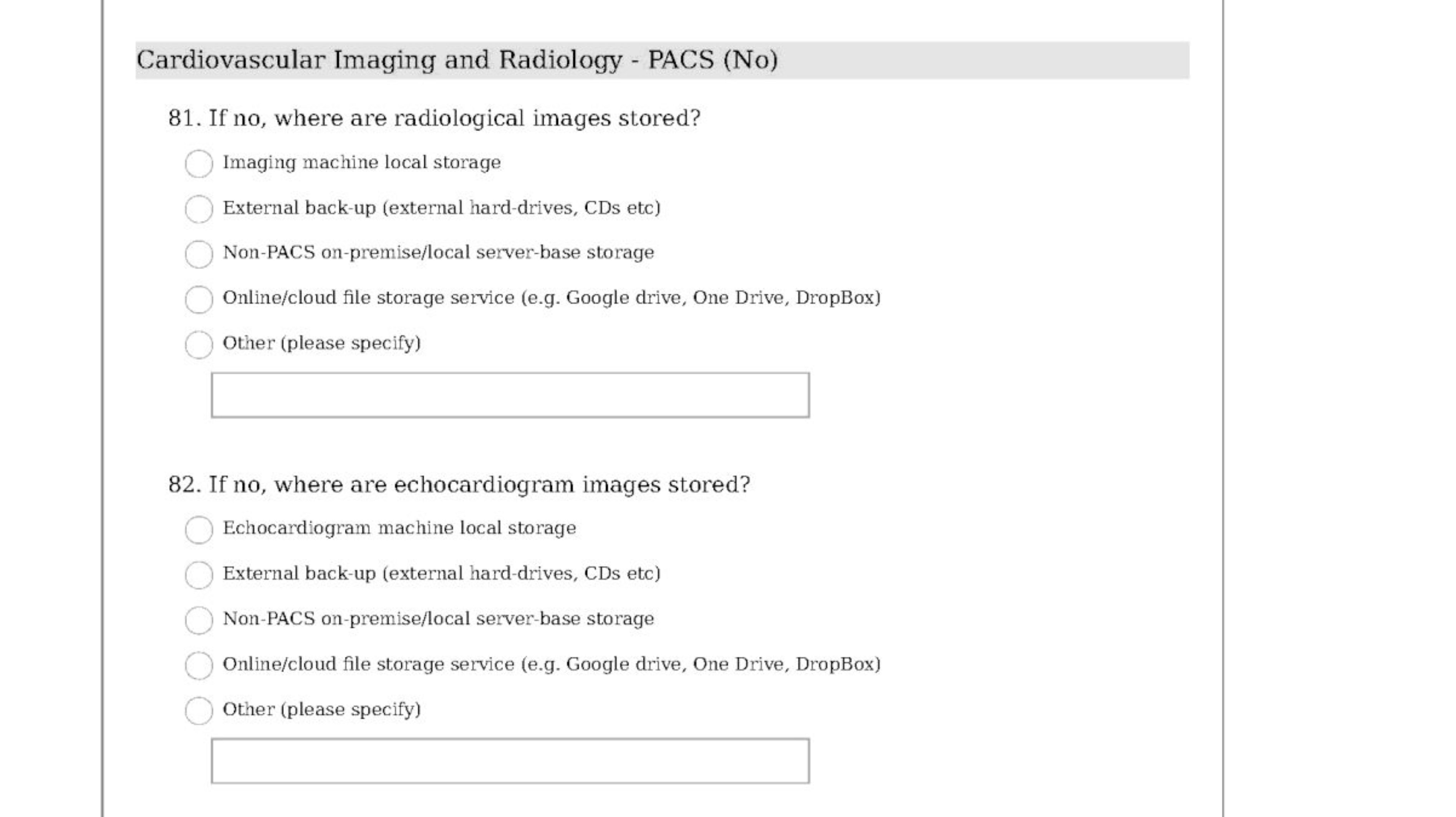


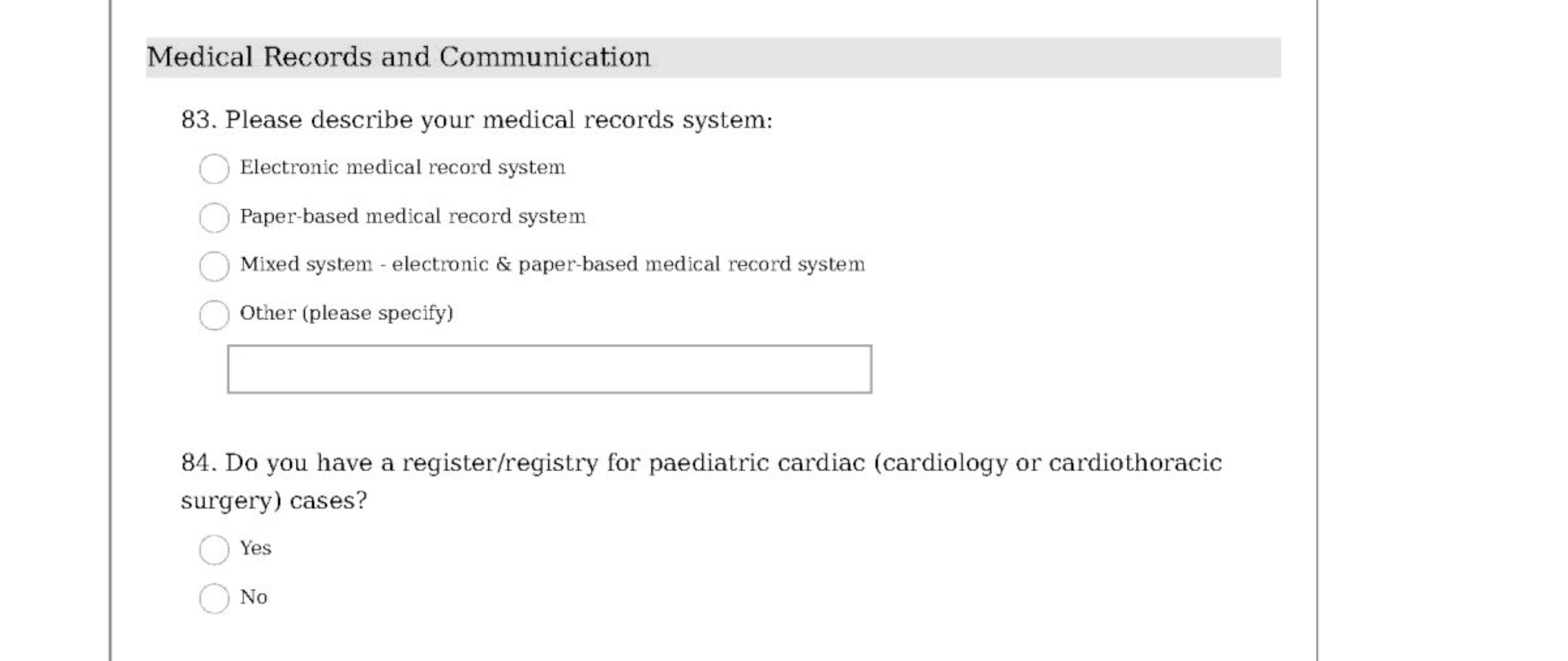


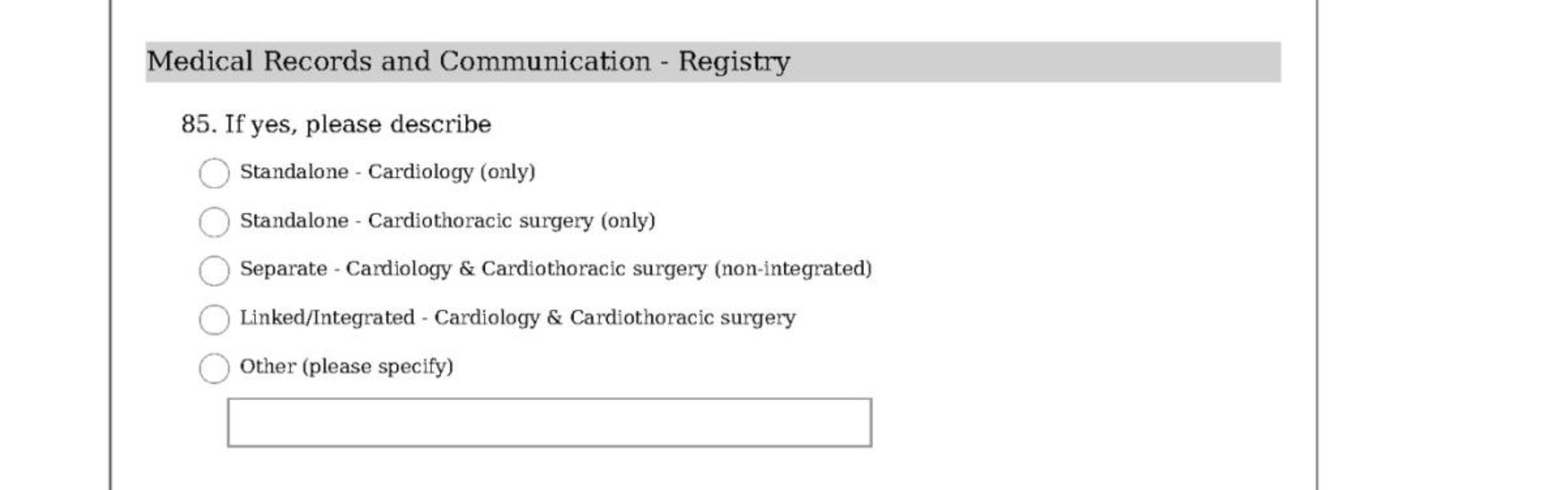


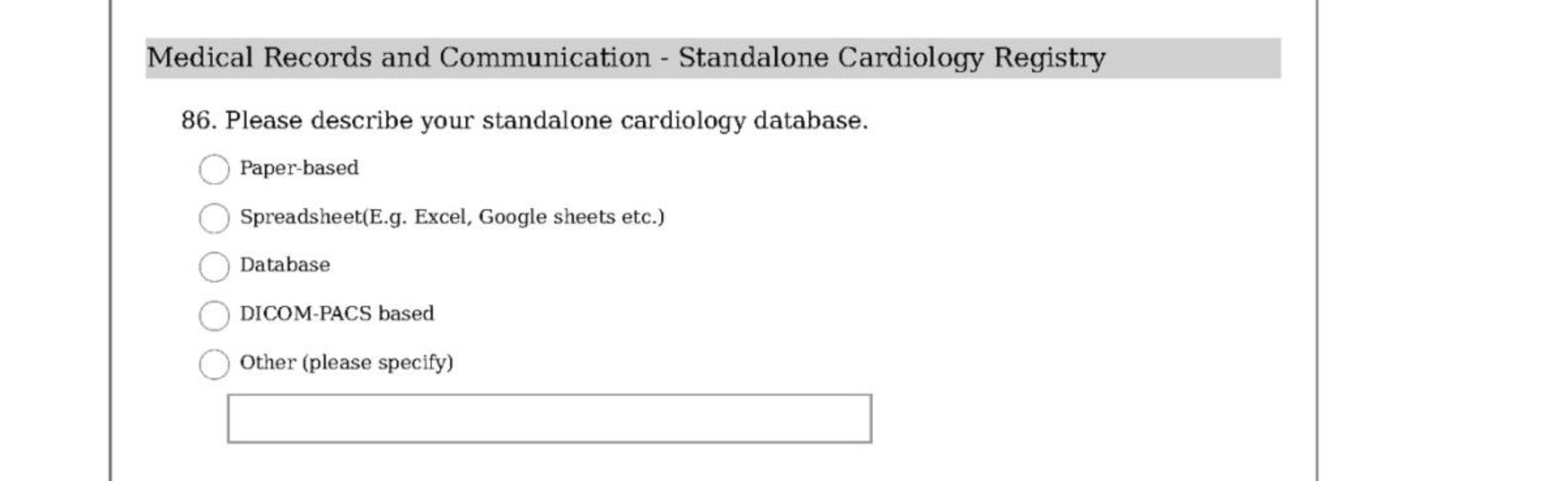


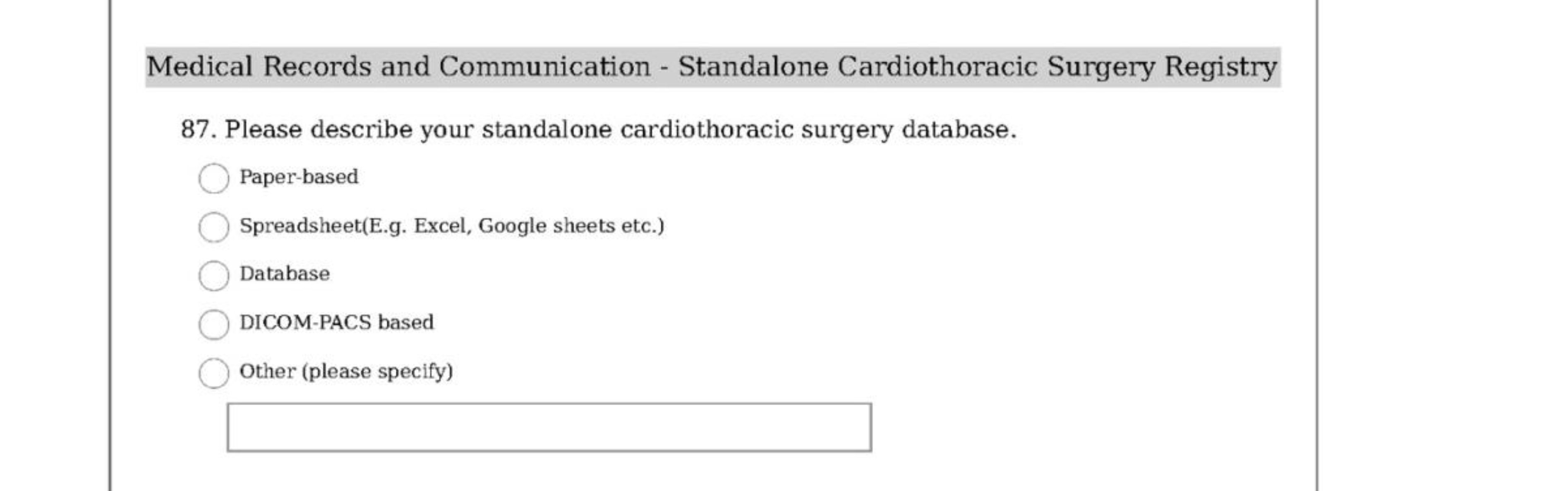


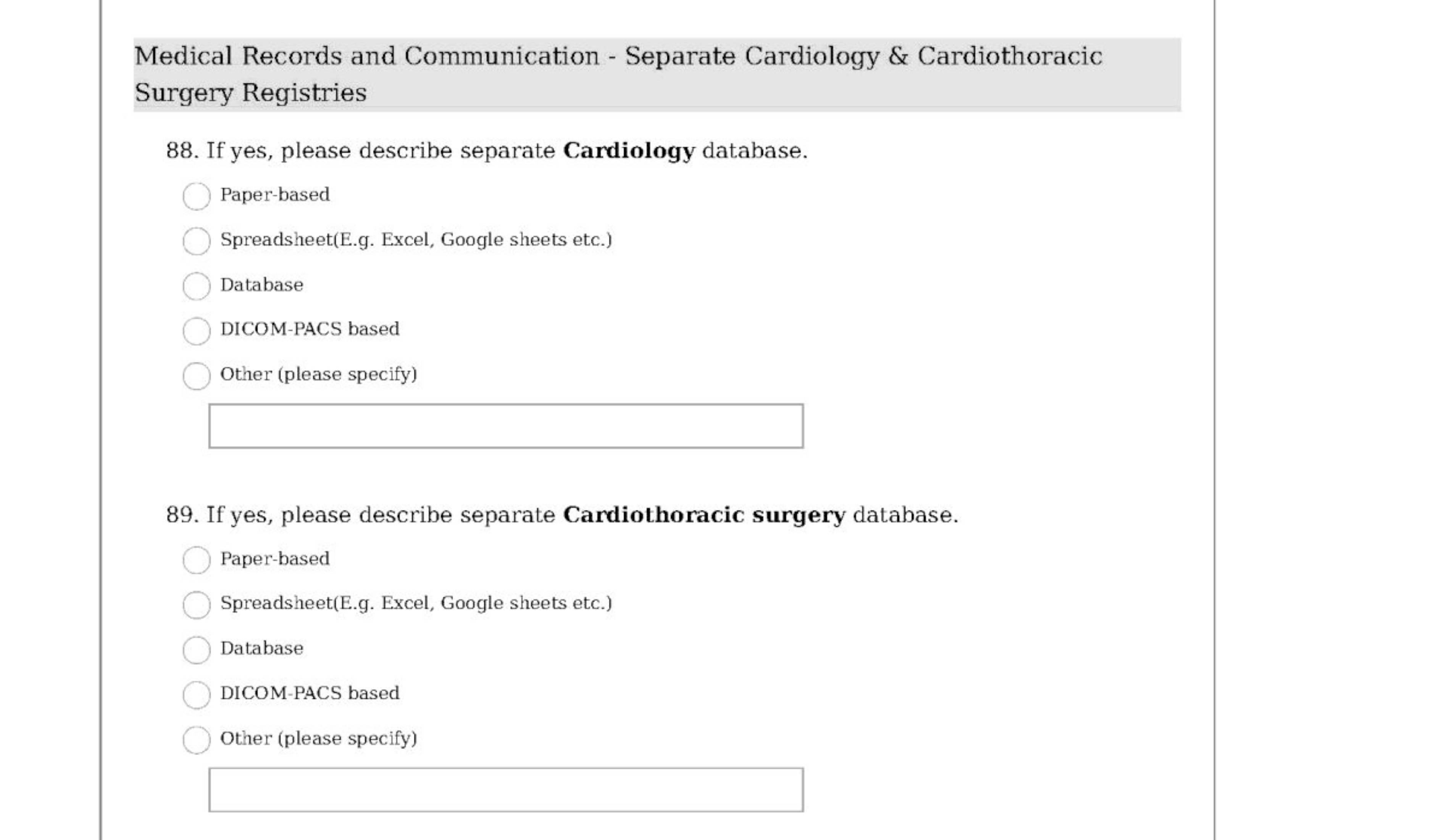


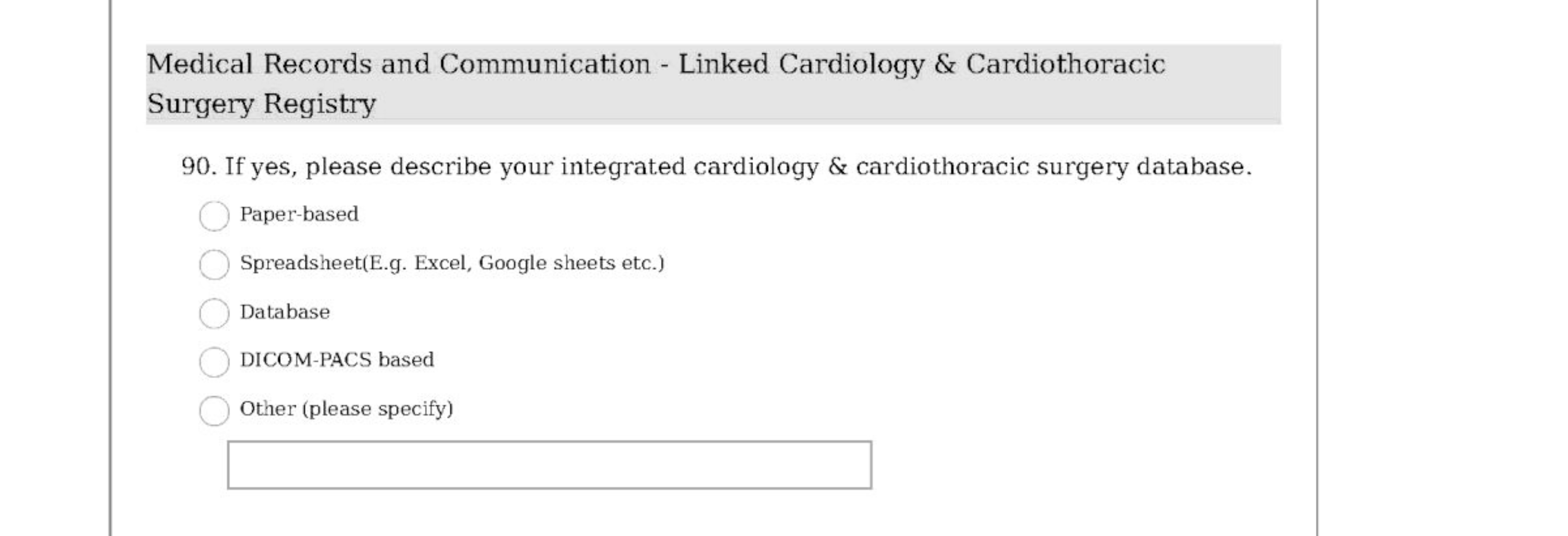


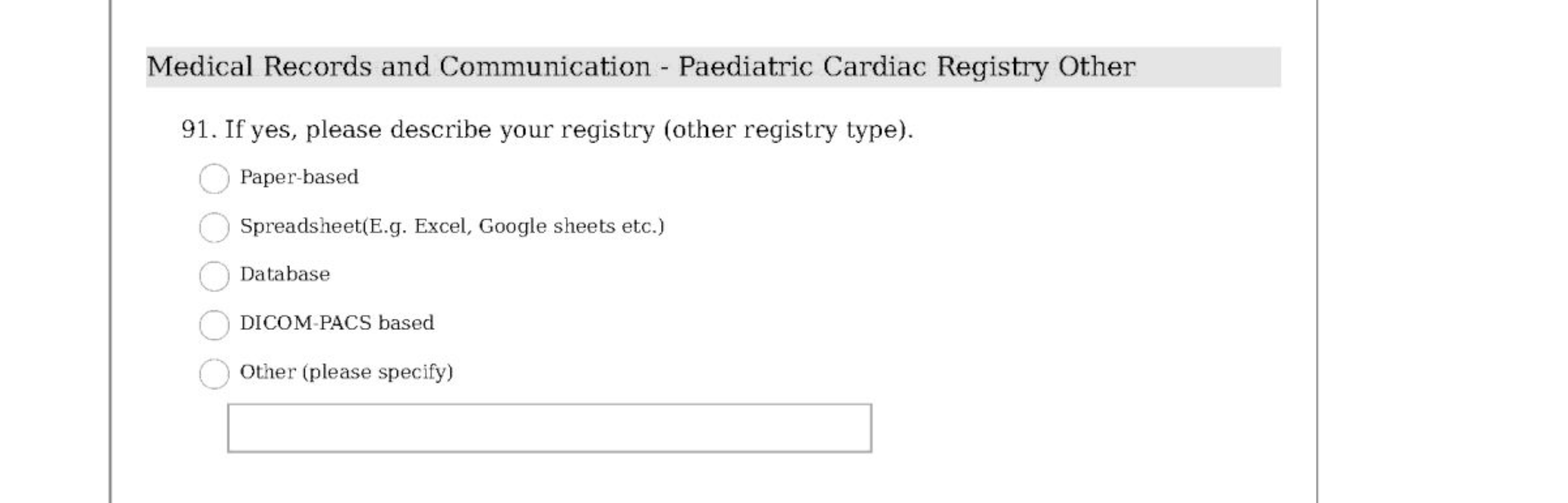


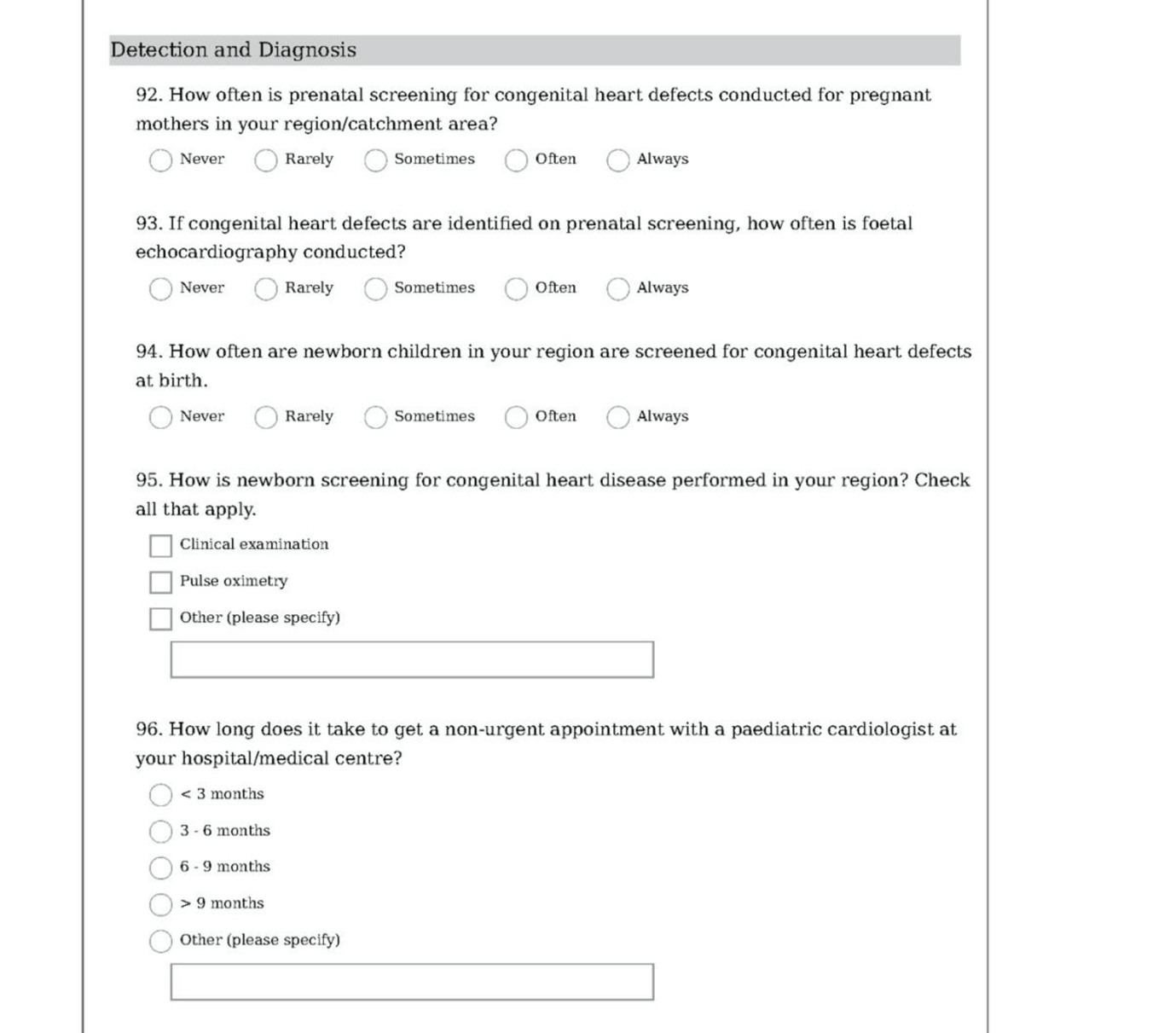


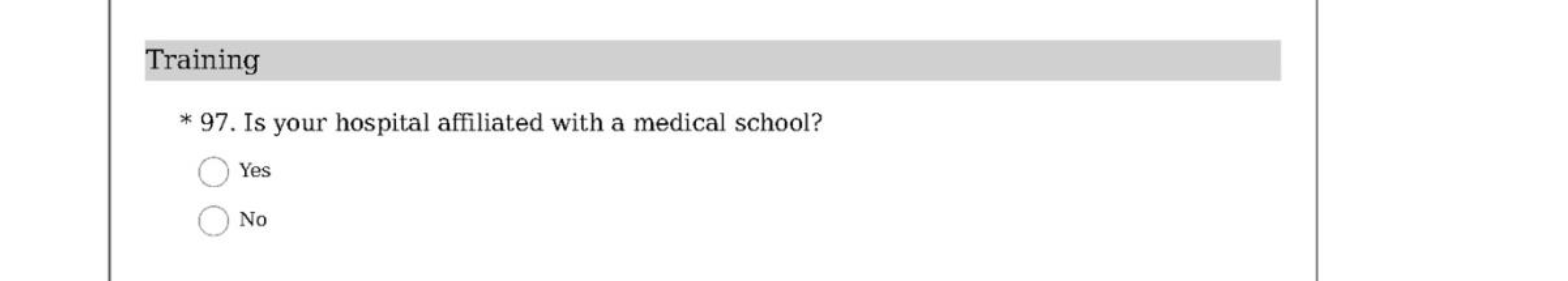


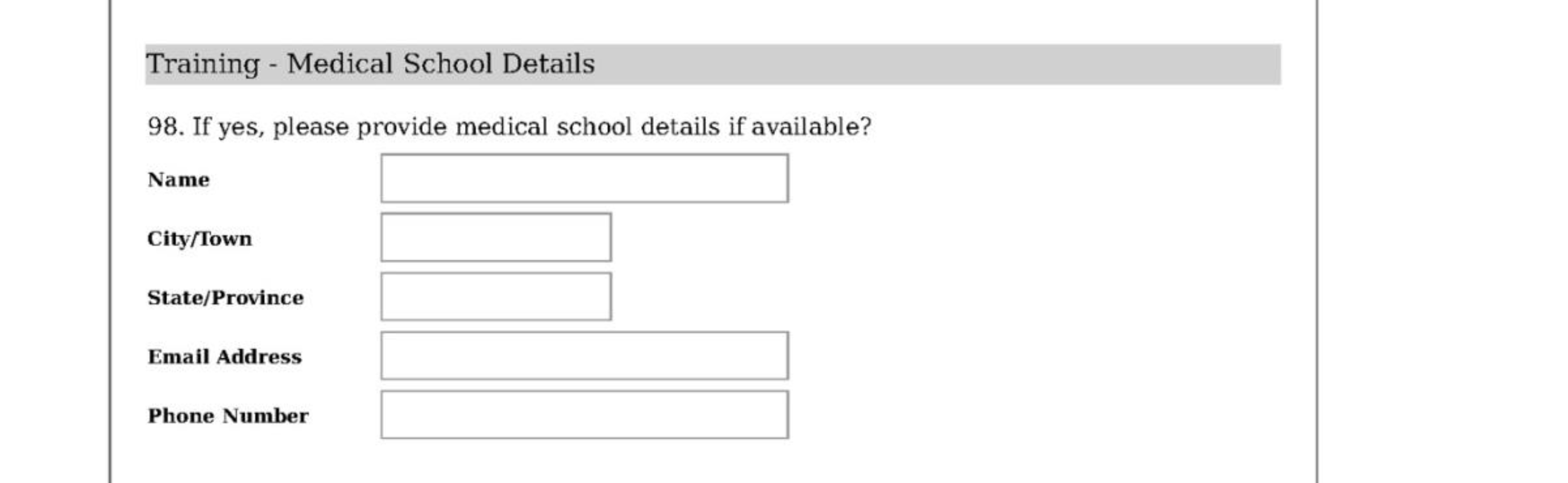


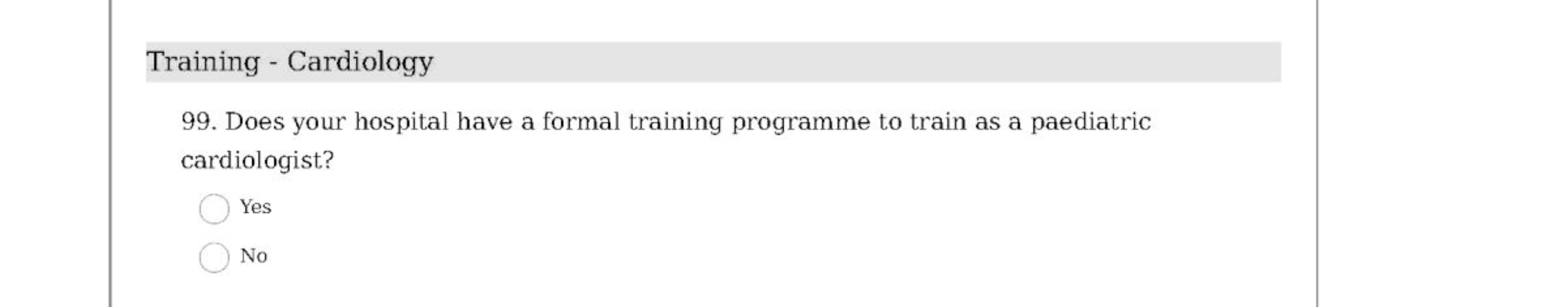


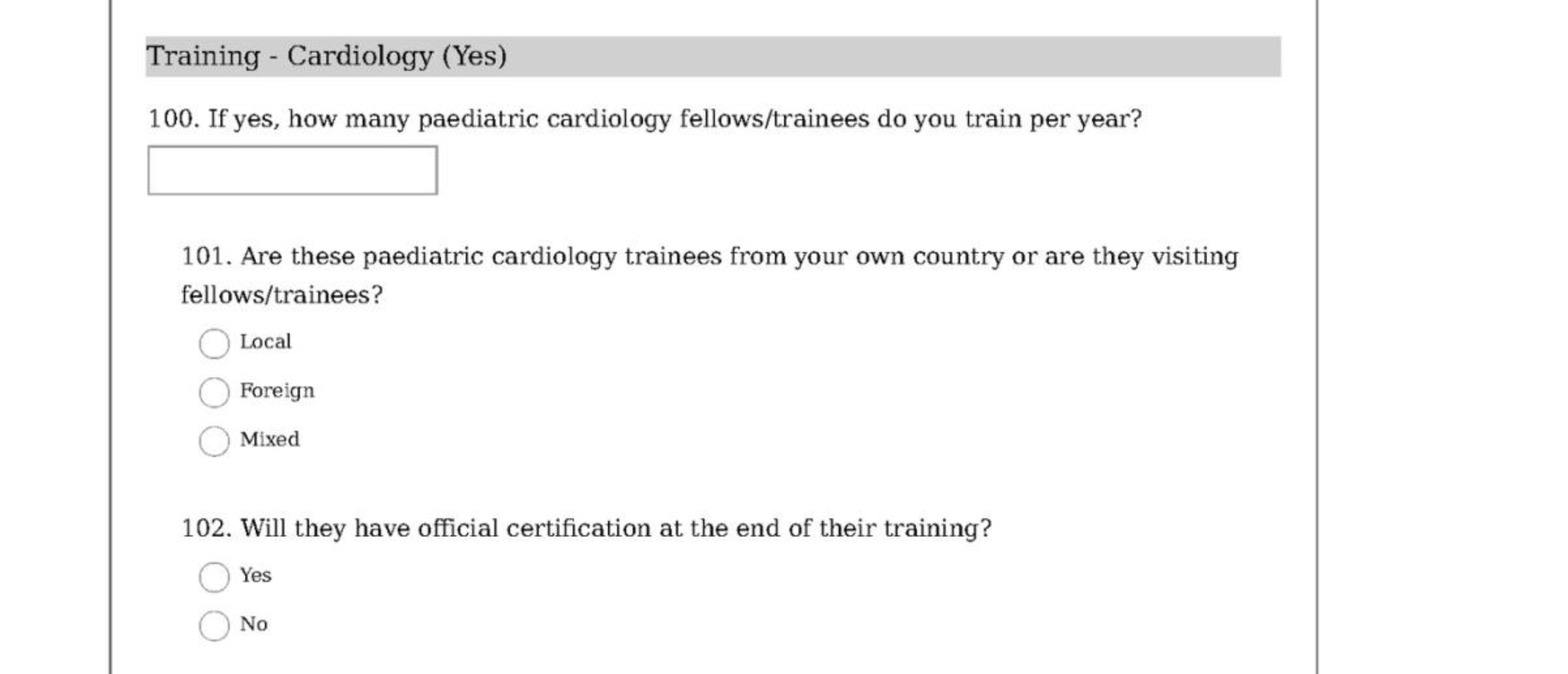


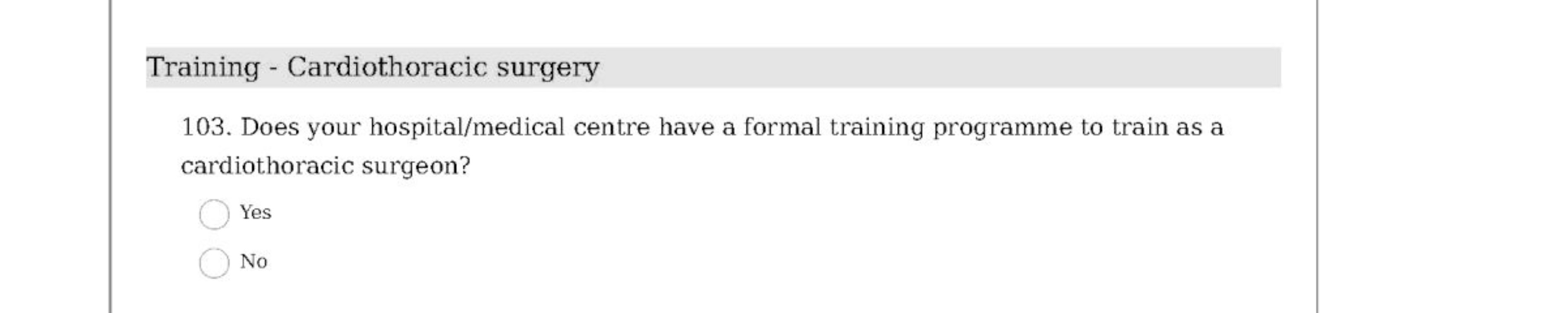


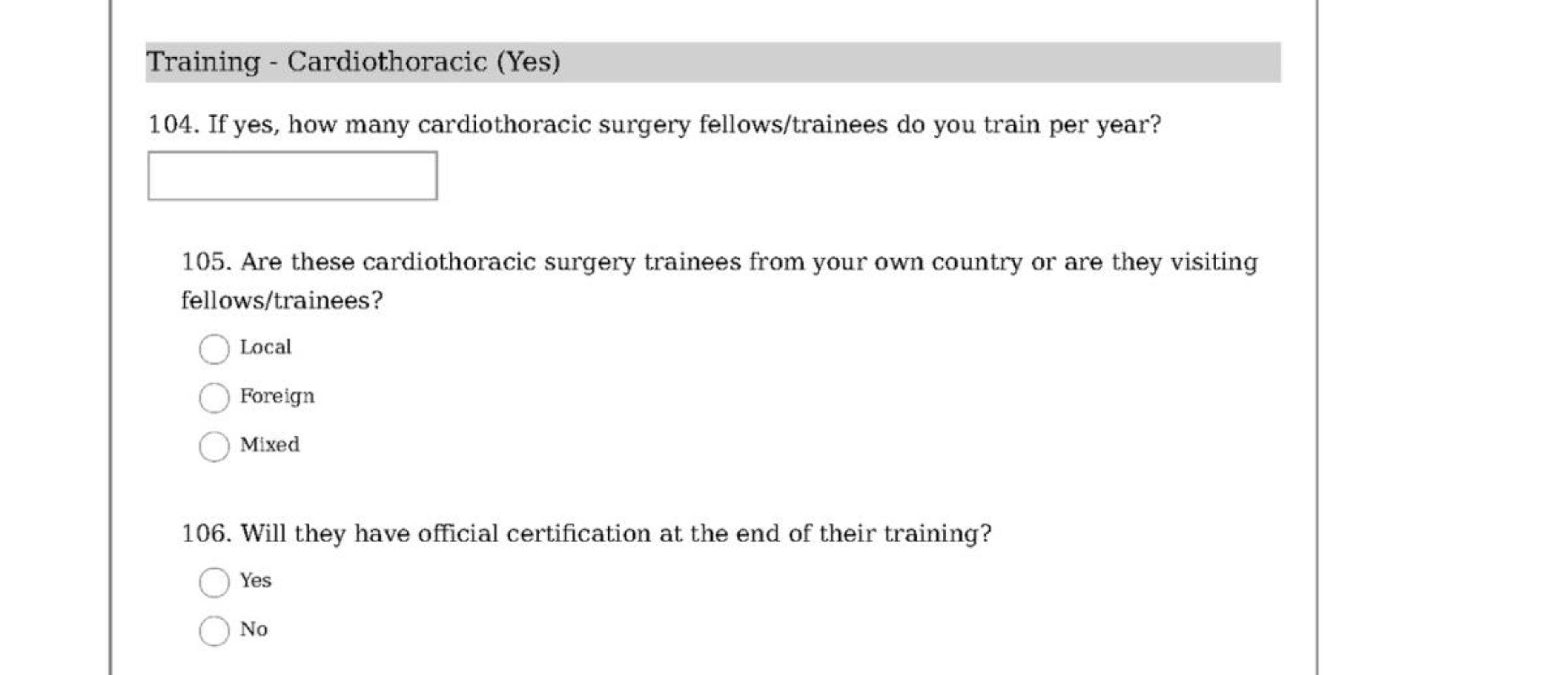


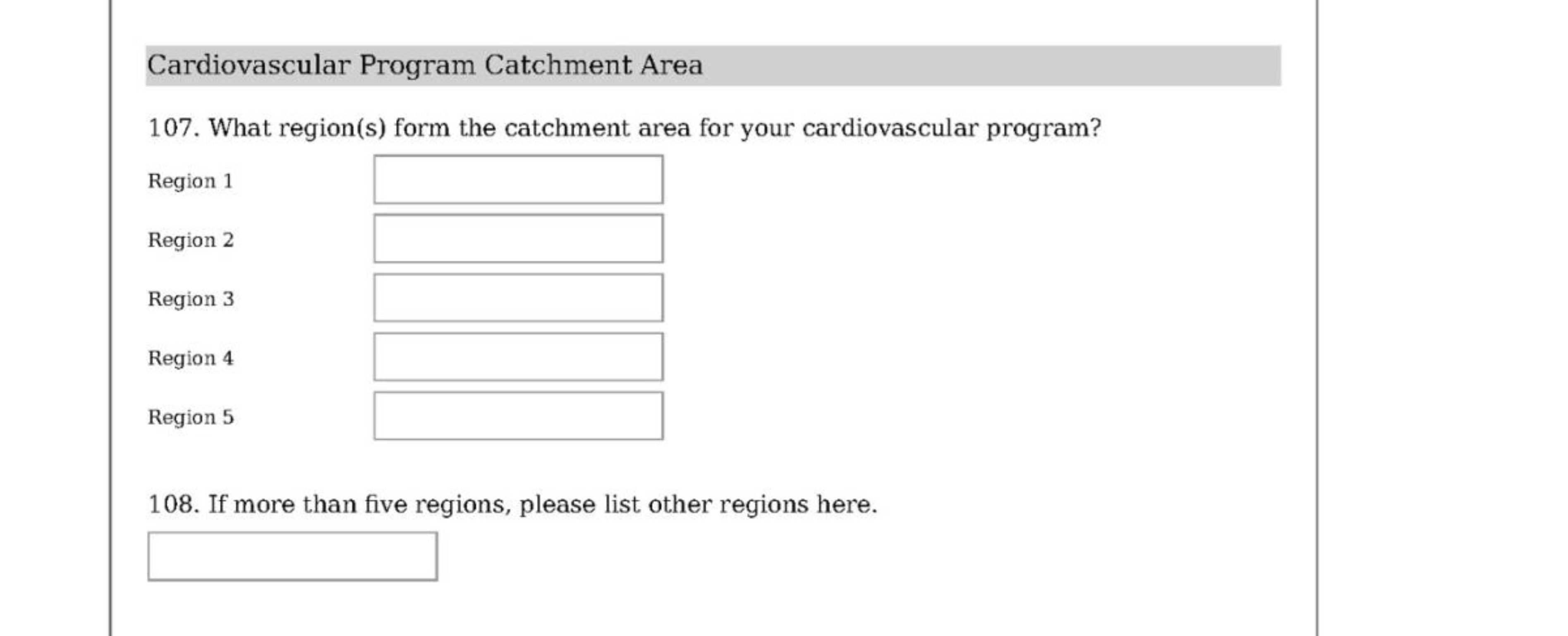


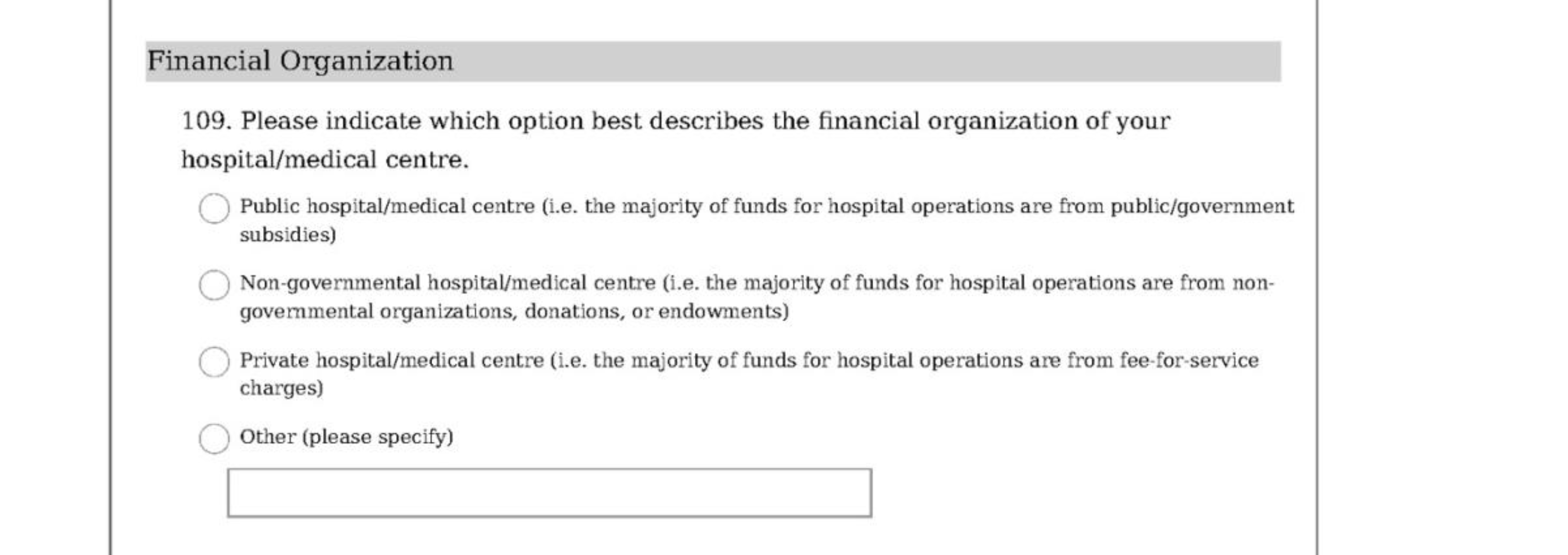


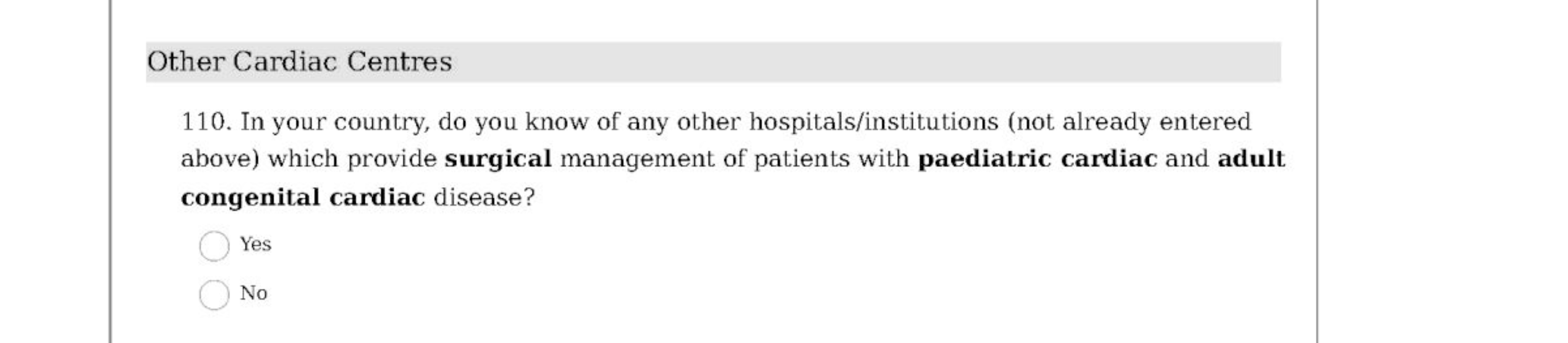


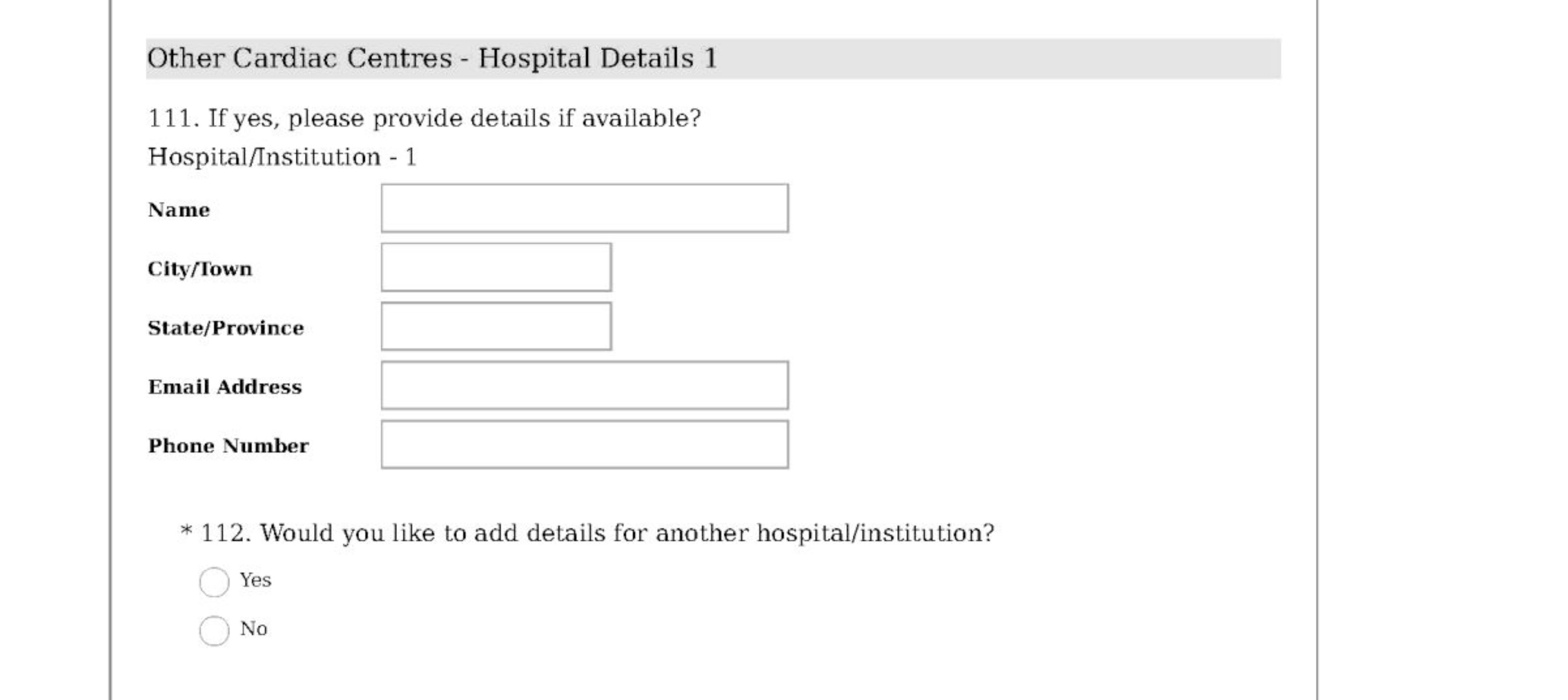


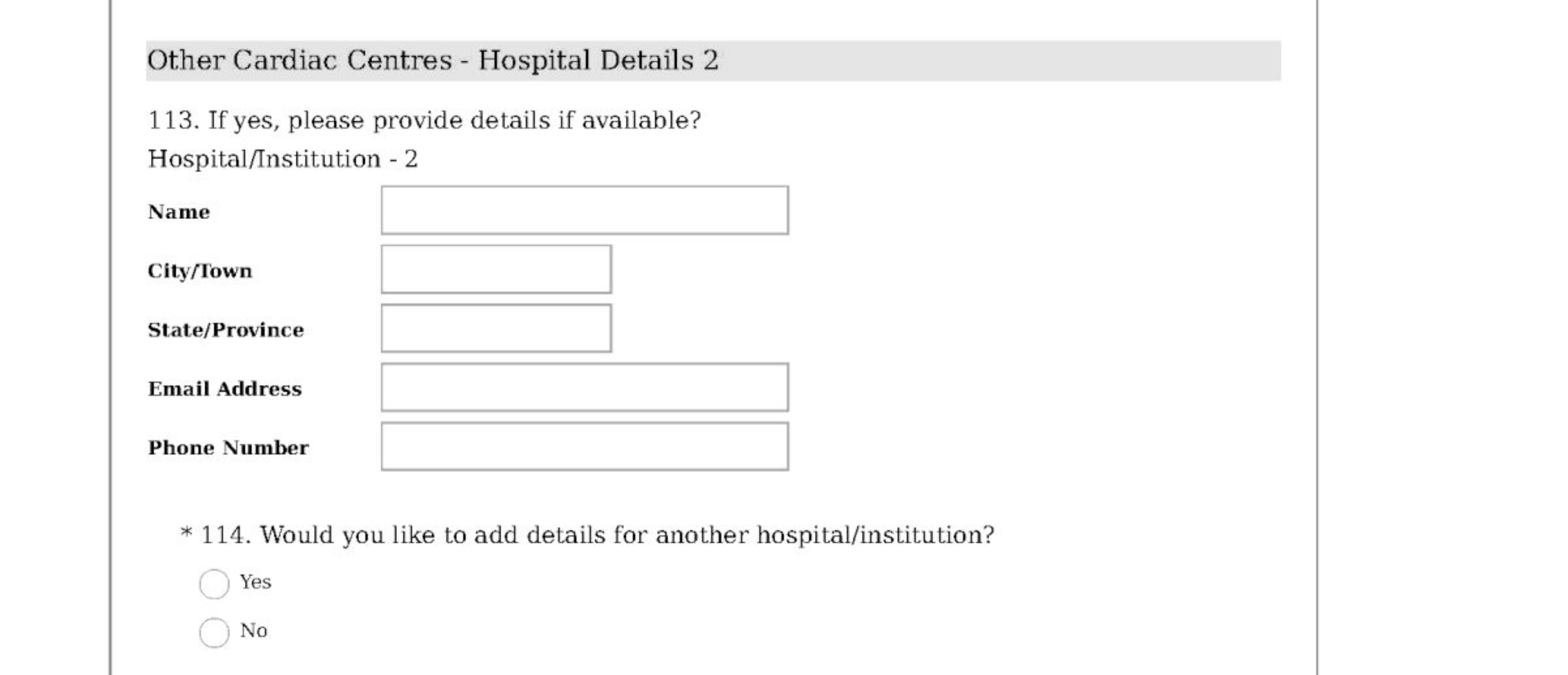


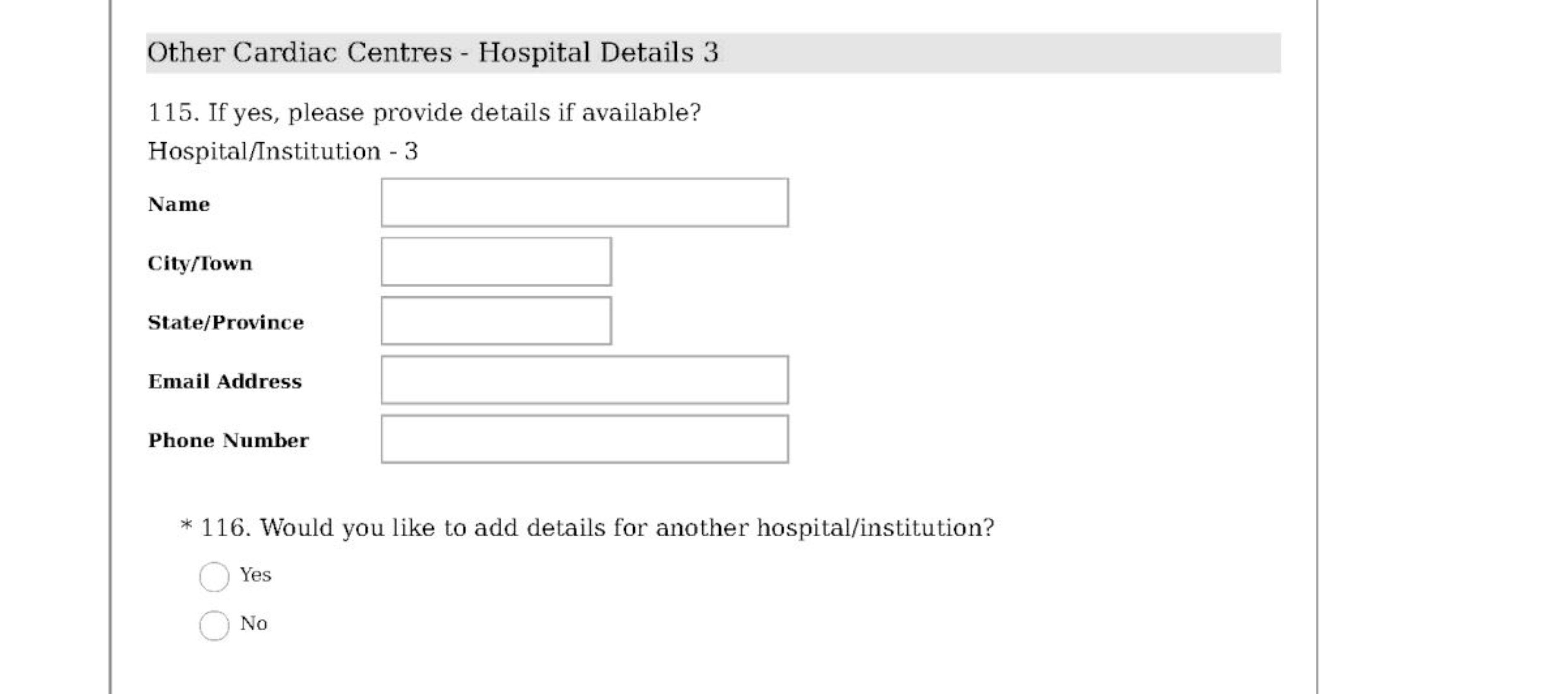


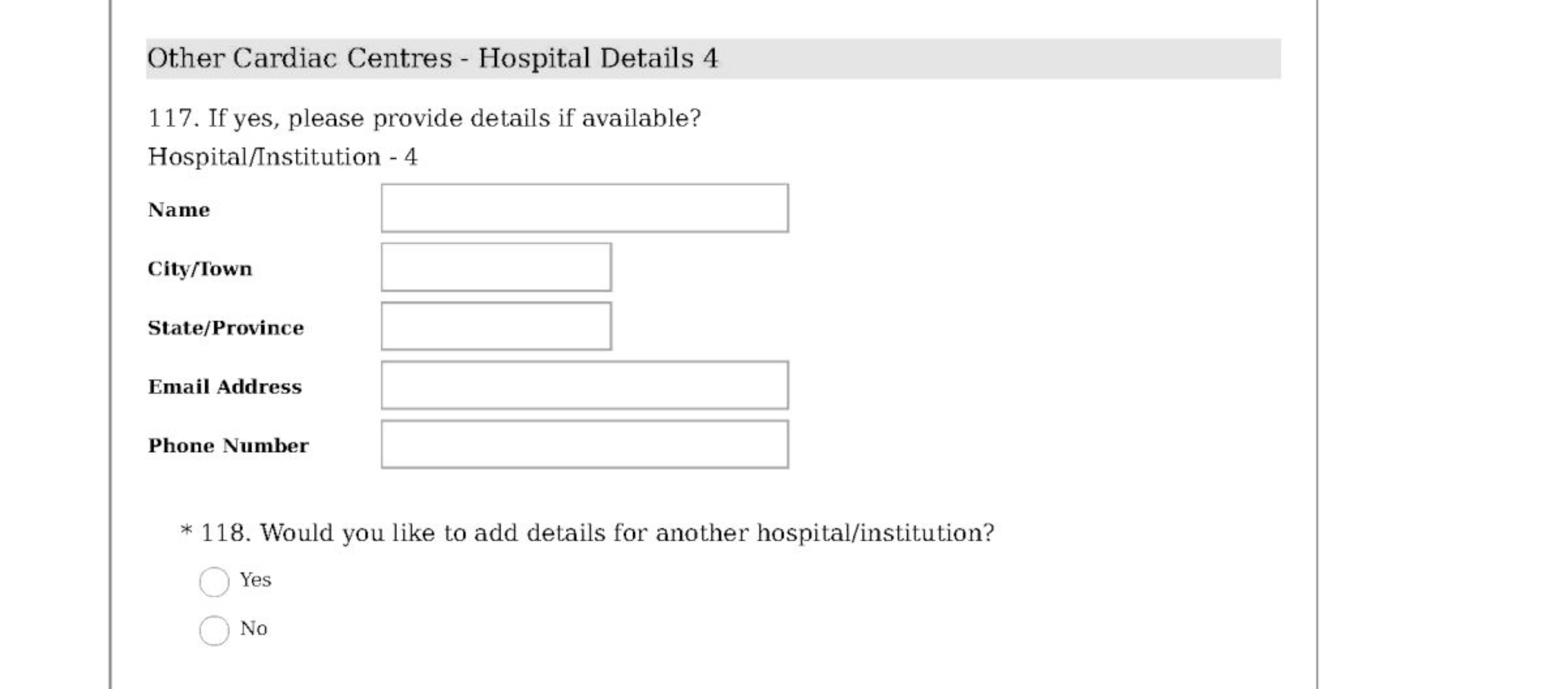


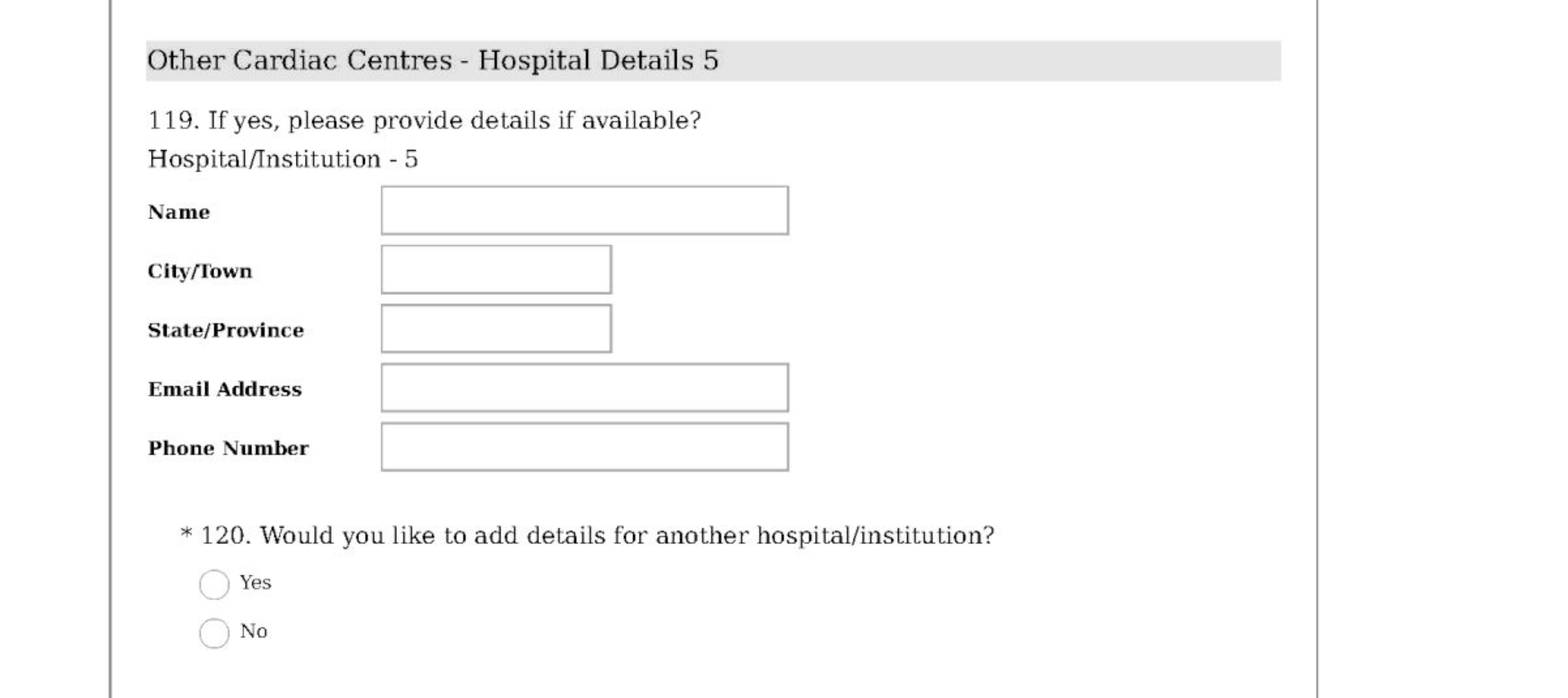


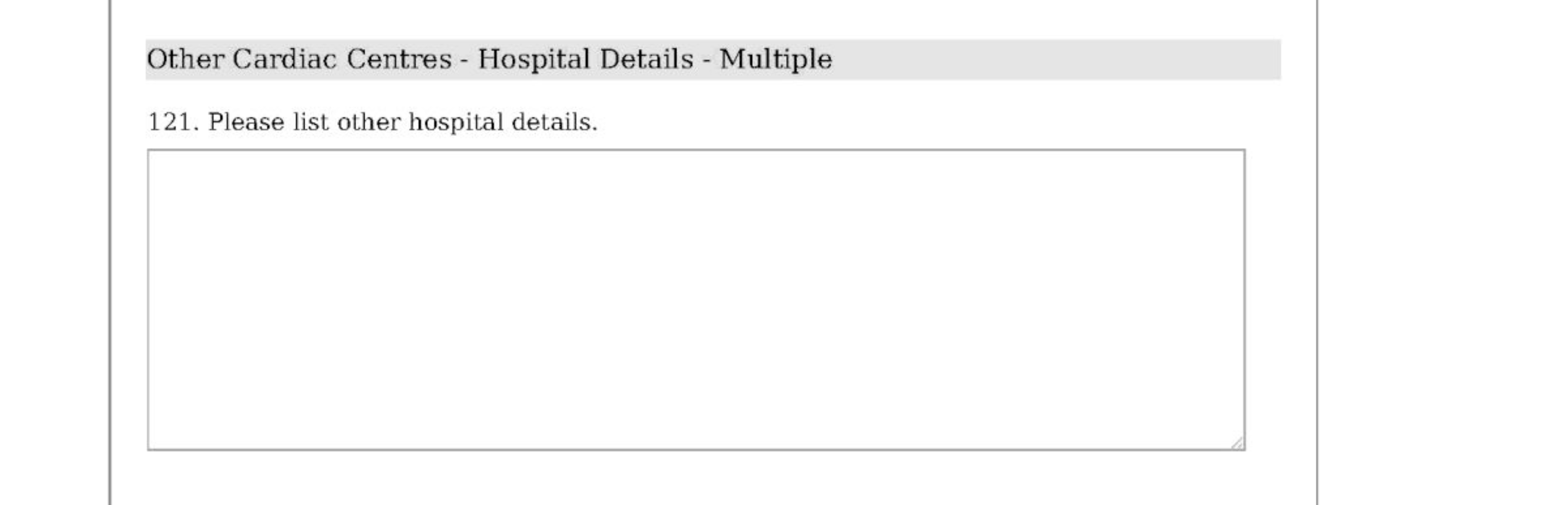


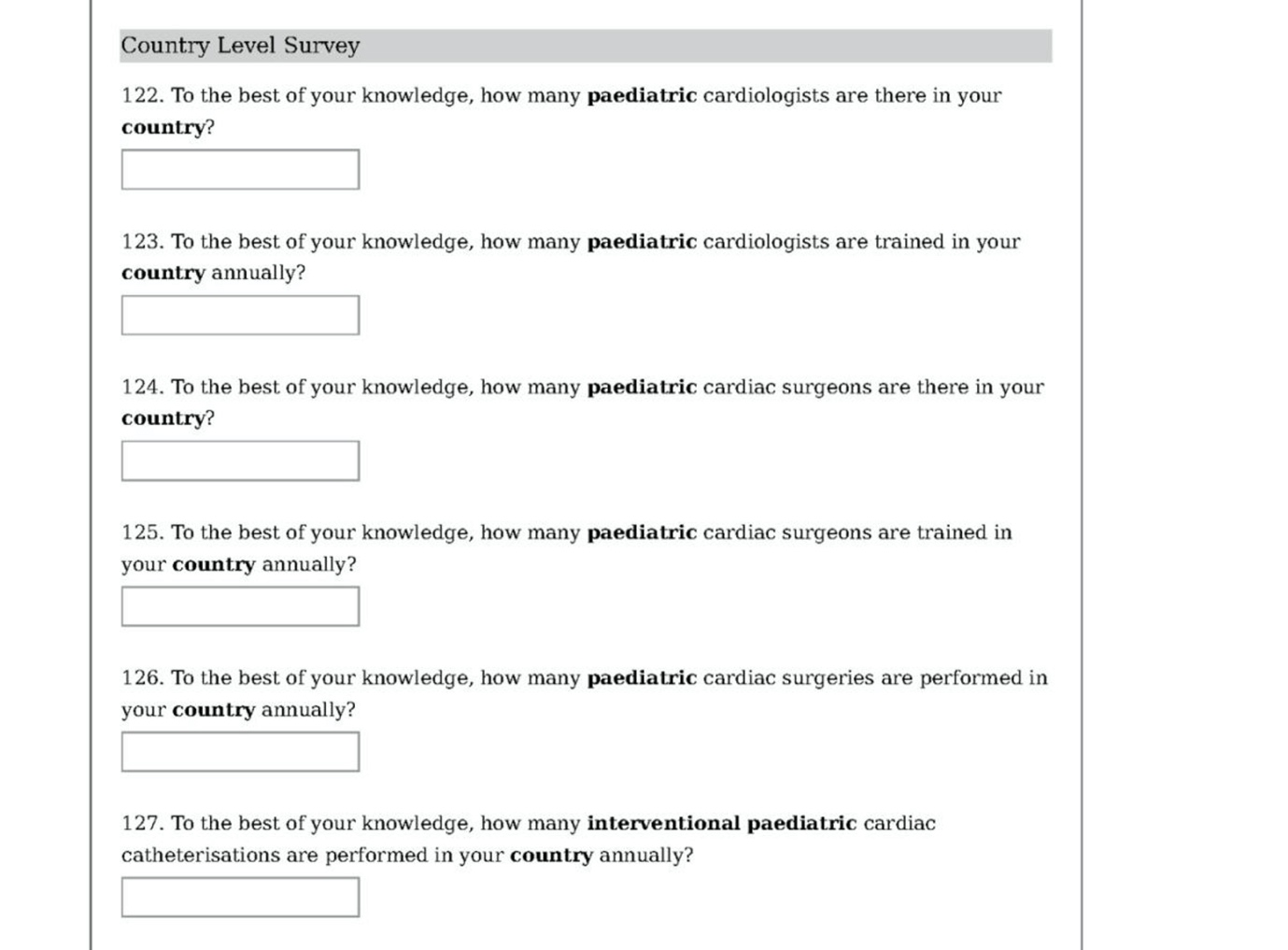


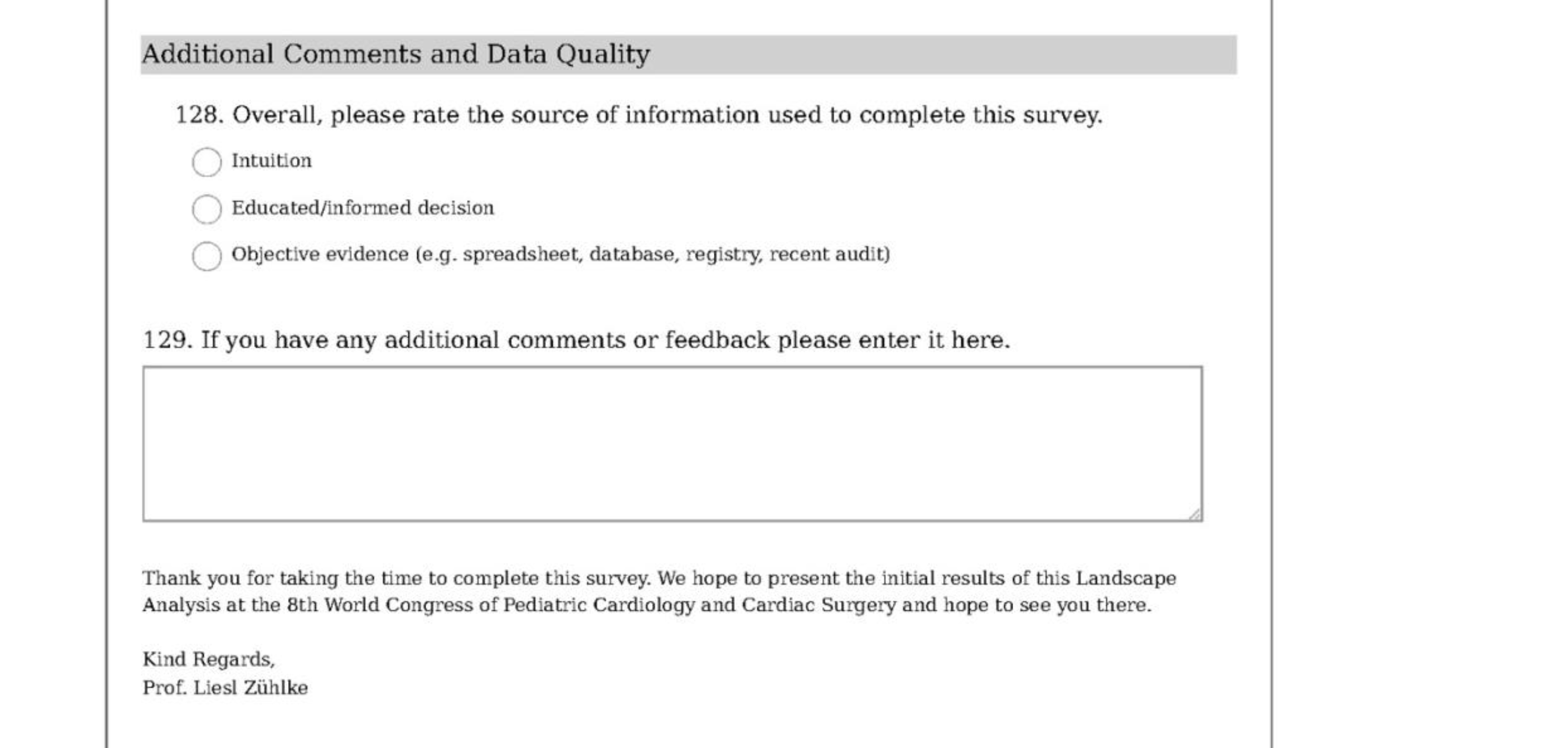

Supplement: sj-docx-1-pch-10.1177_21501351251316230 - Supplemental material for A Landscape Analysis of Pediatric and Congenital Heart Disease Services in Africa [file sj-docx-1-pch-10.1177_21501351251316230.docx]
